# Two-dimensional difference gel electrophoresis (DIGE) analysis of sera from visceral leishmaniasis patients

**DOI:** 10.1186/1559-0275-8-4

**Published:** 2011-05-31

**Authors:** Lokesh A Rukmangadachar, Jitender Kataria, Gururao Hariprasad, Jyotish C Samantaray, Alagiri Srinivasan

**Affiliations:** 1 Department of Biophysics, All India Institute of Medical Sciences, New Delhi, 110029, India; 2 Department of Gastroenterology and Human Nutrition, All India Institute of Medical Sciences, New Delhi, 110029, India; 3 Department of Microbiology, All India Institute of Medical Sciences, New Delhi, 110029, India

## Abstract

**Introduction:**

Visceral leishmaniasis is a parasitic infection caused by *Lesihmania donovani *complex and transmitted by the bite of the *phlebotomine *sand fly. It is an endemic disease in many developing countries with more than 90% of the cases occurring in Bangladesh, India, Nepal, Sudan, Ethiopia and Brazil. The disease is fatal if untreated. The disease is conventionally diagnosed by demonstrating the intracellular parasite in bone marrow or splenic aspirates. This study was carried out to discover differentially expressed proteins which could be potential biomarkers.

**Methods:**

Sera from six visceral leishmaniasis patients and six healthy controls were depleted of high abundant proteins by immunodepletion. The depleted sera were compared by 2-D Difference in gel electrophoresis (DIGE). Differentially expressed proteins were identified the by tandem mass spectrometry. Three of the identified proteins were further validated by western blotting.

**Results:**

This is the first report of serum proteomics study using quantitative Difference in gel electrophoresis (DIGE) in visceral leishmaniasis. We identified alpha-1-acidglycoprotein and C1 inhibitor as up regulated and transthyretin, retinol binding protein and apolipoprotein A-I as down regulated proteins in visceral leishmaniasis sera in comparison with healthy controls. Western blot validation of C1 inhibitor, transthyretin and apolipoprotein A-I in a larger cohort (n = 29) confirmed significant difference in the expression levels (p < 0.05).

**Conclusions:**

In conclusion, DIGE based proteomic analysis showed that several proteins are differentially expressed in the sera of visceral leishmaniasis. The five proteins identified here have potential, either independently or in combination, as prognostic biomarkers.

## Introduction

Leishmaniasis is a vector borne infection caused by the obligate intracellular protozoa belonging to the genus *Leishmania*. It is endemic disease affecting people in the large parts of tropical counties and the Mediterranean basin. Clinically, there are mainly four subtypes, namely cutaneous, muco-cutaneous, visceral (kala azar) and post kala azar dermal leishmaniasis. Visceral leishmaniasis is caused by the *L. donovani *group of organisms and transmitted by the bite of the *phlebotomine *sand fly. There are an estimated 500,000 new cases every year and more than 90% of these cases occur mainly in Bangladesh, India, Nepal, Sudan, Ethiopia and Brazil [1]. The disease has an incubation period between 2 to 6 months and presents with fever, anaemia and enlargement of liver, spleen and lymph nodes. It is almost always fatal if untreated within two years of onset. Diagnosis of the disease is conventionally made by demonstrating the amastigote form of the organism in spleen or bone marrow [2]. Presence of anti-rK39 antibody is also used for the serodiagnosis of leishmania species. The test is highly sensitive but there is a possibility of cross reactivity. Also, the test remains positive even after treatment and may not be useful in immunocompromised patients [3]. The PCR based approaches are expensive, require technical expertise and need to be cost-effective to be useful, especially in areas where visceral leishmaniasis is endemic [4].

Studying the serum protein profile during the infection will yield important information regarding the pathogenesis. This information can be used for identifying biomarkers and therapeutic targets. Detailed proteomic studies of human body fluids in the recent past have resulted in identifying potential biomarker candidates for many conditions [5]. In the past, studies examining the electrophoretic pattern of serum proteins in visceral leishmaniasis have reported decreased serum albumin and increased serum immunoglobulin levels which are used as a supporting evidence for diagnosis [6]. Proteomic techniques have also been used to investigate leishmaniasis in recent studies. Using 2-dimensional western blot analyses with patient's sera and parasites isolated from visceral leishmaniasis patients, immune responses for 330 different leishmania antigens was detected and six antigens were identified [7]. However, there has been no significant study examining the global proteome profile of the visceral leishmaniasis patients' sera. Here, we report, for the first time, the difference-in-gel electrophoresis (DIGE) analysis of sera from visceral leishmaniasis patients and the mass spectrometric identification of the differentially expressed proteins.

## Methods

### Sample collection

The study population included groups of healthy controls and patients with confirmed visceral leishmaniasis. Visceral leishmaniasis serum specimens were collected from the Department of Microbiology, All India Institute of Medical Sciences. Patients were admitted for evaluation of fever when they had fever over a mean duration of two months. All subjects were negative for HIV and HBV. All the serum samples were collected before initiation of the therapy for visceral leishmaniasis. Control serum specimens were from healthy donors. Written informed consent was obtained from all participants before drawing blood. The study was approved by the ethics committee of All India Institute of Medical Sciences and procedures followed were in accordance with the ethical standards formulated in the Helsinki declaration. Serum was separated from 2 ml of blood, aliquoted into separate eppendorf tubes and stored at -70°C.

### Sample processing

Individual samples were treated with Multiple Affinity Spin Cartridge Hu PL 7 kit (Agilent Technologies, USA) according to the manufacturer's instruction for removal of seven high abundant proteins (albumin, IgG, IgA, alpha-1-antitrypsin, haptoglobin, transferrin and fibrinogen). Depleted serum fraction from affinity chromatography was concentrated to a final volume of approximately 100 μl by ultra filtration (5 kDa cut-off). The protein concentration was estimated by Bradford method using bovine serum albumin as standard.

### 2D-DIGE

50 μg of protein from the each of the depleted serum fractions was precipitated with 80% acetone and the precipitate was solubilised in lysis solution (8M urea, 2M thiourea, 4% CHAPS). These samples were labelled with Cy dye flours according to Minimal labelling protocol provided by the manufacturer (Amersham Biosciences, USA). Three patient samples were labelled with Cy3 and three other patient samples with Cy5. Similarly three controls were labelled with Cy5 and three controls with Cy3. This resulted in equal distribution of Cy dyes in both patient and control groups. This dye swapping strategy was adopted to avoid dye bias. Equal amount of protein from all the twelve patient and control samples was mixed to generate an internal standard and 50 μg of protein from this internal standard was labelled with 200 pmol of Cy2. Each gel therefore consisted of one patient (Cy3 or Cy5), one control (Cy5 or Cy3) and one internal standard (Cy2) samples. Labelled serum samples of one patient, one control and one internal standard were pooled together and rehydrating stock solution (8M urea, 2M thiourea, 2% CHAPS, 0.002% bromophenol blue) was added to make up the final volume to 250 μl. DTT and IPG buffer (pH 3-10) were added to a final concentrations of 0.003% and 0.5% respectively. After 15 h of rehydration, IPG strips (13 cm, pH 3-10) were subjected to iso-electric focusing in an Ettan IPGphor 3 system (Amersham Biosciences, USA) for a total of 27,000 Volt-hours. Each electro focused strip was equilibrated, first with 10 ml of SDS equilibration buffer containing 10 mg/ml DTT for 15 minutes. This was followed by second equilibration with SDS equilibration buffer containing 25 mg/ml iodoacetamide for 15 minutes. The strips were then transferred onto 10% homogenous polyacrylamide gels cast on SE 600 Ruby gel apparatus (Amersham Biosciences, USA). The strips were overlaid with 0.5% agarose sealing solution (0.5% agarose, 0.002% bromophenol blue in Tris-glycine electrode buffer). Separation in SDS-PAGE was carried out with constant running current set at 15 mA per gel at 20°C for 30 minutes, followed by 30 mA per gel at 20°C until the bromophenol blue dye front ran off from the bottom of the gels. Six such gels were run corresponding to six biological replicates.

### Image acquisition and analysis

Labelled proteins were visualized using a Typhoon TRIO Variable Mode Imager (Amersham Biosciences, USA). Cy2 images were scanned with 488 nm/520 nm, Cy3 images were scanned with 532 nm/580 nm and Cy5 images were scanned with 633 nm/670 nm. All gels were scanned with a PMT setting of 750 to 800 V with 200 μm/pixel resolution. Images were cropped using Image-Quant™ v 5.5 (Amersham Biosciences, USA) to remove areas extraneous to the gel image. Gel images were processed using DeCyder™ 2D version 7.0 (Amersham Biosciences, USA). The images were imported to Differential in-Gel Analysis (DIA) workspace to create six different workspaces for each of the six gel pairs. The maximum number of spots for each co-detection procedure was set to 1500. The spots were co-detected and quantified automatically as 2-D DIGE image pairs, intrinsically linking the samples to its in-gel standard. These six DIA workspaces were then analyzed in the Biological Variation Analysis (BVA) workspace. In BVA work space, each Cy3 or Cy5 gel image was assigned an experimental condition, either control or visceral leishmaniasis and all Cy2 images were classified as standards. The gel with the highest spot count was assigned as the master gel. Matching between gels was performed utilizing the in-gel standard from each image pair. Matching was further improved by land marking and manually confirming potential spots of interest. Student t-test was performed for every matched spot-set, comparing the average and standard deviation of protein abundance for a given spot.

### Mass spectrometric analysis and protein identification

A preparative gel was run using 500 μg of pooled protein sample and stained with colloidal coomassie blue. Matched spots of interest were picked manually from the preparative gel. These spots were subjected to in-gel trypsinization according to the manufacturer's protocol (Promega, USA). After overnight digestion, digestion buffer containing the peptides was recovered. Additional extraction of peptides was carried out with 100 μl of 50% acetonitrile in 1% formic acid. The extracts were poled and vacuum-dried. For LC-MS/MS, peptide mixtures were resuspended in 50% acetonitrile and 1% formic acid solution and analyzed in a *Tempo*™ nano-LC system (Applied Biosystems) coupled to QSTAR XL system (*Applied Biosystems, USA*). Some spots were analyzed by off-line nanospray method. These peptides were dissolved in 20 μl of 50% acetonitrile in 0.1% formic acid. Nanospray ionization was carried out using an ion spray voltage of 900. The spectra were acquired in an information dependent manner utilizing the Analyst QS 2.0 software to generate raw data. Database searching was done using Mascot search program (Version 1.6, Matrix Science, UK). Search parameters were as follows: 1 missed cleavage allowed, carbamidomethylation set as fixed modification, methionine oxidation as variable modification, peptide mass tolerance ± 1.2 Da, fragment mass tolerance: ± 0.6 Da, monoisotopic mass values. Spectra were searched against NCBInr or MSDB database. Criteria for positive identification were a significant Mascot probability score (score >40; p < 0.05).

### Western Blot analyses

Individual undepleted serum specimens were separated on 12% polyacrylamide gels and transferred onto nitrocellulose membranes in a trans-blot electrophoresis transfer cell (Bio-Rad, USA). Western blot analyses were performed by using polyclonal antibodies against C1 inhibitor (diluted 1:200, Santa Cruz, USA), transthyretin (diluted 1:2500, Abcam, USA) and apolipoprotein A-I (diluted 1:10000, Abcam, USA). Peroxidase-conjugated antibody (diluted 1:5000, Abcam, USA) was used as secondary antibody. The reaction was detected by chemiluminescence with ECL reagents (Pierce Biotechnology, USA). A semi quantitative analysis based on optical density was performed by ImageJ software (available at http://www.rsbweb.nih.gov/ij). Student t-test was used to determine mean differences between two groups and a p < 0.05 was considered significant at a 95% confidence level.

## Results

### Clinical data

The clinical data from the study subjects are summarised in Table 1. Analysis of the age distribution between the two groups showed that there was no significant difference among the patient and controls in both the DIGE study group and the validation groups (p value > 0.05). Patients in DIGE study group had an established diagnosis of visceral leishmaniasis as evidenced by the presence of the parasite in the bone marrow aspirates and presence of anti rK39 antibody. Validation cohort consisted of patients with clinical diagnosis of visceral leishmaniasis supported by the presence of anti rK-39 antibody.

**Table 1:** Clinical Data of study subjects

*Group*	*No*	*Age in years (Mean ± SD)*	*Sex Male/Female*	*Presence of parasite in bone marrow*	*Presence of anti rK 39 antibody*
*2D DIGE*					
Visceral leishmaniasis	6	27.8 ± 18.3	6:0	6/6	6/6
Controls	6	27.6 ± 2.7	4:2	NA^a^	0/6
*Validation*					
Visceral leishmaniasis	19	25.2 ± 10.8	17:2	9/19	19/19
Controls	10	26.9 ± 4.5	8:2	NA^a^	0/19

^*a *^*NA *Not Applicable

### 2D-DIGE and Protein Identification

**Figure 1:**
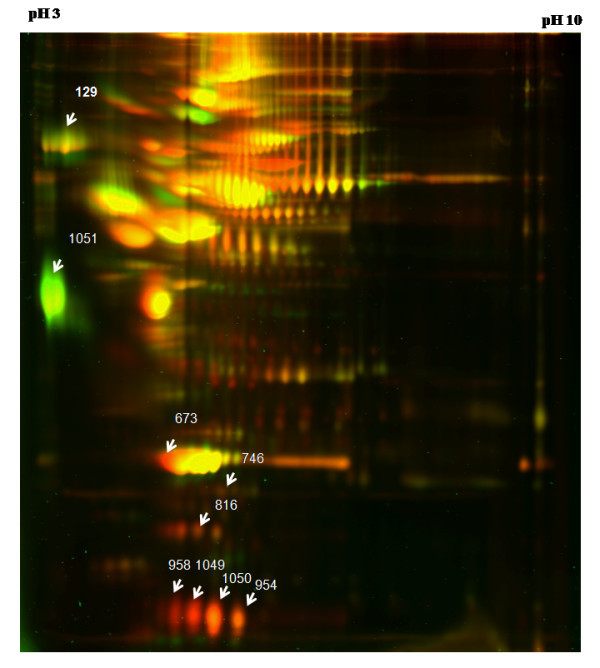
**Analysis of serum proteome by DIGE**. A representative DIGE image (grey scale) showing the serum protein profile. Proteins identified as differentially expressed are shown by arrows with numbers assigned in the DeCyder analysis. Patient and control sera were labelled with Cy3 and Cy5 respectively in this gel. The range of the horizontal dimension is isoelectric point (from pI = 3 to pI = 10); the range of the vertical dimension is molecular weight (from approx. 150 to 10 kD)

Immunodepleted serum proteome profiles of six visceral leishmaniasis patients and six healthy volunteers were compared using DIGE. Three images corresponding to the three samples (control, visceral leishmaniasis and internal standard) were generated for each gel. Eighteen images were generated in total corresponding to the six gels. A representative DIGE gel showing the overlay of Cy3 and Cy5 images from one such gel is shown in Figure 1. Between 894 to1051 spots were co-detected in different DIA workspaces of DeCyder software. In BVA module, Cy3 image from gel number five was chosen as master gel as it had the maximum number of spots. 26 spots were found to be differentially expressed with a criteria of average ratio more than +1.5 or less than -1.5 and a student t-test p value < 0.05. Among them, 25 spots were present in all the six gels and one spot was present in only five gels. 19 spots were found to be down regulated in the patient serum and seven were up regulated compared to the mean value of controls. List of all significant spots obtained in DeCyder are provided as Additional File 1.

**Figure 2:**
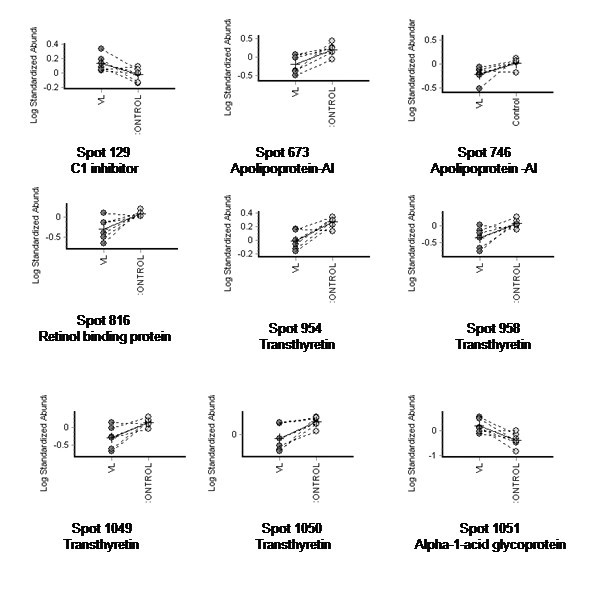
**Relative abundance of differentially expressed proteins from DeCyder**. Graphical representation of protein spots differentially expressed in sera from visceral leishmaniasis patients compared with controls (p < 0.05). Spots for which the volume ratio was ±1.5 based on DeCyder software analysis were identified by MS/MS. Data from the same gel are connected by dotted lines.

In the preparative gel stained with colloidal coomassie, all the 26 differentially expressed spots could not be visualized, probably because of low abundance. Of the fifteen spots digested and analysed by mass spectrometry, only nine spots were identified with high confidence (Figure 1). The sequence coverage of the identified proteins varied from 27 to 95%. Fold changes of protein levels of the nine identified proteins compared with controls along with the details from the Mascot search results are given in Table 2. Complete details of the Mascot search results for all spots identified are provided as Additional Files 2 and 3. The standardized log abundance of these proteins in individual gels and their comparison with control as given by the DeCyder software are illustrated in Figure 2. Transthyretin was represented by at least four and apolipoprotein-AI was represented by at least two spots. This result is not surprising as many proteins in plasma are known to exist as isoforms. In both the cases, the individual spots behaved in a similar way, being down regulated.

**Table 2:** List of differentially expressed serum proteins in visceral leishmaniasis identified by Q-TOF-MS/MS^f^

**Spot no**^**a**^.	Protein name	**Accession no.**^**b**^	**Average ratio**^**d **^**ratio ('p' (p value)**	Appaerance in gels (n = 18)	**Mascot Score**^**e**^	Peptide matches	Coverage (%)
129	C1 inhibitor	gi|73858570	+1.45 (0.026)	18	190	22	32
1051	Alpha-1- acid glycoprotein	gi|112877	+3.73 (0.009)	18	166	13	48
954	Transthyretin	gi|126030594	-1.83 (0.001)	18	449	13	95
958	Transthyretin	gi|126030594	-2.23 (0.011)	18	503	12	84
1050	Transthyretin	gi|219978	-1.84 (0.015)	18	188	5	46
1049	Transthyretin	gi|443295	-2.22 (0.011)	18	262	5	38
816	Retinol binding protein	gi|4558179	-1.99 (0.009)	18	47	6	27
673	Apolipoprotein A-I	gi|113992	-2.24 (0.013)	18	114	8	27
746	Apolipoprotein A-I	LPHUA1^c^	-1.65 (0.015)	18	314	28	63

^a ^Spot no. assigned in DeCyder analysis and corresponded to the DIGE image in Figure 1

^***b ***^Accession no. from NCBInr database, ^c ^MSDB database

^***d ***^+ indicates up-regulation and - indicates down-regulation of the protein in visceral leishmaniasis serum with reference to controls.

^e ^Mascot scores greater than 40 were considered significant

^f ^Details of all the mascot search results are provided as additional files 2 and 3.

### Western blot validation of C1 inhibitor, transthyretin and apolipoprotein-AI levels

**Figure 3:**
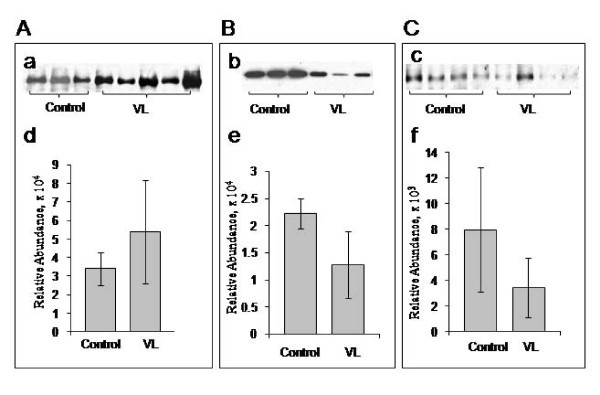
**Validation of differentially expressed proteins by western blot**. Western blot analysis of **A **C1 inhibitor, **B **transthyretin and **C **apolipoprotein A-I. The levels of **a **C1 inhibitor, **b **transthyretin and **c **apolipoprotein A-I in individual samples of each group detected by Western blot. Graphical representation of the semi quantitative analysis of Western blot results (mean ± SD of OD of bands). **d **Relative abundance of C1 inhibitor: control, 33920.4 ± 8991.7, visceral leishmaniasis, 54101.0 ± 27858.3, *p < 0.01*. **e **relative abundance of transthyretin: control, 22236.7 ± 2794.3, visceral leishmaniasis, 12804.3 ± 6128.6, *p < 0.0001*. and **f **relative abundance of apolipoprotein A-I: control, 7962.7 ± 3462.2, visceral leishmaniasis, 4846.4 ± 2319.1, *p < 0.05*

We performed western blot analyses of three proteins in a separate set of 29 undepleted serum samples to confirm the DIGE findings. Relative abundance of each band as measured by the optical density was evaluated by ImageJ software. Relative abundance of C1 inhibitor in control was 33920.4 ± 8991.7 and in visceral leishmaniasis was 54101.0 ± 27858.3 (p < 0.01) Relative abundance of transthyretin in control was 22236.7 ± 2794.3 and in visceral leishmaniasis was 12804.3 ± 6128.6 (p < 0.0001). Relative abundance of apolipoprotein A-I in control was 7962.7 ± 3462.2 and in visceral leishmaniasis was 4846.4 ± 2319.1 (p < 0.05). These results are illustrated graphically in Figure 3 and are in agreement with the DIGE analysis. The up-regulation of C1 inhibitor and the down-regulation of transthyretin and apolipoprotein A-I in visceral leishmaniasis were thus confirmed in these samples.

## Discussion

Serum is a rich source of disease-related information especially in a systemic infection like visceral leishmaniasis. Since the dynamic range of human serum proteome is large, we chose to deplete seven high abundant proteins from serum. Of all the methods employed for depletion, immunoaffinity chromatography is more effective in removing targeted proteins, with minimal carryover, high longevity, minimal nonspecific binding and high reproducibility [8,9]. However, there remains a possibility of losing some proteins by protein-protein interaction. Since the advantage conferred by depleting the high abundant proteins was deemed to be of more value in discovering low abundant proteins, we chose to deplete the serum. These seven abundant proteins make up 85-90% total protein in serum and hence, their depletion yielded a highly resolved profile of serum proteome on 2D gels enabling the analysis of low abundant proteins. According to a recent statistical study, a minimum of four biological replicates are needed to identify at least two fold difference in DIGE studies employing immunodepleted serum [10]. Assuming similar experimental conditions, our DIGE study was sufficiently powered as we used six biological replicates. Only one protein, C1-inhibitor, had an average ratio of 1.45. However, the protein's up regulation was confirmed on a larger set in western blot validation experiments.

The alteration in total protein in sera visceral leishmaniasis is a well known phenomenon [6]. However, detailed proteome analysis of the sera of this neglected tropical disease with modern technologies has not been reported so far. To our knowledge, this is the first report on the DIGE analysis of serum proteome of visceral leishmaniasis. The use of proteomics to explore the plasma proteome of related infectious diseases like human African trypanosomiasis [11], tuberculosis [12] and leprosy [13] has been reported previously. These studies reported the differential expression of many acute phase proteins in the plasma in these conditions. In this study, as expected, we found many acute phase proteins being differentially expressed. Some of the identified proteins also are important transport proteins in blood. These proteins are discussed below.

Alpha-1-acidglycoprotein is known to be elevated in systemic tissue injury, inflammation and infection. It inhibits activation, chemotaxis and their oxidative metabolism of neutrophils [14]. Alpha-1-acidglycoprotein also modulates cytokine synthesis by monocytes and macrophages [15]. Pyogenic infections of the skin and deeper tissues are common complications in patients with visceral leishmaniasis. Increased level of alpha-1-acidglycoprotein might enable these infections by inhibiting neutrophils. The deficiency of neutrophil function is reversible following successful treatment of leishmaniasis. It is interesting to note that alpha-1-acidglycoprotein has been evaluated along with serum amyloid A and C-reactive protein as potential markers for predicting response to therapy in visceral leishmaniasis [16]. These acute phase protein concentrations were significantly raised in patients who were slower to clear parasites after treatment.

C1-inhibitor is a plasma protease inhibitor and is regulator of activation of complement and kinin generating systems [17]. It inhibits both the classical and the alternate complement pathways [17,18]. C1-inhibitor also has anti inflammatory property independent of its proteolytic activity [19]. Hemolysis due to activation of alternate complement pathway is one of the major causes of anaemia in visceral leishmaniasis [20]. Therefore, it can be evaluated for its use as an additional therapeutic approach in visceral leishmaniasis to prevent complement mediated hemolysis. It is also interesting to note that, because of its anti inflammatory role, this protein and its mimics are being evaluated for its therapeutic potential in clinical trials with promising results in severe inflammatory conditions [21,22].

Transthyretin is a transporter of thyroid hormones in plasma and is a negative acute phase protein. Decreased transthyretin level during inflammation may be due to the inhibition of its production by proinflammatory cytokines during inflammation [23] or due to its increased transcapillary escape [24]. Decreased transthyretin level is described in visceral leishmaniasis in a study with a small sample size previously [25]. Transthyretin is reported to have important anti inflammatory properties as it inhibit the production of interleukin-1 by monocytes and endothelial cells [26].

Retinol binding protein transports retinol from the liver to the peripheral tissues. In plasma, retinol binding protein interacts and exists as a complex with transthyretin. This association prevents its loss through filtration in kidney [27]. Like transthyretin, retinol binding protein is also a negative acute phase protein and its production is inhibited by proinflammatory cytokines [23]. However, it may be pointed out that the serum retinol level is low in patients with leishmaniasis [28] and low retinol level contributes to low retinol binding protein level [29].

Apolipoprotein A-I is a major component of high density lipoproteins in plasma. Changes in the lipoproteins are known to occur in infantile visceral leishmaniasis, particularly, deficiency of apolipoprotein A-I and high density lipoproteins [30]. Apolipoprotein A-I is known to suppress neutrophil activation and inhibit endothelial expression of adhesion molecules [31]. It also blocks contact-mediated activation of monocytes by T lymphocytes by inhibiting the production of interleukin-1β and tumor necrosis factor-α [32]. A decrease in apolipoprotein A-I and high density lipoprotein therefore allows the uninhibited production of interleukin-1β and tumour necrosis factor-α during inflammation.

Two up regulated proteins identified in this study, alpha-1-acidglycoprotein and C1-inhibitor have anti inflammatory properties. Their elevated levels probably help decrease the tissue injury during inflammation in visceral leishmaniasis. The low level of apolipoprotein A-I leading to more proinflammatory cytokines may be seen as system defence against infection. These cytokines inhibit the production of transthyretin and retinol binding protein. Thus, there is a complex interplay among these proteins and interpreting their biological significance needs identification of more differentially expressed proteins. From the biomarker point of view, larger prospective studies incorporating appropriate controls like patients presenting with similar symptoms and employing absolute quantitative methods are suggested to establish them as biomarkers. Moreover, since these proteins are related to the inflammatory process, they will serve as good biomarkers for monitoring response to therapy. Longitudinal studies are needed in this regard to evaluate their utility as prognostic biomarkers. Since visceral leishmaniasis is endemic in resource constrained areas, simple and low cost methods need to be developed to use these results in the clinical setting. Development of simpler dipstick assays will enable such a possibility of testing these proteins in field conditions.

## Conclusions

In conclusion, DIGE based proteomic analysis showed that several proteins are differentially expressed in the sera of visceral leishmaniasis. The five proteins identified here have potential, either independently or in combination, for prognostic biomarkers. Further studies are suggested to establish their application potential.

## Competing interests

The authors declare that they have no competing interests.

## Authors' contributions

LAR wrote the main manuscript and designed and performed the most of the experiments. JK contributed to the design of the study, data collection and interpretation. GH contributed to the design of the study and revision of the manuscript draft. JCS participated in clinical sample and clinical data collection and contributed to the design of the study. AS participated in the design of the experiments, supervised the data analysis and interpretation, and participated in manuscript writing. All authors read and approved the final manuscript.

## Supplementary Material

Additional file 1 **List of differentially expressed spots in BVA**. List of differentially expressed spots in BVA, showing details for each spot (master spot number, appearance in gels, average ratio and p value). Click here for file

Additional file 2 **Detailed Mascot search results for identified proteins**. Detailed Mascot search results for the identified proteins. Mowse score for the first five hits and peptides matched are shown. Click here for file

Additional file 3 **Detailed Mascot search results for identified proteins**. Detailed Mascot search result showing the protein view. Score and sequence coverage for the identified protein and list of all the peptides matched is shown. Click here for file
