# Supplementary material for: Two-dimensional difference gel electrophoresis (DIGE) analysis of sera from visceral leishmaniasis patients
Source: Clin Proteomics. 2011 May 31;8(1):4. doi: 10.1186/1559-0275-8-4 (PMC3167202; doi:10.1186/1559-0275-8-4)
Supplement: Additional file 2 — Detailed Mascot search results for identified proteins. Detailed Mascot search results for the identified proteins. Mowse score for the first five hits and peptides matched are shown. [file 1559-0275-8-4-S2.DOC]

**Mascot Search Results**

**Spot 746**

**User : A.Srinivasan**

**Email : srini@aiims.ac.in**

**Search title : D:\PE Sciex Data\Projects\Default\Data\Data lo kala spot 1lo kala spot 1.wiff (sample number 1)**

**MS data file : C:\Temp\mas4D.tmp**

**Database : MSDB 20060831 (3239079 sequences; 1079594700 residues)**

**Taxonomy : Homo sapiens (human) (148148 sequences)**

**Timestamp : 4 Apr 2008 at 11:16:29 GMT**

| **Protein hits    :** | **[LPHUA1](http://www.matrixscience.com/cgi/master_results.pl?file=../data/20080404/FtgoSrETt.dat#Hit1)** | apolipoprotein A-I precursor [validated] - human |
| --- | --- | --- |
|  | **[CAC39960](http://www.matrixscience.com/cgi/master_results.pl?file=../data/20080404/FtgoSrETt.dat#Hit2)** | Sequence 7 from Patent EP1101772.- Homo sapiens (Human). |
|  | **[Q8WWZ8_HUMAN](http://www.matrixscience.com/cgi/master_results.pl?file=../data/20080404/FtgoSrETt.dat#Hit3)** | LZP (PPFL826) (CDNA FLJ39116 fis, clone NTONG2006301, highly similar to Mus musculus EF-9 mRNA).- Homo sapiens (Human). |
|  | **[Q86TH5_HUMAN](http://www.matrixscience.com/cgi/master_results.pl?file=../data/20080404/FtgoSrETt.dat#Hit4)** | ARFGEF2 protein (Fragment).- Homo sapiens (Human). |
|  | **[PC2205](http://www.matrixscience.com/cgi/master_results.pl?file=../data/20080404/FtgoSrETt.dat#Hit5)** | interferon-alpha LCA-2b binding subtype - human (fragment) |

**Probability Based Mowse Score**

Ions score is -10*Log(P), where P is the probability that the observed match is a random event.
Individual ions scores > 40 indicate identity or extensive homology (p<0.05).
Protein scores are derived from ions scores as a non-probabilistic basis for ranking protein hits.


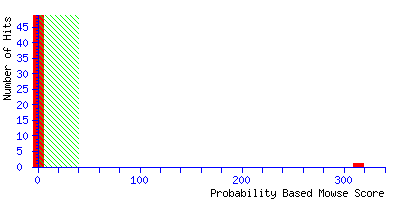


Top of Form

Peptide Summary Report

|  |  |  | [Help](http://www.matrixscience.com/help/results_help.html#FORMAT) |
| --- | --- | --- | --- |
|  | Significance threshold p< | Max. number of hits |  |
|  | Standard scoring  MudPIT scoring | Ions score or expect cut-off | Show sub-sets |
|  | Show pop-ups  Suppress pop-ups | Sort unassigned | Require bold red |

Bottom of Form

Top of Form

**Error tolerant**

| **1.** | [LPHUA1](http://www.matrixscience.com/cgi/protein_view.pl?file=../data/20080404/FtgoSrETt.dat&hit=LPHUA1&px=1&ave_thresh=40&_sigthreshold=0.05&_server_mudpit_switch=0.001)    **Mass:** 30759    **Score:** 314    **Queries matched:** 28   **emPAI:** 4.69 |
| --- | --- |
|  | apolipoprotein A-I precursor [validated] - human |

|  | Check to include this hit in error tolerant search |
| --- | --- |
|  |  |

|  | **Query** | **Observed** | **Mr(expt)** | **Mr(calc)** | **Delta** | **Miss** | **Score** | **Expect** | **Rank** | **Peptide** |
| --- | --- | --- | --- | --- | --- | --- | --- | --- | --- | --- |
|  | [11](http://www.matrixscience.com/cgi/peptide_view.pl?file=../data/20080404/FtgoSrETt.dat&query=11&hit=1&index=LPHUA1&px=1&section=5&ave_thresh=40) | **416.2180** | **830.4215** | **830.4286** | **-0.0072** | **0** | **23** | **5.3** | **1** | **R.LAEYHAK.A** |
|  | [21](http://www.matrixscience.com/cgi/peptide_view.pl?file=../data/20080404/FtgoSrETt.dat&query=21&hit=1&index=LPHUA1&px=1&section=5&ave_thresh=40) | **435.2465** | **868.4784** | **868.5130** | **-0.0347** | **1** | **15** | **19** | **1** | **R.QKVEPLR.A** |
|  | [22](http://www.matrixscience.com/cgi/peptide_view.pl?file=../data/20080404/FtgoSrETt.dat&query=22&hit=1&index=LPHUA1&px=1&section=5&ave_thresh=40) | **437.2162** | **872.4179** | **872.4352** | **-0.0173** | **0** | **48** | **0.015** | **1** | **R.AELQEGAR.Q** |
|  | [25](http://www.matrixscience.com/cgi/peptide_view.pl?file=../data/20080404/FtgoSrETt.dat&query=25&hit=1&index=LPHUA1&px=1&section=5&ave_thresh=40) | **448.2365** | **894.4585** | **895.4763** | **-1.0178** | **0** | **(19)** | **9.8** | **1** | **K.LHELQEK.L** |
|  | [26](http://www.matrixscience.com/cgi/peptide_view.pl?file=../data/20080404/FtgoSrETt.dat&query=26&hit=1&index=LPHUA1&px=1&section=5&ave_thresh=40) | **448.7388** | **895.4631** | **895.4763** | **-0.0132** | **0** | **27** | **1.3** | **1** | **K.LHELQEK.L** |
|  | [44](http://www.matrixscience.com/cgi/peptide_view.pl?file=../data/20080404/FtgoSrETt.dat&query=44&hit=1&index=LPHUA1&px=1&section=5&ave_thresh=40) | **506.7874** | **1011.5602** | **1011.5713** | **-0.0110** | **0** | **34** | **0.26** | **1** | **K.AKPALEDLR.Q** |
|  | [47](http://www.matrixscience.com/cgi/peptide_view.pl?file=../data/20080404/FtgoSrETt.dat&query=47&hit=1&index=LPHUA1&px=1&section=5&ave_thresh=40) | **516.2606** | **1030.5067** | **1030.5117** | **-0.0050** | **0** | **50** | **0.0073** | **1** | **K.LSPLGEEMR.D** |
|  | [53](http://www.matrixscience.com/cgi/peptide_view.pl?file=../data/20080404/FtgoSrETt.dat&query=53&hit=1&index=LPHUA1&px=1&section=5&ave_thresh=40) | **524.7558** | **1047.4971** | **1046.5066** | **0.9905** | **0** | **(33)** | **0.34** | **1** | **K.LSPLGEEMR.D + Oxidation (M)** |
|  | [69](http://www.matrixscience.com/cgi/peptide_view.pl?file=../data/20080404/FtgoSrETt.dat&query=69&hit=1&index=LPHUA1&px=1&section=5&ave_thresh=40) | **607.8360** | **1213.6574** | **1214.6143** | **-0.9568** | **0** | **40** | **0.063** | **1** | **K.ATEHLSTLSEK.A** |
|  | [70](http://www.matrixscience.com/cgi/peptide_view.pl?file=../data/20080404/FtgoSrETt.dat&query=70&hit=1&index=LPHUA1&px=1&section=5&ave_thresh=40) | **405.8749** | **1214.6029** | **1214.6143** | **-0.0114** | **0** | **(36)** | **0.17** | **1** | **K.ATEHLSTLSEK.A** |
|  | [73](http://www.matrixscience.com/cgi/peptide_view.pl?file=../data/20080404/FtgoSrETt.dat&query=73&hit=1&index=LPHUA1&px=1&section=5&ave_thresh=40) | **615.8476** | **1229.6807** | **1229.7020** | **-0.0213** | **0** | **50** | **0.0066** | **1** | **R.QGLLPVLESFK.V** |
|  | [75](http://www.matrixscience.com/cgi/peptide_view.pl?file=../data/20080404/FtgoSrETt.dat&query=75&hit=1&index=LPHUA1&px=1&section=5&ave_thresh=40) | **618.3441** | **1234.6736** | **1234.6809** | **-0.0073** | **0** | **47** | **0.015** | **1** | **K.DLATVYVDVLK.D** |
|  | [85](http://www.matrixscience.com/cgi/peptide_view.pl?file=../data/20080404/FtgoSrETt.dat&query=85&hit=1&index=LPHUA1&px=1&section=5&ave_thresh=40) | **642.2958** | **1282.5770** | **1282.5652** | **0.0118** | **0** | **34** | **0.22** | **1** | **K.WQEEMELYR.Q** |
|  | [91](http://www.matrixscience.com/cgi/peptide_view.pl?file=../data/20080404/FtgoSrETt.dat&query=91&hit=1&index=LPHUA1&px=1&section=5&ave_thresh=40) | **650.2916** | **1298.5686** | **1298.5601** | **0.0085** | **0** | **(7)** | **1.1e+02** | **1** | **K.WQEEMELYR.Q + Oxidation (M)** |
|  | [92](http://www.matrixscience.com/cgi/peptide_view.pl?file=../data/20080404/FtgoSrETt.dat&query=92&hit=1&index=LPHUA1&px=1&section=5&ave_thresh=40) | **434.2027** | **1299.5863** | **1300.6411** | **-1.0549** | **0** | **37** | **0.12** | **1** | **R.THLAPYSDELR.Q** |
|  | [93](http://www.matrixscience.com/cgi/peptide_view.pl?file=../data/20080404/FtgoSrETt.dat&query=93&hit=1&index=LPHUA1&px=1&section=5&ave_thresh=40) | **434.5505** | **1300.6296** | **1300.6411** | **-0.0115** | **0** | **(34)** | **0.22** | **1** | **R.THLAPYSDELR.Q** |
|  | [109](http://www.matrixscience.com/cgi/peptide_view.pl?file=../data/20080404/FtgoSrETt.dat&query=109&hit=1&index=LPHUA1&px=1&section=5&ave_thresh=40) | **460.9002** | **1379.6788** | **1379.7085** | **-0.0298** | **1** | **(11)** | **50** | **1** | **K.VQPYLDDFQKK.W** |
|  | [110](http://www.matrixscience.com/cgi/peptide_view.pl?file=../data/20080404/FtgoSrETt.dat&query=110&hit=1&index=LPHUA1&px=1&section=5&ave_thresh=40) | **461.2444** | **1380.7114** | **1379.7085** | **1.0029** | **1** | **14** | **23** | **1** | **K.VQPYLDDFQKK.W** |
|  | [111](http://www.matrixscience.com/cgi/peptide_view.pl?file=../data/20080404/FtgoSrETt.dat&query=111&hit=1&index=LPHUA1&px=1&section=5&ave_thresh=40) | **693.8542** | **1385.6939** | **1385.7078** | **-0.0139** | **0** | **68** | **9.5e-05** | **1** | **K.VSFLSALEEYTK.K** |
|  | [113](http://www.matrixscience.com/cgi/peptide_view.pl?file=../data/20080404/FtgoSrETt.dat&query=113&hit=1&index=LPHUA1&px=1&section=5&ave_thresh=40) | **467.2090** | **1398.6052** | **1399.6620** | **-1.0567** | **0** | **(15)** | **18** | **1** | **R.DYVSQFEGSALGK.Q** |
|  | [114](http://www.matrixscience.com/cgi/peptide_view.pl?file=../data/20080404/FtgoSrETt.dat&query=114&hit=1&index=LPHUA1&px=1&section=5&ave_thresh=40) | **700.8403** | **1399.6661** | **1399.6620** | **0.0042** | **0** | **47** | **0.011** | **1** | **R.DYVSQFEGSALGK.Q** |
|  | [117](http://www.matrixscience.com/cgi/peptide_view.pl?file=../data/20080404/FtgoSrETt.dat&query=117&hit=1&index=LPHUA1&px=1&section=5&ave_thresh=40) | **471.2562** | **1410.7469** | **1410.6601** | **0.0867** | **1** | **19** | **7.1** | **1** | **K.KWQEEMELYR.Q** |
|  | [119](http://www.matrixscience.com/cgi/peptide_view.pl?file=../data/20080404/FtgoSrETt.dat&query=119&hit=5&index=LPHUA1&px=1&section=5&ave_thresh=40) | **476.5541** | **1426.6406** | **1426.6551** | **-0.0145** | **1** | **(8)** | **84** | **5** | **K.KWQEEMELYR.Q + Oxidation (M)** |
|  | [123](http://www.matrixscience.com/cgi/peptide_view.pl?file=../data/20080404/FtgoSrETt.dat&query=123&hit=1&index=LPHUA1&px=1&section=5&ave_thresh=40) | **488.2864** | **1461.8374** | **1461.8443** | **-0.0068** | **1** | **36** | **0.13** | **1** | **R.VKDLATVYVDVLK.D** |
|  | [133](http://www.matrixscience.com/cgi/peptide_view.pl?file=../data/20080404/FtgoSrETt.dat&query=133&hit=1&index=LPHUA1&px=1&section=5&ave_thresh=40) | **806.8888** | **1611.7630** | **1611.7781** | **-0.0150** | **0** | **85** | **1.7e-06** | **1** | **K.LLDNWDSVTSTFSK.L** |
|  | [143](http://www.matrixscience.com/cgi/peptide_view.pl?file=../data/20080404/FtgoSrETt.dat&query=143&hit=4&index=LPHUA1&px=1&section=5&ave_thresh=40) | **645.3143** | **1932.9211** | **1931.9265** | **0.9946** | **0** | **1** | **3.7e+02** | **4** | **R.EQLGPVTQEFWDNLEK.E** |
|  | [152](http://www.matrixscience.com/cgi/peptide_view.pl?file=../data/20080404/FtgoSrETt.dat&query=152&hit=1&index=LPHUA1&px=1&section=5&ave_thresh=40) | **734.6992** | **2201.0757** | **2201.1117** | **-0.0359** | **1** | **44** | **0.014** | **1** | **K.LREQLGPVTQEFWDNLEK.E** |
|  | [153](http://www.matrixscience.com/cgi/peptide_view.pl?file=../data/20080404/FtgoSrETt.dat&query=153&hit=1&index=LPHUA1&px=1&section=5&ave_thresh=40) | **735.0296** | **2202.0668** | **2201.1117** | **0.9552** | **1** | **(32)** | **0.26** | **1** | **K.LREQLGPVTQEFWDNLEK.E** |

|  | |
| --- | --- |
|  | **Proteins matching the same set of peptides:** |

|  | [CAA00975](http://www.matrixscience.com/cgi/protein_view.pl?file=../data/20080404/FtgoSrETt.dat&hit=CAA00975&px=1&ave_thresh=40&_sigthreshold=0.05&_server_mudpit_switch=0.001)    **Mass:** 28061    **Score:** 314    **Queries matched:** 28 |
| --- | --- |
|  | APOA1 PROTEIN (FRAGMENT).- Homo sapiens (Human). |

|  | [AAA51747](http://www.matrixscience.com/cgi/protein_view.pl?file=../data/20080404/FtgoSrETt.dat&hit=AAA51747&px=1&ave_thresh=40&_sigthreshold=0.05&_server_mudpit_switch=0.001)    **Mass:** 28944    **Score:** 314    **Queries matched:** 28 |
| --- | --- |
|  | HUMAPOAIC NID: - Homo sapiens |

Bottom of Form

**Mascot Search Results**

**Spot 114**

**User : A.Srinivasan**

**Email : srini@aiims.ac.in**

**Search title : D:\PE Sciex Data\Projects\Default\Data\Data lo kala spot 2alo kala spot 2a.wiff (sample number 1)**

**MS data file : C:\Temp\mas15.tmp**

**Database : MSDB 20060831 (3239079 sequences; 1079594700 residues)**

**Taxonomy : Homo sapiens (human) (148148 sequences)**

**Timestamp : 17 Apr 2008 at 13:10:07 GMT**

| **Protein hits    :** | **[LPHUA1](http://www.matrixscience.com/cgi/master_results.pl?file=../data/20080417/FtgmriYOE.dat#Hit1)** | apolipoprotein A-I precursor [validated] - human |
| --- | --- | --- |
|  | **[Q4EW67_ADE09](http://www.matrixscience.com/cgi/master_results.pl?file=../data/20080417/FtgmriYOE.dat#Hit2)** | Hexon protein (Fragment).- Human adenovirus 9 (HAdV-9). |
|  | **[Q3KQZ0_HUMAN](http://www.matrixscience.com/cgi/master_results.pl?file=../data/20080417/FtgmriYOE.dat#Hit3)** | NUP214 protein.- Homo sapiens (Human). |
|  | **[K1688_HUMAN](http://www.matrixscience.com/cgi/master_results.pl?file=../data/20080417/FtgmriYOE.dat#Hit4)** | Protein KIAA1688.- Homo sapiens (Human). |
|  | **[BAA20829](http://www.matrixscience.com/cgi/master_results.pl?file=../data/20080417/FtgmriYOE.dat#Hit5)** | AB002372 NID: - Homo sapiens |

**Probability Based Mowse Score**

Ions score is -10*Log(P), where P is the probability that the observed match is a random event.
Individual ions scores > 40 indicate identity or extensive homology (p<0.05).
Protein scores are derived from ions scores as a non-probabilistic basis for ranking protein hits.


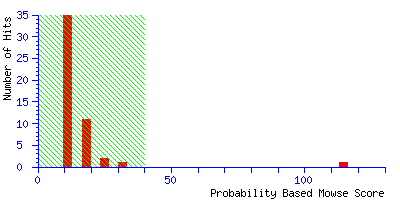


Top of Form

Peptide Summary Report

|  |  |  | [Help](http://www.matrixscience.com/help/results_help.html#FORMAT) |
| --- | --- | --- | --- |
|  | Significance threshold p< | Max. number of hits |  |
|  | Standard scoring  MudPIT scoring | Ions score or expect cut-off | Show sub-sets |
|  | Show pop-ups  Suppress pop-ups | Sort unassigned | Require bold red |

Bottom of Form

Top of Form

**Error tolerant**

| **1.** | [LPHUA1](http://www.matrixscience.com/cgi/protein_view.pl?file=../data/20080417/FtgmriYOE.dat&hit=LPHUA1&px=1&ave_thresh=40&_sigthreshold=0.05&_server_mudpit_switch=0.001)    **Mass:** 30759    **Score:** 114    **Queries matched:** 8 |
| --- | --- |
|  | apolipoprotein A-I precursor [validated] - human |

|  | Check to include this hit in error tolerant search |
| --- | --- |
|  |  |

|  | **Query** | **Observed** | **Mr(expt)** | **Mr(calc)** | **Delta** | **Miss** | **Score** | **Expect** | **Rank** | **Peptide** |
| --- | --- | --- | --- | --- | --- | --- | --- | --- | --- | --- |
|  | [9](http://www.matrixscience.com/cgi/peptide_view.pl?file=../data/20080417/FtgmriYOE.dat&query=9&hit=2&index=LPHUA1&px=1&section=5&ave_thresh=40) | **437.2117** | **872.4088** | **872.4352** | **-0.0263** | **0** | **16** | **21** | **2** | **R.AELQEGAR.Q** |
|  | [17](http://www.matrixscience.com/cgi/peptide_view.pl?file=../data/20080417/FtgmriYOE.dat&query=17&hit=1&index=LPHUA1&px=1&section=5&ave_thresh=40) | **516.2865** | **1030.5585** | **1030.5117** | **0.0468** | **0** | **(15)** | **25** | **1** | **K.LSPLGEEMR.D** |
|  | [20](http://www.matrixscience.com/cgi/peptide_view.pl?file=../data/20080417/FtgmriYOE.dat&query=20&hit=1&index=LPHUA1&px=1&section=5&ave_thresh=40) | **524.3222** | **1046.6298** | **1046.5066** | **0.1232** | **0** | **15** | **23** | **1** | **K.LSPLGEEMR.D + Oxidation (M)** |
|  | [25](http://www.matrixscience.com/cgi/peptide_view.pl?file=../data/20080417/FtgmriYOE.dat&query=25&hit=1&index=LPHUA1&px=1&section=5&ave_thresh=40) | **626.8052** | **1251.5958** | **1251.6136** | **-0.0177** | **0** | **22** | **3.7** | **1** | **K.VQPYLDDFQK.K** |
|  | [27](http://www.matrixscience.com/cgi/peptide_view.pl?file=../data/20080417/FtgmriYOE.dat&query=27&hit=2&index=LPHUA1&px=1&section=5&ave_thresh=40) | **642.3076** | **1282.6007** | **1282.5652** | **0.0355** | **0** | **8** | **1.1e+02** | **2** | **K.WQEEMELYR.Q** |
|  | [29](http://www.matrixscience.com/cgi/peptide_view.pl?file=../data/20080417/FtgmriYOE.dat&query=29&hit=1&index=LPHUA1&px=1&section=5&ave_thresh=40) | **434.8881** | **1301.6425** | **1300.6411** | **1.0014** | **0** | **24** | **2.5** | **1** | **R.THLAPYSDELR.Q** |
|  | [36](http://www.matrixscience.com/cgi/peptide_view.pl?file=../data/20080417/FtgmriYOE.dat&query=36&hit=2&index=LPHUA1&px=1&section=5&ave_thresh=40) | **701.3393** | **1400.6641** | **1399.6620** | **1.0022** | **0** | **12** | **35** | **2** | **R.DYVSQFEGSALGK.Q** |
|  | [38](http://www.matrixscience.com/cgi/peptide_view.pl?file=../data/20080417/FtgmriYOE.dat&query=38&hit=1&index=LPHUA1&px=1&section=5&ave_thresh=40) | **807.3853** | **1612.7560** | **1611.7781** | **0.9780** | **0** | **18** | **7.8** | **1** | **K.LLDNWDSVTSTFSK.L** |

|  | |
| --- | --- |
|  | **Proteins matching the same set of peptides:** |

|  | [CAA00975](http://www.matrixscience.com/cgi/protein_view.pl?file=../data/20080417/FtgmriYOE.dat&hit=CAA00975&px=1&ave_thresh=40&_sigthreshold=0.05&_server_mudpit_switch=0.001)    **Mass:** 28061    **Score:** 114    **Queries matched:** 8 |
| --- | --- |
|  | APOA1 PROTEIN (FRAGMENT).- Homo sapiens (Human). |

|  | [AAA35545](http://www.matrixscience.com/cgi/protein_view.pl?file=../data/20080417/FtgmriYOE.dat&hit=AAA35545&px=1&ave_thresh=40&_sigthreshold=0.05&_server_mudpit_switch=0.001)    **Mass:** 30745    **Score:** 114    **Queries matched:** 8 |
| --- | --- |
|  | HUMAPOAIP NID: - Homo sapiens |

|  | [AAA51747](http://www.matrixscience.com/cgi/protein_view.pl?file=../data/20080417/FtgmriYOE.dat&hit=AAA51747&px=1&ave_thresh=40&_sigthreshold=0.05&_server_mudpit_switch=0.001)    **Mass:** 28944    **Score:** 114    **Queries matched:** 8 |
| --- | --- |
|  | HUMAPOAIC NID: - Homo sapiens |

Bottom of Form

**Mascot Search Results**

**Spot 816**

**User : A.Srinivasan**

**Email : srini@aiims.ac.in**

**Search title : D:\PE Sciex Data\Projects\Default\Data\Data lo kala spot 3lo kala spot 3.wiff (sample number 1)**

**MS data file : C:\Temp\masA.tmp**

**Database : MSDB 20060831 (3239079 sequences; 1079594700 residues)**

**Taxonomy : Homo sapiens (human) (148148 sequences)**

**Timestamp : 11 Apr 2008 at 09:52:34 GMT**

| **Protein hits    :** | **[1QABE](http://www.matrixscience.com/cgi/master_results.pl?file=../data/20080411/FtgoCnumR.dat#Hit1)** | retinol binding protein, chain E - human |
| --- | --- | --- |
|  | **[Q5JVP6_HUMAN](http://www.matrixscience.com/cgi/master_results.pl?file=../data/20080411/FtgoCnumR.dat#Hit2)** | Rac/Cdc42 guanine nucleotide exchange factor (GEF) 6.- Homo sapiens (Human). |
|  | **[CAD38234](http://www.matrixscience.com/cgi/master_results.pl?file=../data/20080411/FtgoCnumR.dat#Hit3)** | Sequence 1 from Patent WO0206331.- Homo sapiens (Human). |
|  | **[AAD33397](http://www.matrixscience.com/cgi/master_results.pl?file=../data/20080411/FtgoCnumR.dat#Hit4)** | AF128881 NID: - Homo sapiens |
|  | **[Q86TH5_HUMAN](http://www.matrixscience.com/cgi/master_results.pl?file=../data/20080411/FtgoCnumR.dat#Hit5)** | ARFGEF2 protein (Fragment).- Homo sapiens (Human). |

**Probability Based Mowse Score**

Ions score is -10*Log(P), where P is the probability that the observed match is a random event.
Individual ions scores > 40 indicate identity or extensive homology (p<0.05).
Protein scores are derived from ions scores as a non-probabilistic basis for ranking protein hits.


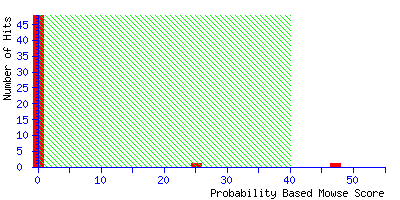


Top of Form

Peptide Summary Report

|  |  |  | [Help](http://www.matrixscience.com/help/results_help.html#FORMAT) |
| --- | --- | --- | --- |
|  | Significance threshold p< | Max. number of hits |  |
|  | Standard scoring  MudPIT scoring | Ions score or expect cut-off | Show sub-sets |
|  | Show pop-ups  Suppress pop-ups | Sort unassigned | Require bold red |

Bottom of Form

Top of Form

**Error tolerant**

| **1.** | [1QABE](http://www.matrixscience.com/cgi/protein_view.pl?file=../data/20080411/FtgoCnumR.dat&hit=1QABE&px=1&ave_thresh=40&_sigthreshold=0.05&_server_mudpit_switch=0.001)    **Mass:** 20745    **Score:** 47     **Queries matched:** 6   **emPAI:** 0.66 |
| --- | --- |
|  | retinol binding protein, chain E - human |

|  | Check to include this hit in error tolerant search |
| --- | --- |
|  |  |

|  | **Query** | **Observed** | **Mr(expt)** | **Mr(calc)** | **Delta** | **Miss** | **Score** | **Expect** | **Rank** | **Peptide** |
| --- | --- | --- | --- | --- | --- | --- | --- | --- | --- | --- |
|  | [140](http://www.matrixscience.com/cgi/peptide_view.pl?file=../data/20080411/FtgoCnumR.dat&query=140&hit=1&index=1QABE&px=1&section=5&ave_thresh=40) | **581.2623** | **1160.5100** | **1160.5325** | **-0.0225** | **0** | **23** | **3.7** | **1** | **R.FSGTWYAMAK.K** |
|  | [145](http://www.matrixscience.com/cgi/peptide_view.pl?file=../data/20080411/FtgoCnumR.dat&query=145&hit=1&index=1QABE&px=1&section=5&ave_thresh=40) | **599.8079** | **1197.6013** | **1197.6182** | **-0.0169** | **0** | **31** | **0.46** | **1** | **K.YWGVASFLQK.G** |
|  | [168](http://www.matrixscience.com/cgi/peptide_view.pl?file=../data/20080411/FtgoCnumR.dat&query=168&hit=1&index=1QABE&px=1&section=5&ave_thresh=40) | **434.8925** | **1301.6558** | **1302.6139** | **-0.9581** | **0** | **36** | **0.17** | **1** | **R.LIVHNGYCDGR.S** |
|  | [169](http://www.matrixscience.com/cgi/peptide_view.pl?file=../data/20080411/FtgoCnumR.dat&query=169&hit=1&index=1QABE&px=1&section=5&ave_thresh=40) | **435.2249** | **1302.6530** | **1302.6139** | **0.0391** | **0** | **(29)** | **0.9** | **1** | **R.LIVHNGYCDGR.S** |
|  | [171](http://www.matrixscience.com/cgi/peptide_view.pl?file=../data/20080411/FtgoCnumR.dat&query=171&hit=1&index=1QABE&px=1&section=5&ave_thresh=40) | **435.5379** | **1303.5919** | **1302.6139** | **0.9780** | **0** | **(18)** | **11** | **1** | **R.LIVHNGYCDGR.S** |
|  | [229](http://www.matrixscience.com/cgi/peptide_view.pl?file=../data/20080411/FtgoCnumR.dat&query=229&hit=1&index=1QABE&px=1&section=5&ave_thresh=40) | **688.9734** | **2063.8985** | **2063.9623** | **-0.0638** | **0** | **19** | **4.5** | **1** | **R.LLNLDGTCADSYSFVFSR.D** |

|  | |
| --- | --- |
|  | **Proteins matching the same set of peptides:** |

|  | [VAHU](http://www.matrixscience.com/cgi/protein_view.pl?file=../data/20080411/FtgoCnumR.dat&hit=VAHU&px=1&ave_thresh=40&_sigthreshold=0.05&_server_mudpit_switch=0.001)    **Mass:** 23195    **Score:** 47     **Queries matched:** 6 |
| --- | --- |
|  | plasma retinol-binding protein precursor [validated] - human |

|  | [Q5VY30_HUMAN](http://www.matrixscience.com/cgi/protein_view.pl?file=../data/20080411/FtgoCnumR.dat&hit=Q5VY30_HUMAN&px=1&ave_thresh=40&_sigthreshold=0.05&_server_mudpit_switch=0.001)    **Mass:** 23271    **Score:** 47     **Queries matched:** 6 |
| --- | --- |
|  | Retinol binding protein 4, plasma.- Homo sapiens (Human). |

|  | [AAH20633](http://www.matrixscience.com/cgi/protein_view.pl?file=../data/20080411/FtgoCnumR.dat&hit=AAH20633&px=1&ave_thresh=40&_sigthreshold=0.05&_server_mudpit_switch=0.001)    **Mass:** 23371    **Score:** 47     **Queries matched:** 6 |
| --- | --- |
|  | BC020633 NID: - Homo sapiens |

|  | [CAH72328](http://www.matrixscience.com/cgi/protein_view.pl?file=../data/20080411/FtgoCnumR.dat&hit=CAH72328&px=1&ave_thresh=40&_sigthreshold=0.05&_server_mudpit_switch=0.001)    **Mass:** 23337    **Score:** 47     **Queries matched:** 6 |
| --- | --- |
|  | AL356214 NID: - Homo sapiens |

Bottom of Form

**Mascot Search Results**

**Spot 1051**

**User : A.Srinivasan**

**Email : srini@aiims.ac.in**

**Search title : D:\PE Sciex Data\Projects\Default\Data\Data lo kala spot 9lo kala spot 9.wiff (sample number 1)**

**MS data file : C:\Temp\mas57.tmp**

**Database : MSDB 20060831 (3239079 sequences; 1079594700 residues)**

**Taxonomy : Homo sapiens (human) (148148 sequences)**

**Timestamp : 7 Apr 2008 at 06:57:42 GMT**

| **Protein hits    :** | **[OMHU1](http://www.matrixscience.com/cgi/master_results.pl?file=../data/20080407/FtgoinTne.dat#Hit1)** | alpha-1-acid glycoprotein 1 precursor [validated] - human |
| --- | --- | --- |
|  | **[OMHU2](http://www.matrixscience.com/cgi/master_results.pl?file=../data/20080407/FtgoinTne.dat#Hit2)** | alpha-1-acid glycoprotein 2 precursor - human |
|  | **[CAE98367](http://www.matrixscience.com/cgi/master_results.pl?file=../data/20080407/FtgoinTne.dat#Hit3)** | AX884253 NID: - Homo sapiens |
|  | **[BAA34292](http://www.matrixscience.com/cgi/master_results.pl?file=../data/20080407/FtgoinTne.dat#Hit4)** | ALPHA1-ACID GLYCOPROTEIN (FRAGMENT).- Homo sapiens (Human). |
|  | **[Q9UL10_HUMAN](http://www.matrixscience.com/cgi/master_results.pl?file=../data/20080407/FtgoinTne.dat#Hit5)** | Hypothetical protein SARDH.- Homo sapiens (Human). |

**Probability Based Mowse Score**

Ions score is -10*Log(P), where P is the probability that the observed match is a random event.
Individual ions scores > 40 indicate identity or extensive homology (p<0.05).
Protein scores are derived from ions scores as a non-probabilistic basis for ranking protein hits.


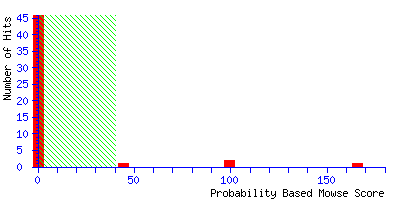


Top of Form

Peptide Summary Report

|  |  |  | [Help](http://www.matrixscience.com/help/results_help.html#FORMAT) |
| --- | --- | --- | --- |
|  | Significance threshold p< | Max. number of hits |  |
|  | Standard scoring  MudPIT scoring | Ions score or expect cut-off | Show sub-sets |
|  | Show pop-ups  Suppress pop-ups | Sort unassigned | Require bold red |

Bottom of Form

Top of Form

**Error tolerant**

| **1.** | [OMHU1](http://www.matrixscience.com/cgi/protein_view.pl?file=../data/20080407/FtgoinTne.dat&hit=OMHU1&px=1&ave_thresh=40&_sigthreshold=0.05&_server_mudpit_switch=0.001)    **Mass:** 23725    **Score:** 166    **Queries matched:** 13   **emPAI:** 1.84 |
| --- | --- |
|  | alpha-1-acid glycoprotein 1 precursor [validated] - human |

|  | Check to include this hit in error tolerant search |
| --- | --- |
|  |  |

|  | **Query** | **Observed** | **Mr(expt)** | **Mr(calc)** | **Delta** | **Miss** | **Score** | **Expect** | **Rank** | **Peptide** |
| --- | --- | --- | --- | --- | --- | --- | --- | --- | --- | --- |
|  | [19](http://www.matrixscience.com/cgi/peptide_view.pl?file=../data/20080407/FtgoinTne.dat&query=19&hit=8&index=OMHU1&px=1&section=5&ave_thresh=40) | **404.2000** | **806.3854** | **805.3454** | **1.0401** | **1** | **11** | **69** | **8** | **R.KQEEGES.-** |
|  | [49](http://www.matrixscience.com/cgi/peptide_view.pl?file=../data/20080407/FtgoinTne.dat&query=49&hit=1&index=OMHU1&px=1&section=5&ave_thresh=40) | **497.7657** | **993.5168** | **993.5131** | **0.0037** | **0** | **(54)** | **0.003** | **1** | **K.TEDTIFLR.E** |
|  | [50](http://www.matrixscience.com/cgi/peptide_view.pl?file=../data/20080407/FtgoinTne.dat&query=50&hit=1&index=OMHU1&px=1&section=5&ave_thresh=40) | **497.9000** | **993.7854** | **993.5131** | **0.2723** | **0** | **54** | **0.0017** | **1** | **K.TEDTIFLR.E** |
|  | [54](http://www.matrixscience.com/cgi/peptide_view.pl?file=../data/20080407/FtgoinTne.dat&query=54&hit=1&index=OMHU1&px=1&section=5&ave_thresh=40) | **509.2574** | **1016.5002** | **1017.4801** | **-0.9798** | **1** | **29** | **0.95** | **1** | **K.DKCEPLEK.Q** |
|  | [55](http://www.matrixscience.com/cgi/peptide_view.pl?file=../data/20080407/FtgoinTne.dat&query=55&hit=1&index=OMHU1&px=1&section=5&ave_thresh=40) | **509.7441** | **1017.4736** | **1017.4801** | **-0.0064** | **1** | **(21)** | **6.2** | **1** | **K.DKCEPLEK.Q** |
|  | [71](http://www.matrixscience.com/cgi/peptide_view.pl?file=../data/20080407/FtgoinTne.dat&query=71&hit=1&index=OMHU1&px=1&section=5&ave_thresh=40) | **556.7613** | **1111.5081** | **1111.5186** | **-0.0105** | **0** | **42** | **0.039** | **1** | **K.SDVVYTDWK.K** |
|  | [80](http://www.matrixscience.com/cgi/peptide_view.pl?file=../data/20080407/FtgoinTne.dat&query=80&hit=1&index=OMHU1&px=1&section=5&ave_thresh=40) | **580.7968** | **1159.5791** | **1159.5815** | **-0.0023** | **0** | **47** | **0.015** | **1** | **K.WFYIASAFR.N** |
|  | [120](http://www.matrixscience.com/cgi/peptide_view.pl?file=../data/20080407/FtgoinTne.dat&query=120&hit=1&index=OMHU1&px=1&section=5&ave_thresh=40) | **723.3700** | **1444.7254** | **1444.6544** | **0.0710** | **0** | **42** | **0.035** | **1** | **K.TYMLAFDVNDEK.N** |
|  | [122](http://www.matrixscience.com/cgi/peptide_view.pl?file=../data/20080407/FtgoinTne.dat&query=122&hit=1&index=OMHU1&px=1&section=5&ave_thresh=40) | **731.3468** | **1460.6790** | **1460.6493** | **0.0297** | **0** | **(10)** | **53** | **1** | **K.TYMLAFDVNDEK.N + Oxidation (M)** |
|  | [134](http://www.matrixscience.com/cgi/peptide_view.pl?file=../data/20080407/FtgoinTne.dat&query=134&hit=1&index=OMHU1&px=1&section=5&ave_thresh=40) | **570.2828** | **1707.8265** | **1707.8468** | **-0.0203** | **0** | **26** | **1.2** | **1** | **K.NWGLSVYADKPETTK.E** |
|  | [137](http://www.matrixscience.com/cgi/peptide_view.pl?file=../data/20080407/FtgoinTne.dat&query=137&hit=1&index=OMHU1&px=1&section=5&ave_thresh=40) | **872.3926** | **1742.7707** | **1741.7981** | **0.9725** | **0** | **68** | **8.3e-05** | **1** | **K.EQLGEFYEALDCLR.I** |
|  | [139](http://www.matrixscience.com/cgi/peptide_view.pl?file=../data/20080407/FtgoinTne.dat&query=139&hit=1&index=OMHU1&px=1&section=5&ave_thresh=40) | **439.2452** | **1752.9516** | **1751.9471** | **1.0045** | **0** | **13** | **24** | **1** | **R.YVGGQEHFAHLLILR.D** |
|  | [166](http://www.matrixscience.com/cgi/peptide_view.pl?file=../data/20080407/FtgoinTne.dat&query=166&hit=1&index=OMHU1&px=1&section=5&ave_thresh=40) | **784.6363** | **3134.5161** | **3134.4906** | **0.0255** | **1** | **20** | **2.6** | **1** | **K.TYMLAFDVNDEKNWGLSVYADKPETTK.E** |

|  | |
| --- | --- |
|  | **Proteins matching the same set of peptides:** |

|  | [Q5T539_HUMAN](http://www.matrixscience.com/cgi/protein_view.pl?file=../data/20080407/FtgoinTne.dat&hit=Q5T539_HUMAN&px=1&ave_thresh=40&_sigthreshold=0.05&_server_mudpit_switch=0.001)    **Mass:** 23753    **Score:** 166    **Queries matched:** 13 |
| --- | --- |
|  | Orosomucoid 1.- Homo sapiens (Human). |

|  | [CAA29229](http://www.matrixscience.com/cgi/protein_view.pl?file=../data/20080407/FtgoinTne.dat&hit=CAA29229&px=1&ave_thresh=40&_sigthreshold=0.05&_server_mudpit_switch=0.001)    **Mass:** 23579    **Score:** 166    **Queries matched:** 13 |
| --- | --- |
|  | HSA1GPA1 NID: - Homo sapiens |

Bottom of Form

**Mascot Search Results**

**Spot 129**

**User : A.Srinivasan**

**Email : srini@aiims.ac.in**

**Search title : D:\PE Sciex Data\Projects\Default\Data\Data lo kala spot 6lo kala spot 6.wiff (sample number 1)**

**MS data file : C:\Temp\mas58.tmp**

**Database : MSDB 20060831 (3239079 sequences; 1079594700 residues)**

**Taxonomy : Homo sapiens (human) (148148 sequences)**

**Timestamp : 7 Apr 2008 at 07:19:29 GMT**

| **Protein hits    :** | **[ITHUC1](http://www.matrixscience.com/cgi/master_results.pl?file=../data/20080407/FtgoinEwE.dat#Hit1)** | complement C1 inhibitor precursor [validated] - human |
| --- | --- | --- |
|  | **[OMHU1](http://www.matrixscience.com/cgi/master_results.pl?file=../data/20080407/FtgoinEwE.dat#Hit2)** | alpha-1-acid glycoprotein 1 precursor [validated] - human |
|  | **[CAE98367](http://www.matrixscience.com/cgi/master_results.pl?file=../data/20080407/FtgoinEwE.dat#Hit3)** | AX884253 NID: - Homo sapiens |
|  | **[CAF00770](http://www.matrixscience.com/cgi/master_results.pl?file=../data/20080407/FtgoinEwE.dat#Hit4)** | AX886632 NID: - Homo sapiens |
|  | **[AAQ89125](http://www.matrixscience.com/cgi/master_results.pl?file=../data/20080407/FtgoinEwE.dat#Hit5)** | AY358765 NID: - Homo sapiens |

**Probability Based Mowse Score**

Ions score is -10*Log(P), where P is the probability that the observed match is a random event.
Individual ions scores > 40 indicate identity or extensive homology (p<0.05).
Protein scores are derived from ions scores as a non-probabilistic basis for ranking protein hits.


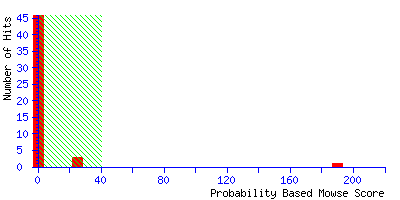


Top of Form

Peptide Summary Report

|  |  |  | [Help](http://www.matrixscience.com/help/results_help.html#FORMAT) |
| --- | --- | --- | --- |
|  | Significance threshold p< | Max. number of hits |  |
|  | Standard scoring  MudPIT scoring | Ions score or expect cut-off | Show sub-sets |
|  | Show pop-ups  Suppress pop-ups | Sort unassigned | Require bold red |

Bottom of Form

Top of Form

**Error tolerant**

| **1.** | [ITHUC1](http://www.matrixscience.com/cgi/protein_view.pl?file=../data/20080407/FtgoinEwE.dat&hit=ITHUC1&px=1&ave_thresh=40&_sigthreshold=0.05&_server_mudpit_switch=0.001)    **Mass:** 55347    **Score:** 190    **Queries matched:** 22   **emPAI:** 0.80 |
| --- | --- |
|  | complement C1 inhibitor precursor [validated] - human |

|  | Check to include this hit in error tolerant search |
| --- | --- |
|  |  |

|  | **Query** | **Observed** | **Mr(expt)** | **Mr(calc)** | **Delta** | **Miss** | **Score** | **Expect** | **Rank** | **Peptide** |
| --- | --- | --- | --- | --- | --- | --- | --- | --- | --- | --- |
|  | [17](http://www.matrixscience.com/cgi/peptide_view.pl?file=../data/20080407/FtgoinEwE.dat&query=17&hit=6&index=ITHUC1&px=1&section=5&ave_thresh=40) | **591.3000** | **590.2927** | **590.3098** | **-0.0170** | **0** | **2** | **6.4e+02** | **6** | **K.AIMEK.L** |
|  | [27](http://www.matrixscience.com/cgi/peptide_view.pl?file=../data/20080407/FtgoinEwE.dat&query=27&hit=2&index=ITHUC1&px=1&section=5&ave_thresh=40) | **404.1992** | **806.3839** | **805.3826** | **1.0012** | **0** | **(16)** | **21** | **2** | **K.VPMMNSK.K** |
|  | [32](http://www.matrixscience.com/cgi/peptide_view.pl?file=../data/20080407/FtgoinEwE.dat&query=32&hit=1&index=ITHUC1&px=1&section=5&ave_thresh=40) | **412.2034** | **822.3923** | **821.3775** | **1.0147** | **0** | **23** | **3.1** | **1** | **K.VPMMNSK.K + Oxidation (M)** |
|  | [36](http://www.matrixscience.com/cgi/peptide_view.pl?file=../data/20080407/FtgoinEwE.dat&query=36&hit=1&index=ITHUC1&px=1&section=5&ave_thresh=40) | **427.2547** | **852.4948** | **852.4316** | **0.0632** | **0** | **14** | **27** | **1** | **K.FPVFMGR.V** |
|  | [48](http://www.matrixscience.com/cgi/peptide_view.pl?file=../data/20080407/FtgoinEwE.dat&query=48&hit=1&index=ITHUC1&px=1&section=5&ave_thresh=40) | **455.7361** | **909.4576** | **909.4556** | **0.0021** | **0** | **25** | **2.2** | **1** | **R.TLYSSSPR.V** |
|  | [54](http://www.matrixscience.com/cgi/peptide_view.pl?file=../data/20080407/FtgoinEwE.dat&query=54&hit=1&index=ITHUC1&px=1&section=5&ave_thresh=40) | **468.2266** | **934.4387** | **934.4371** | **0.0016** | **0** | **11** | **69** | **1** | **R.MEPFHFK.N** |
|  | [82](http://www.matrixscience.com/cgi/peptide_view.pl?file=../data/20080407/FtgoinEwE.dat&query=82&hit=1&index=ITHUC1&px=1&section=5&ave_thresh=40) | **534.2755** | **1066.5365** | **1066.5270** | **0.0096** | **0** | **12** | **56** | **1** | **K.LYHAFSAMK.K** |
|  | [83](http://www.matrixscience.com/cgi/peptide_view.pl?file=../data/20080407/FtgoinEwE.dat&query=83&hit=2&index=ITHUC1&px=1&section=5&ave_thresh=40) | **534.7675** | **1067.5205** | **1066.5270** | **0.9936** | **0** | **(9)** | **1.1e+02** | **2** | **K.LYHAFSAMK.K** |
|  | [86](http://www.matrixscience.com/cgi/peptide_view.pl?file=../data/20080407/FtgoinEwE.dat&query=86&hit=6&index=ITHUC1&px=1&section=5&ave_thresh=40) | **542.2970** | **1082.5793** | **1082.5219** | **0.0575** | **0** | **(3)** | **2.9e+02** | **6** | **K.LYHAFSAMK.K + Oxidation (M)** |
|  | [91](http://www.matrixscience.com/cgi/peptide_view.pl?file=../data/20080407/FtgoinEwE.dat&query=91&hit=1&index=ITHUC1&px=1&section=5&ave_thresh=40) | **558.8005** | **1115.5864** | **1115.5822** | **0.0042** | **0** | **50** | **0.0065** | **1** | **R.LLDSLPSDTR.L** |
|  | [107](http://www.matrixscience.com/cgi/peptide_view.pl?file=../data/20080407/FtgoinEwE.dat&query=107&hit=1&index=ITHUC1&px=1&section=5&ave_thresh=40) | **593.3532** | **1184.6919** | **1184.6917** | **0.0002** | **0** | **25** | **2.3** | **1** | **K.FQPTLLTLPR.I** |
|  | [116](http://www.matrixscience.com/cgi/peptide_view.pl?file=../data/20080407/FtgoinEwE.dat&query=116&hit=2&index=ITHUC1&px=1&section=5&ave_thresh=40) | **609.8000** | **1217.5854** | **1217.5863** | **-0.0009** | **0** | **(9)** | **91** | **2** | **K.DFTCVHQALK.G** |
|  | [117](http://www.matrixscience.com/cgi/peptide_view.pl?file=../data/20080407/FtgoinEwE.dat&query=117&hit=3&index=ITHUC1&px=1&section=5&ave_thresh=40) | **406.8762** | **1217.6068** | **1217.5863** | **0.0205** | **0** | **(11)** | **52** | **3** | **K.DFTCVHQALK.G** |
|  | [118](http://www.matrixscience.com/cgi/peptide_view.pl?file=../data/20080407/FtgoinEwE.dat&query=118&hit=1&index=ITHUC1&px=1&section=5&ave_thresh=40) | **407.2053** | **1218.5941** | **1217.5863** | **1.0078** | **0** | **18** | **10** | **1** | **K.DFTCVHQALK.G** |
|  | [127](http://www.matrixscience.com/cgi/peptide_view.pl?file=../data/20080407/FtgoinEwE.dat&query=127&hit=1&index=ITHUC1&px=1&section=5&ave_thresh=40) | **632.8465** | **1263.6785** | **1263.6710** | **0.0075** | **0** | **75** | **2e-05** | **1** | **K.TNLESILSYPK.D** |
|  | [139](http://www.matrixscience.com/cgi/peptide_view.pl?file=../data/20080407/FtgoinEwE.dat&query=139&hit=1&index=ITHUC1&px=1&section=5&ave_thresh=40) | **659.4127** | **1316.8109** | **1316.8067** | **0.0042** | **0** | **62** | **0.00044** | **1** | **R.LVLLNAIYLSAK.W** |
|  | [153](http://www.matrixscience.com/cgi/peptide_view.pl?file=../data/20080407/FtgoinEwE.dat&query=153&hit=1&index=ITHUC1&px=1&section=5&ave_thresh=40) | **478.2602** | **1431.7589** | **1430.7558** | **1.0031** | **0** | **28** | **0.92** | **1** | **K.YPVAHFIDQTLK.A** |
|  | [159](http://www.matrixscience.com/cgi/peptide_view.pl?file=../data/20080407/FtgoinEwE.dat&query=159&hit=1&index=ITHUC1&px=1&section=5&ave_thresh=40) | **749.3602** | **1496.7058** | **1497.7055** | **-0.9996** | **0** | **38** | **0.083** | **1** | **K.VTTSQDMLSIMEK.L + Oxidation (M)** |
|  | [169](http://www.matrixscience.com/cgi/peptide_view.pl?file=../data/20080407/FtgoinEwE.dat&query=169&hit=1&index=ITHUC1&px=1&section=5&ave_thresh=40) | **575.3108** | **1722.9105** | **1722.8896** | **0.0210** | **1** | **24** | **2.1** | **1** | **R.IKVTTSQDMLSIMEK.L** |
|  | [176](http://www.matrixscience.com/cgi/peptide_view.pl?file=../data/20080407/FtgoinEwE.dat&query=176&hit=1&index=ITHUC1&px=1&section=5&ave_thresh=40) | **609.8000** | **1826.3782** | **1825.9687** | **0.4095** | **0** | **39** | **0.038** | **1** | **K.GVTSVSQIFHSPDLAIR.D** |
|  | [177](http://www.matrixscience.com/cgi/peptide_view.pl?file=../data/20080407/FtgoinEwE.dat&query=177&hit=1&index=ITHUC1&px=1&section=5&ave_thresh=40) | **610.0014** | **1826.9824** | **1825.9687** | **1.0138** | **0** | **(23)** | **2.3** | **1** | **K.GVTSVSQIFHSPDLAIR.D** |
|  | [196](http://www.matrixscience.com/cgi/peptide_view.pl?file=../data/20080407/FtgoinEwE.dat&query=196&hit=1&index=ITHUC1&px=1&section=5&ave_thresh=40) | **872.7997** | **2615.3772** | **2614.3948** | **0.9824** | **0** | **43** | **0.015** | **1** | **R.TLLVFEVQQPFLFVLWDQQHK.F** |

|  | |
| --- | --- |
|  | **Proteins matching the same set of peptides:** |

|  | [AAA35613](http://www.matrixscience.com/cgi/protein_view.pl?file=../data/20080407/FtgoinEwE.dat&hit=AAA35613&px=1&ave_thresh=40&_sigthreshold=0.05&_server_mudpit_switch=0.001)    **Mass:** 55375    **Score:** 190    **Queries matched:** 22 |
| --- | --- |
|  | HUMC1INHA NID: - Homo sapiens |

|  | [AAB59387](http://www.matrixscience.com/cgi/protein_view.pl?file=../data/20080407/FtgoinEwE.dat&hit=AAB59387&px=1&ave_thresh=40&_sigthreshold=0.05&_server_mudpit_switch=0.001)    **Mass:** 55346    **Score:** 190    **Queries matched:** 22 |
| --- | --- |
|  | HUMC1INHB NID: - Homo sapiens |

Bottom of Form

**Mascot Search Results**

**Spot 954**

**User : JITENDRA VASHIST**

**Email : jvashist@yahoo.co.uk**

**Search title : H:\mass res\LOKESH 31.5.10\TTR1.wiff (sample number 1)**

**MS data file : C:\DOCUME~1\DRAF40~1.JIT\LOCALS~1\Temp\mas18.tmp**

**Database : NCBInr 20100724 (11505486 sequences; 3925745078 residues)**

**Taxonomy : Homo sapiens (human) (232657 sequences)**

**Timestamp : 27 Jul 2010 at 02:17:25 GMT**

| **Protein hits    :** | **[gi|126030594](http://www.matrixscience.com/cgi/master_results.pl?file=..%2Fdata%2F20100727%2FFtemCacah.dat;report=5#Hit1)** | Chain A, Crystal Structure Of Cys10 Sulfonated Transthyretin |
| --- | --- | --- |
|  | **[gi|5107791](http://www.matrixscience.com/cgi/master_results.pl?file=..%2Fdata%2F20100727%2FFtemCacah.dat;report=5#Hit2)** | Chain A, Leu 55 Pro Transthyretin Crystal Structure |
|  | **[gi|17942890](http://www.matrixscience.com/cgi/master_results.pl?file=..%2Fdata%2F20100727%2FFtemCacah.dat;report=5#Hit3)** | Chain A, An Engineered Transthyretin Monomer That Is Non-Amyloidogenic - Unless Partially Denatured |
|  | **[gi|259045651](http://www.matrixscience.com/cgi/master_results.pl?file=..%2Fdata%2F20100727%2FFtemCacah.dat;report=5#Hit4)** | Chain 1, The X-Ray Crystal Structure Refinements Of Normal Human Transthyretin And The Amyloidogenic Val 30-->met Variant To 1.7 Angstroms Resolution |
|  | **[gi|435476](http://www.matrixscience.com/cgi/master_results.pl?file=..%2Fdata%2F20100727%2FFtemCacah.dat;report=5#Hit5)** | cytokeratin 9 [Homo sapiens] |

**Mascot Score Histogram**

Ions score is -10*Log(P), where P is the probability that the observed match is a random event.
Individual ions scores > 42 indicate identity or extensive homology (p<0.05).
Protein scores are derived from ions scores as a non-probabilistic basis for ranking protein hits.


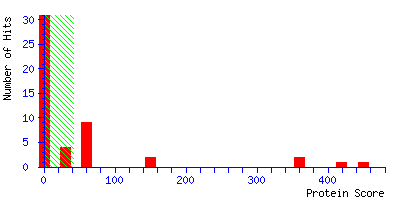


Top of Form

Peptide Summary Report

|  |  |  | [Help](http://www.matrixscience.com/help/msms_summaries_help.html) |
| --- | --- | --- | --- |
|  | Significance threshold p< | Max. number of hits |  |
|  | Standard scoring  MudPIT scoring | Ions score or expect cut-off | Show sub-sets |
|  | Show pop-ups  Suppress pop-ups | Sort unassigned | Require bold red |

Bottom of Form

Top of Form

**Error tolerant**

| **1.** | [gi|126030594](http://www.matrixscience.com/cgi/protein_view.pl?file=../data/20100727/FtemCacah.dat&hit=gi|126030594&db_idx=1&px=1&ave_thresh=42&_ignoreionsscorebelow=0&report=5&_sigthreshold=0.05&_msresflags=1089&_msresflags2=2&percolate=-1&percolate_rt=0)    **Mass:** 13761    **Score:** 449    **Matches:** 13(9)  **Sequences:** 12(9)  **emPAI:** 14.81 |
| --- | --- |
|  | Chain A, Crystal Structure Of Cys10 Sulfonated Transthyretin |

|  | Check to include this hit in error tolerant search |
| --- | --- |
|  |  |

|  | **Query** | **Observed** | **Mr(expt)** | **Mr(calc)** | **Delta** | **Miss** | **Score** | **Expect** | **Rank** | **Unique** | **Peptide** |
| --- | --- | --- | --- | --- | --- | --- | --- | --- | --- | --- | --- |
|  | [451](http://www.matrixscience.com/cgi/peptide_view.pl?file=../data/20100727/FtemCacah.dat&query=451&hit=1&index=gi|126030594&px=1&section=5&ave_thresh=42&_ignoreionsscorebelow=0&report=5&_sigthreshold=0.05&_msresflags=1089&_msresflags2=2&percolate=-1&percolate_rt=0) | **635.3548** | **1268.6951** | **1267.6448** | **1.0502** | **1** | **51** | **0.0082** | **1** |  | **K.VEIDTKSYWK.A** |
|  | [503](http://www.matrixscience.com/cgi/peptide_view.pl?file=../data/20100727/FtemCacah.dat&query=503&hit=1&index=gi|126030594&px=1&section=5&ave_thresh=42&_ignoreionsscorebelow=0&report=5&_sigthreshold=0.05&_msresflags=1089&_msresflags2=2&percolate=-1&percolate_rt=0) | **683.8955** | **1365.7764** | **1365.7517** | **0.0246** | **0** | **71** | **7.5e-05** | **1** |  | **R.GSPAINVAVHVFR.K** |
|  | [510](http://www.matrixscience.com/cgi/peptide_view.pl?file=../data/20100727/FtemCacah.dat&query=510&hit=1&index=gi|126030594&px=1&section=5&ave_thresh=42&_ignoreionsscorebelow=0&report=5&_sigthreshold=0.05&_msresflags=1089&_msresflags2=2&percolate=-1&percolate_rt=0) | **697.3707** | **1392.7268** | **1393.6150** | **-0.8882** | **0** | **86** | **2.3e-06** | **1** |  | **K.AADDTWEPFASGK.T** |
|  | [549](http://www.matrixscience.com/cgi/peptide_view.pl?file=../data/20100727/FtemCacah.dat&query=549&hit=1&index=gi|126030594&px=1&section=5&ave_thresh=42&_ignoreionsscorebelow=0&report=5&_sigthreshold=0.05&_msresflags=1089&_msresflags2=2&percolate=-1&percolate_rt=0) | **492.2786** | **1473.8140** | **1473.7497** | **0.0642** | **1** | **18** | **17** | **1** | **U** | **-.GPTGTGESKGPLMVK.V + Oxidation (M)** |
|  | [560](http://www.matrixscience.com/cgi/peptide_view.pl?file=../data/20100727/FtemCacah.dat&query=560&hit=1&index=gi|126030594&px=1&section=5&ave_thresh=42&_ignoreionsscorebelow=0&report=5&_sigthreshold=0.05&_msresflags=1089&_msresflags2=2&percolate=-1&percolate_rt=0) | **747.9383** | **1493.8621** | **1493.8467** | **0.0154** | **1** | **90** | **8.5e-07** | **1** |  | **R.GSPAINVAVHVFRK.A** |
|  | [571](http://www.matrixscience.com/cgi/peptide_view.pl?file=../data/20100727/FtemCacah.dat&query=571&hit=1&index=gi|126030594&px=1&section=5&ave_thresh=42&_ignoreionsscorebelow=0&report=5&_sigthreshold=0.05&_msresflags=1089&_msresflags2=2&percolate=-1&percolate_rt=0) | **761.8779** | **1521.7412** | **1521.7100** | **0.0312** | **1** | **97** | **2e-07** | **1** |  | **R.KAADDTWEPFASGK.T** |
|  | [594](http://www.matrixscience.com/cgi/peptide_view.pl?file=../data/20100727/FtemCacah.dat&query=594&hit=2&index=gi|126030594&px=1&section=5&ave_thresh=42&_ignoreionsscorebelow=0&report=5&_sigthreshold=0.05&_msresflags=1089&_msresflags2=2&percolate=-1&percolate_rt=0) | **526.2709** | **1575.7909** | **1576.7589** | **-0.9680** | **1** | **32** | **0.52** | **1** | **U** | **-.GPTGTGESKCPLMVK.V + Oxidation (M)** |
|  | [654](http://www.matrixscience.com/cgi/peptide_view.pl?file=../data/20100727/FtemCacah.dat&query=654&hit=1&index=gi|126030594&px=1&section=5&ave_thresh=42&_ignoreionsscorebelow=0&report=5&_sigthreshold=0.05&_msresflags=1089&_msresflags2=2&percolate=-1&percolate_rt=0) | **1181.1312** | **2360.2478** | **2359.2311** | **1.0167** | **0** | **43** | **0.031** | **1** |  | **R.YTIAALLSPYSYSTTAVVTNPK.E** |
|  | [662](http://www.matrixscience.com/cgi/peptide_view.pl?file=../data/20100727/FtemCacah.dat&query=662&hit=1&index=gi|126030594&px=1&section=5&ave_thresh=42&_ignoreionsscorebelow=0&report=5&_sigthreshold=0.05&_msresflags=1089&_msresflags2=2&percolate=-1&percolate_rt=0) | **818.1043** | **2451.2911** | **2450.1979** | **1.0932** | **0** | **85** | **1.8e-06** | **1** |  | **K.ALGISPFHEHAEVVFTANDSGPR.R** |
|  | [664](http://www.matrixscience.com/cgi/peptide_view.pl?file=../data/20100727/FtemCacah.dat&query=664&hit=1&index=gi|126030594&px=1&section=5&ave_thresh=42&_ignoreionsscorebelow=0&report=5&_sigthreshold=0.05&_msresflags=1089&_msresflags2=2&percolate=-1&percolate_rt=0) | **819.4109** | **2455.2109** | **2454.1438** | **1.0671** | **0** | **68** | **8.9e-05** | **1** |  | **K.TSESGELHGLTTEEEFVEGIYK.V** |
|  | [666](http://www.matrixscience.com/cgi/peptide_view.pl?file=../data/20100727/FtemCacah.dat&query=666&hit=1&index=gi|126030594&px=1&section=5&ave_thresh=42&_ignoreionsscorebelow=0&report=5&_sigthreshold=0.05&_msresflags=1089&_msresflags2=2&percolate=-1&percolate_rt=0) | **819.4197** | **2455.2372** | **2454.1438** | **1.0934** | **0** | **(12)** | **35** | **1** |  | **K.TSESGELHGLTTEEEFVEGIYK.V** |
|  | [673](http://www.matrixscience.com/cgi/peptide_view.pl?file=../data/20100727/FtemCacah.dat&query=673&hit=1&index=gi|126030594&px=1&section=5&ave_thresh=42&_ignoreionsscorebelow=0&report=5&_sigthreshold=0.05&_msresflags=1089&_msresflags2=2&percolate=-1&percolate_rt=0) | **830.7868** | **2489.3386** | **2488.2737** | **1.0649** | **1** | **19** | **6.5** | **1** |  | **R.YTIAALLSPYSYSTTAVVTNPKE.-** |
|  | [674](http://www.matrixscience.com/cgi/peptide_view.pl?file=../data/20100727/FtemCacah.dat&query=674&hit=1&index=gi|126030594&px=1&section=5&ave_thresh=42&_ignoreionsscorebelow=0&report=5&_sigthreshold=0.05&_msresflags=1089&_msresflags2=2&percolate=-1&percolate_rt=0) | **839.7993** | **2516.3761** | **2515.3322** | **1.0439** | **1** | **76** | **1.2e-05** | **1** |  | **R.RYTIAALLSPYSYSTTAVVTNPK.E** |

Bottom of Form

**Mascot Search Results**

**Spot 958**

**User : JITENDRA VASHIST**

**Email : jvashist@yahoo.co.uk**

**Search title : H:\mass res\LOKESH 31.5.10\TTR1A.wiff (sample number 1)**

**MS data file : C:\DOCUME~1\DRAF40~1.JIT\LOCALS~1\Temp\mas1B.tmp**

**Database : NCBInr 20100724 (11505486 sequences; 3925745078 residues)**

**Taxonomy : Homo sapiens (human) (232657 sequences)**

**Timestamp : 27 Jul 2010 at 02:25:42 GMT**

| **Protein hits    :** | **[gi|126030594](http://www.matrixscience.com/cgi/master_results.pl?file=..%2Fdata%2F20100727%2FFtemCacte.dat;report=5#Hit1)** | Chain A, Crystal Structure Of Cys10 Sulfonated Transthyretin |
| --- | --- | --- |
|  | **[gi|5107791](http://www.matrixscience.com/cgi/master_results.pl?file=..%2Fdata%2F20100727%2FFtemCacte.dat;report=5#Hit2)** | Chain A, Leu 55 Pro Transthyretin Crystal Structure |
|  | **[gi|17942890](http://www.matrixscience.com/cgi/master_results.pl?file=..%2Fdata%2F20100727%2FFtemCacte.dat;report=5#Hit3)** | Chain A, An Engineered Transthyretin Monomer That Is Non-Amyloidogenic - Unless Partially Denatured |
|  | **[gi|114319005](http://www.matrixscience.com/cgi/master_results.pl?file=..%2Fdata%2F20100727%2FFtemCacte.dat;report=5#Hit4)** | transthyretin [Homo sapiens] |
|  | **[gi|259045651](http://www.matrixscience.com/cgi/master_results.pl?file=..%2Fdata%2F20100727%2FFtemCacte.dat;report=5#Hit5)** | Chain 1, The X-Ray Crystal Structure Refinements Of Normal Human Transthyretin And The Amyloidogenic Val 30-->met Variant To 1.7 Angstroms Resolution |

**Mascot Score Histogram**

Ions score is -10*Log(P), where P is the probability that the observed match is a random event.
Individual ions scores > 42 indicate identity or extensive homology (p<0.05).
Protein scores are derived from ions scores as a non-probabilistic basis for ranking protein hits.


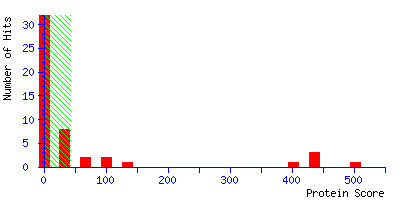


Top of Form

Peptide Summary Report

|  |  |  | [Help](http://www.matrixscience.com/help/msms_summaries_help.html) |
| --- | --- | --- | --- |
|  | Significance threshold p< | Max. number of hits |  |
|  | Standard scoring  MudPIT scoring | Ions score or expect cut-off | Show sub-sets |
|  | Show pop-ups  Suppress pop-ups | Sort unassigned | Require bold red |

Bottom of Form

Top of Form

**Error tolerant**

| **1.** | [gi|126030594](http://www.matrixscience.com/cgi/protein_view.pl?file=../data/20100727/FtemCacte.dat&hit=gi|126030594&db_idx=1&px=1&ave_thresh=42&_ignoreionsscorebelow=0&report=5&_sigthreshold=0.05&_msresflags=1089&_msresflags2=2&percolate=-1&percolate_rt=0)    **Mass:** 13761    **Score:** 503    **Matches:** 12(10)  **Sequences:** 9(7)  **emPAI:** 6.45 |
| --- | --- |
|  | Chain A, Crystal Structure Of Cys10 Sulfonated Transthyretin |

|  | Check to include this hit in error tolerant search |
| --- | --- |
|  |  |

|  | **Query** | **Observed** | **Mr(expt)** | **Mr(calc)** | **Delta** | **Miss** | **Score** | **Expect** | **Rank** | **Unique** | **Peptide** |
| --- | --- | --- | --- | --- | --- | --- | --- | --- | --- | --- | --- |
|  | [462](http://www.matrixscience.com/cgi/peptide_view.pl?file=../data/20100727/FtemCacte.dat&query=462&hit=1&index=gi|126030594&px=1&section=5&ave_thresh=42&_ignoreionsscorebelow=0&report=5&_sigthreshold=0.05&_msresflags=1089&_msresflags2=2&percolate=-1&percolate_rt=0) | **683.8951** | **1365.7757** | **1365.7517** | **0.0240** | **0** | **74** | **3.5e-05** | **1** |  | **R.GSPAINVAVHVFR.K** |
|  | [463](http://www.matrixscience.com/cgi/peptide_view.pl?file=../data/20100727/FtemCacte.dat&query=463&hit=1&index=gi|126030594&px=1&section=5&ave_thresh=42&_ignoreionsscorebelow=0&report=5&_sigthreshold=0.05&_msresflags=1089&_msresflags2=2&percolate=-1&percolate_rt=0) | **684.3916** | **1366.7687** | **1365.7517** | **1.0170** | **0** | **(48)** | **0.019** | **1** |  | **R.GSPAINVAVHVFR.K** |
|  | [470](http://www.matrixscience.com/cgi/peptide_view.pl?file=../data/20100727/FtemCacte.dat&query=470&hit=1&index=gi|126030594&px=1&section=5&ave_thresh=42&_ignoreionsscorebelow=0&report=5&_sigthreshold=0.05&_msresflags=1089&_msresflags2=2&percolate=-1&percolate_rt=0) | **692.3883** | **1382.7621** | **1383.8272** | **-1.0651** | **1** | **23** | **5.2** | **1** | **U** | **K.KPLMVKVLDAVR.G + Oxidation (M)** |
|  | [473](http://www.matrixscience.com/cgi/peptide_view.pl?file=../data/20100727/FtemCacte.dat&query=473&hit=1&index=gi|126030594&px=1&section=5&ave_thresh=42&_ignoreionsscorebelow=0&report=5&_sigthreshold=0.05&_msresflags=1089&_msresflags2=2&percolate=-1&percolate_rt=0) | **697.8249** | **1393.6352** | **1393.6150** | **0.0202** | **0** | **80** | **9.6e-06** | **1** |  | **K.AADDTWEPFASGK.T** |
|  | [515](http://www.matrixscience.com/cgi/peptide_view.pl?file=../data/20100727/FtemCacte.dat&query=515&hit=1&index=gi|126030594&px=1&section=5&ave_thresh=42&_ignoreionsscorebelow=0&report=5&_sigthreshold=0.05&_msresflags=1089&_msresflags2=2&percolate=-1&percolate_rt=0) | **747.9448** | **1493.8750** | **1493.8467** | **0.0283** | **1** | **71** | **7.8e-05** | **1** |  | **R.GSPAINVAVHVFRK.A** |
|  | [524](http://www.matrixscience.com/cgi/peptide_view.pl?file=../data/20100727/FtemCacte.dat&query=524&hit=1&index=gi|126030594&px=1&section=5&ave_thresh=42&_ignoreionsscorebelow=0&report=5&_sigthreshold=0.05&_msresflags=1089&_msresflags2=2&percolate=-1&percolate_rt=0) | **761.8735** | **1521.7324** | **1521.7100** | **0.0225** | **1** | **131** | **7.3e-11** | **1** |  | **R.KAADDTWEPFASGK.T** |
|  | [600](http://www.matrixscience.com/cgi/peptide_view.pl?file=../data/20100727/FtemCacte.dat&query=600&hit=1&index=gi|126030594&px=1&section=5&ave_thresh=42&_ignoreionsscorebelow=0&report=5&_sigthreshold=0.05&_msresflags=1089&_msresflags2=2&percolate=-1&percolate_rt=0) | **1181.1316** | **2360.2487** | **2359.2311** | **1.0176** | **0** | **40** | **0.057** | **1** |  | **R.YTIAALLSPYSYSTTAVVTNPK.E** |
|  | [606](http://www.matrixscience.com/cgi/peptide_view.pl?file=../data/20100727/FtemCacte.dat&query=606&hit=2&index=gi|126030594&px=1&section=5&ave_thresh=42&_ignoreionsscorebelow=0&report=5&_sigthreshold=0.05&_msresflags=1089&_msresflags2=2&percolate=-1&percolate_rt=0) | **818.0922** | **2451.2547** | **2450.1979** | **1.0568** | **0** | **75** | **1.7e-05** | **2** |  | **K.ALGISPFHEHAEVVFTANDSGPR.R** |
|  | [607](http://www.matrixscience.com/cgi/peptide_view.pl?file=../data/20100727/FtemCacte.dat&query=607&hit=1&index=gi|126030594&px=1&section=5&ave_thresh=42&_ignoreionsscorebelow=0&report=5&_sigthreshold=0.05&_msresflags=1089&_msresflags2=2&percolate=-1&percolate_rt=0) | **819.4052** | **2455.1939** | **2454.1438** | **1.0501** | **0** | **(44)** | **0.02** | **1** |  | **K.TSESGELHGLTTEEEFVEGIYK.V** |
|  | [608](http://www.matrixscience.com/cgi/peptide_view.pl?file=../data/20100727/FtemCacte.dat&query=608&hit=1&index=gi|126030594&px=1&section=5&ave_thresh=42&_ignoreionsscorebelow=0&report=5&_sigthreshold=0.05&_msresflags=1089&_msresflags2=2&percolate=-1&percolate_rt=0) | **819.4056** | **2455.1951** | **2454.1438** | **1.0513** | **0** | **(47)** | **0.011** | **1** |  | **K.TSESGELHGLTTEEEFVEGIYK.V** |
|  | [609](http://www.matrixscience.com/cgi/peptide_view.pl?file=../data/20100727/FtemCacte.dat&query=609&hit=1&index=gi|126030594&px=1&section=5&ave_thresh=42&_ignoreionsscorebelow=0&report=5&_sigthreshold=0.05&_msresflags=1089&_msresflags2=2&percolate=-1&percolate_rt=0) | **819.4066** | **2455.1979** | **2454.1438** | **1.0541** | **0** | **71** | **4.6e-05** | **1** |  | **K.TSESGELHGLTTEEEFVEGIYK.V** |
|  | [618](http://www.matrixscience.com/cgi/peptide_view.pl?file=../data/20100727/FtemCacte.dat&query=618&hit=1&index=gi|126030594&px=1&section=5&ave_thresh=42&_ignoreionsscorebelow=0&report=5&_sigthreshold=0.05&_msresflags=1089&_msresflags2=2&percolate=-1&percolate_rt=0) | **839.7968** | **2516.3685** | **2515.3322** | **1.0363** | **1** | **84** | **1.7e-06** | **1** |  | **R.RYTIAALLSPYSYSTTAVVTNPK.E** |

Bottom of Form

**Mascot Search Results**

**Spot 1050**

**User : JITENDRA VASHIST**

**Email : jvashist@yahoo.co.uk**

**Search title : H:\mass res\LOKESH 31.5.10\TTR2.wiff (sample number 1)**

**MS data file : C:\DOCUME~1\DRAF40~1.JIT\LOCALS~1\Temp\mas1D.tmp**

**Database : NCBInr 20100724 (11505486 sequences; 3925745078 residues)**

**Taxonomy : Homo sapiens (human) (232657 sequences)**

**Timestamp : 27 Jul 2010 at 02:36:19 GMT**

| **Protein hits    :** | **[gi|7331218](http://www.matrixscience.com/cgi/master_results.pl?file=..%2Fdata%2F20100727%2FFtemCaamt.dat;report=5#Hit1)** | keratin 1 [Homo sapiens] |
| --- | --- | --- |
|  | **[gi|219978](http://www.matrixscience.com/cgi/master_results.pl?file=..%2Fdata%2F20100727%2FFtemCaamt.dat;report=5#Hit2)** | prealbumin [Homo sapiens] |
|  | **[gi|17942890](http://www.matrixscience.com/cgi/master_results.pl?file=..%2Fdata%2F20100727%2FFtemCaamt.dat;report=5#Hit3)** | Chain A, An Engineered Transthyretin Monomer That Is Non-Amyloidogenic - Unless Partially Denatured |
|  | **[gi|435476](http://www.matrixscience.com/cgi/master_results.pl?file=..%2Fdata%2F20100727%2FFtemCaamt.dat;report=5#Hit4)** | cytokeratin 9 [Homo sapiens] |
|  | **[gi|28317](http://www.matrixscience.com/cgi/master_results.pl?file=..%2Fdata%2F20100727%2FFtemCaamt.dat;report=5#Hit5)** | unnamed protein product [Homo sapiens] |

**Mascot Score Histogram**

Ions score is -10*Log(P), where P is the probability that the observed match is a random event.
Individual ions scores > 43 indicate identity or extensive homology (p<0.05).
Protein scores are derived from ions scores as a non-probabilistic basis for ranking protein hits.


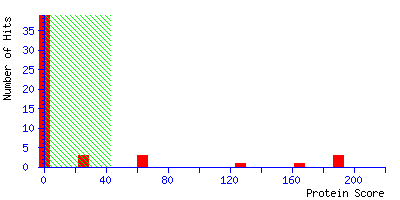


Top of Form

Peptide Summary Report

|  |  |  | [Help](http://www.matrixscience.com/help/msms_summaries_help.html) |
| --- | --- | --- | --- |
|  | Significance threshold p< | Max. number of hits |  |
|  | Standard scoring  MudPIT scoring | Ions score or expect cut-off | Show sub-sets |
|  | Show pop-ups  Suppress pop-ups | Sort unassigned | Require bold red |

Bottom of Form

Top of Form

**Error tolerant**

| **1.** | [gi|7331218](http://www.matrixscience.com/cgi/protein_view.pl?file=../data/20100727/FtemCaamt.dat&hit=gi|7331218&db_idx=1&px=1&ave_thresh=43&_ignoreionsscorebelow=0&report=5&_sigthreshold=0.05&_msresflags=1089&_msresflags2=2&percolate=-1&percolate_rt=0)    **Mass:** 66149    **Score:** 190    **Matches:** 12(7)  **Sequences:** 10(6)  **emPAI:** 0.73 |
| --- | --- |
|  | keratin 1 [Homo sapiens] |

|  | Check to include this hit in error tolerant search |
| --- | --- |
|  |  |

|  | **Query** | **Observed** | **Mr(expt)** | **Mr(calc)** | **Delta** | **Miss** | **Score** | **Expect** | **Rank** | **Unique** | **Peptide** |
| --- | --- | --- | --- | --- | --- | --- | --- | --- | --- | --- | --- |
|  | [271](http://www.matrixscience.com/cgi/peptide_view.pl?file=../data/20100727/FtemCaamt.dat&query=271&hit=1&index=gi|7331218&px=1&section=5&ave_thresh=43&_ignoreionsscorebelow=0&report=5&_sigthreshold=0.05&_msresflags=1089&_msresflags2=2&percolate=-1&percolate_rt=0) | **487.2901** | **972.5657** | **972.5240** | **0.0417** | **0** | **60** | **0.0013** | **1** |  | **K.IEISELNR.V** |
|  | [325](http://www.matrixscience.com/cgi/peptide_view.pl?file=../data/20100727/FtemCaamt.dat&query=325&hit=1&index=gi|7331218&px=1&section=5&ave_thresh=43&_ignoreionsscorebelow=0&report=5&_sigthreshold=0.05&_msresflags=1089&_msresflags2=2&percolate=-1&percolate_rt=0) | **517.2858** | **1032.5570** | **1032.5087** | **0.0482** | **0** | **32** | **0.86** | **1** | **U** | **R.TLLEGEESR.M** |
|  | [407](http://www.matrixscience.com/cgi/peptide_view.pl?file=../data/20100727/FtemCaamt.dat&query=407&hit=1&index=gi|7331218&px=1&section=5&ave_thresh=43&_ignoreionsscorebelow=0&report=5&_sigthreshold=0.05&_msresflags=1089&_msresflags2=2&percolate=-1&percolate_rt=0) | **590.3300** | **1178.6454** | **1178.5931** | **0.0522** | **0** | **62** | **0.00077** | **1** |  | **K.YEELQITAGR.H** |
|  | [461](http://www.matrixscience.com/cgi/peptide_view.pl?file=../data/20100727/FtemCaamt.dat&query=461&hit=1&index=gi|7331218&px=1&section=5&ave_thresh=43&_ignoreionsscorebelow=0&report=5&_sigthreshold=0.05&_msresflags=1089&_msresflags2=2&percolate=-1&percolate_rt=0) | **633.3533** | **1264.6920** | **1264.6299** | **0.0621** | **0** | **50** | **0.0099** | **1** | **U** | **R.TNAENEFVTIK.K** |
|  | [482](http://www.matrixscience.com/cgi/peptide_view.pl?file=../data/20100727/FtemCaamt.dat&query=482&hit=1&index=gi|7331218&px=1&section=5&ave_thresh=43&_ignoreionsscorebelow=0&report=5&_sigthreshold=0.05&_msresflags=1089&_msresflags2=2&percolate=-1&percolate_rt=0) | **650.8007** | **1299.5869** | **1299.5224** | **0.0645** | **0** | **(47)** | **0.02** | **1** | **U** | **K.NMQDMVEDYR.N** |
|  | [485](http://www.matrixscience.com/cgi/peptide_view.pl?file=../data/20100727/FtemCaamt.dat&query=485&hit=1&index=gi|7331218&px=1&section=5&ave_thresh=43&_ignoreionsscorebelow=0&report=5&_sigthreshold=0.05&_msresflags=1089&_msresflags2=2&percolate=-1&percolate_rt=0) | **651.8868** | **1301.7591** | **1301.7078** | **0.0513** | **0** | **54** | **0.0037** | **1** | **U** | **R.SLDLDSIIAEVK.A** |
|  | [500](http://www.matrixscience.com/cgi/peptide_view.pl?file=../data/20100727/FtemCaamt.dat&query=500&hit=1&index=gi|7331218&px=1&section=5&ave_thresh=43&_ignoreionsscorebelow=0&report=5&_sigthreshold=0.05&_msresflags=1089&_msresflags2=2&percolate=-1&percolate_rt=0) | **658.7939** | **1315.5732** | **1315.5173** | **0.0559** | **0** | **50** | **0.012** | **1** | **U** | **K.NMQDMVEDYR.N + Oxidation (M)** |
|  | [512](http://www.matrixscience.com/cgi/peptide_view.pl?file=../data/20100727/FtemCaamt.dat&query=512&hit=1&index=gi|7331218&px=1&section=5&ave_thresh=43&_ignoreionsscorebelow=0&report=5&_sigthreshold=0.05&_msresflags=1089&_msresflags2=2&percolate=-1&percolate_rt=0) | **666.7929** | **1331.5713** | **1331.5122** | **0.0591** | **0** | **(26)** | **3** | **1** | **U** | **K.NMQDMVEDYR.N + 2 Oxidation (M)** |
|  | [523](http://www.matrixscience.com/cgi/peptide_view.pl?file=../data/20100727/FtemCaamt.dat&query=523&hit=1&index=gi|7331218&px=1&section=5&ave_thresh=43&_ignoreionsscorebelow=0&report=5&_sigthreshold=0.05&_msresflags=1089&_msresflags2=2&percolate=-1&percolate_rt=0) | **679.3777** | **1356.7409** | **1356.6885** | **0.0525** | **0** | **65** | **0.00033** | **1** | **U** | **K.LNDLEDALQQAK.E** |
|  | [574](http://www.matrixscience.com/cgi/peptide_view.pl?file=../data/20100727/FtemCaamt.dat&query=574&hit=1&index=gi|7331218&px=1&section=5&ave_thresh=43&_ignoreionsscorebelow=0&report=5&_sigthreshold=0.05&_msresflags=1089&_msresflags2=2&percolate=-1&percolate_rt=0) | **738.4158** | **1474.8171** | **1474.7780** | **0.0391** | **0** | **26** | **2.7** | **1** |  | **R.FLEQQNQVLQTK.W** |
|  | [621](http://www.matrixscience.com/cgi/peptide_view.pl?file=../data/20100727/FtemCaamt.dat&query=621&hit=1&index=gi|7331218&px=1&section=5&ave_thresh=43&_ignoreionsscorebelow=0&report=5&_sigthreshold=0.05&_msresflags=1089&_msresflags2=2&percolate=-1&percolate_rt=0) | **858.9478** | **1715.8810** | **1715.8438** | **0.0372** | **0** | **34** | **0.3** | **1** | **U** | **K.QISNLQQSISDAEQR.G** |
|  | [625](http://www.matrixscience.com/cgi/peptide_view.pl?file=../data/20100727/FtemCaamt.dat&query=625&hit=10&index=gi|7331218&px=1&section=5&ave_thresh=43&_ignoreionsscorebelow=0&report=5&_sigthreshold=0.05&_msresflags=1089&_msresflags2=2&percolate=-1&percolate_rt=0) | **584.6239** | **1750.8500** | **1750.8195** | **0.0305** | **1** | **6** | **2e+02** | **10** | **U** | **R.GSGGGSSGGSIGGRGSSSGGVK.S** |

|  | |
| --- | --- |
|  | **Proteins matching the same set of peptides:** |

|  | [gi|11935049](http://www.matrixscience.com/cgi/protein_view.pl?file=../data/20100727/FtemCaamt.dat&hit=gi|11935049&db_idx=1&px=1&ave_thresh=43&_ignoreionsscorebelow=0&report=5&_sigthreshold=0.05&_msresflags=1089&_msresflags2=2&percolate=-1&percolate_rt=0)    **Mass:** 66198    **Score:** 190    **Matches:** 12(7)  **Sequences:** 10(6) |
| --- | --- |
|  | keratin 1 [Homo sapiens] |

|  | [gi|119395750](http://www.matrixscience.com/cgi/protein_view.pl?file=../data/20100727/FtemCaamt.dat&hit=gi|119395750&db_idx=1&px=1&ave_thresh=43&_ignoreionsscorebelow=0&report=5&_sigthreshold=0.05&_msresflags=1089&_msresflags2=2&percolate=-1&percolate_rt=0)    **Mass:** 66170    **Score:** 190    **Matches:** 12(7)  **Sequences:** 10(6) |
| --- | --- |
|  | keratin, type II cytoskeletal 1 [Homo sapiens]   |  | [gi|219978](http://www.matrixscience.com/cgi/protein_view.pl?file=../data/20100727/FtemCaamt.dat&hit=gi|219978&db_idx=1&px=1&ave_thresh=43&_ignoreionsscorebelow=0&report=5&_sigthreshold=0.05&_msresflags=1089&_msresflags2=2&percolate=-1&percolate_rt=0)    **Mass:** 16023    **Score:** 188    **Matches:** 5(3)  **Sequences:** 5(3)  **emPAI:** 1.39 | | --- | --- | |  | prealbumin [Homo sapiens] |  |  | Check to include this hit in error tolerant search | | --- | --- | |  |  |  |  | **Query** | **Observed** | **Mr(expt)** | **Mr(calc)** | **Delta** | **Miss** | **Score** | **Expect** | **Rank** | **Unique** | **Peptide** | | --- | --- | --- | --- | --- | --- | --- | --- | --- | --- | --- | --- | |  | [467](http://www.matrixscience.com/cgi/peptide_view.pl?file=../data/20100727/FtemCaamt.dat&query=467&hit=2&index=gi|219978&px=1&section=5&ave_thresh=43&_ignoreionsscorebelow=0&report=5&_sigthreshold=0.05&_msresflags=1089&_msresflags2=2&percolate=-1&percolate_rt=0) | **635.3575** | **1268.7004** | **1267.6448** | **1.0555** | **1** | **5** | **3e+02** | **2** |  | **K.VEIDTKSYWK.A** | |  | [550](http://www.matrixscience.com/cgi/peptide_view.pl?file=../data/20100727/FtemCaamt.dat&query=550&hit=1&index=gi|219978&px=1&section=5&ave_thresh=43&_ignoreionsscorebelow=0&report=5&_sigthreshold=0.05&_msresflags=1089&_msresflags2=2&percolate=-1&percolate_rt=0) | **697.3776** | **1392.7407** | **1393.6150** | **-0.8743** | **0** | **96** | **2.7e-07** | **1** |  | **K.AADDTWEPFASGK.T** | |  | [588](http://www.matrixscience.com/cgi/peptide_view.pl?file=../data/20100727/FtemCaamt.dat&query=588&hit=1&index=gi|219978&px=1&section=5&ave_thresh=43&_ignoreionsscorebelow=0&report=5&_sigthreshold=0.05&_msresflags=1089&_msresflags2=2&percolate=-1&percolate_rt=0) | **761.8814** | **1521.7483** | **1521.7100** | **0.0383** | **1** | **30** | **0.87** | **1** |  | **R.KAADDTWEPFASGK.T** | |  | [659](http://www.matrixscience.com/cgi/peptide_view.pl?file=../data/20100727/FtemCaamt.dat&query=659&hit=1&index=gi|219978&px=1&section=5&ave_thresh=43&_ignoreionsscorebelow=0&report=5&_sigthreshold=0.05&_msresflags=1089&_msresflags2=2&percolate=-1&percolate_rt=0) | **818.1086** | **2451.3040** | **2450.1979** | **1.1061** | **0** | **59** | **0.00065** | **1** | **U** | **K.ALGISPFHEHAEVVFTANDSGPR.R** | |  | [660](http://www.matrixscience.com/cgi/peptide_view.pl?file=../data/20100727/FtemCaamt.dat&query=660&hit=1&index=gi|219978&px=1&section=5&ave_thresh=43&_ignoreionsscorebelow=0&report=5&_sigthreshold=0.05&_msresflags=1089&_msresflags2=2&percolate=-1&percolate_rt=0) | **819.4158** | **2455.2255** | **2454.1438** | **1.0817** | **0** | **79** | **6.4e-06** | **1** |  | **K.TSESGELHGLTTEEEFVEGIYK.V** |  |  | | | --- | --- | |  | **Proteins matching the same set of peptides:** |  |  | [gi|386998](http://www.matrixscience.com/cgi/protein_view.pl?file=../data/20100727/FtemCaamt.dat&hit=gi|386998&db_idx=1&px=1&ave_thresh=43&_ignoreionsscorebelow=0&report=5&_sigthreshold=0.05&_msresflags=1089&_msresflags2=2&percolate=-1&percolate_rt=0)    **Mass:** 15932    **Score:** 188    **Matches:** 5(3)  **Sequences:** 5(3) | | --- | --- | |  | prealbumin [Homo sapiens] |  |  | [gi|443295](http://www.matrixscience.com/cgi/protein_view.pl?file=../data/20100727/FtemCaamt.dat&hit=gi|443295&db_idx=1&px=1&ave_thresh=43&_ignoreionsscorebelow=0&report=5&_sigthreshold=0.05&_msresflags=1089&_msresflags2=2&percolate=-1&percolate_rt=0)    **Mass:** 13810    **Score:** 188    **Matches:** 5(3)  **Sequences:** 5(3) | | --- | --- | |  | Chain A, The X-Ray Crystal Structure Refinements Of Normal Human Transthyretin And The Amyloidogenic Val30met Variant To 1.7 Angstroms Resolution |  |  | [gi|443297](http://www.matrixscience.com/cgi/protein_view.pl?file=../data/20100727/FtemCaamt.dat&hit=gi|443297&db_idx=1&px=1&ave_thresh=43&_ignoreionsscorebelow=0&report=5&_sigthreshold=0.05&_msresflags=1089&_msresflags2=2&percolate=-1&percolate_rt=0)    **Mass:** 13840    **Score:** 188    **Matches:** 5(3)  **Sequences:** 5(3) | | --- | --- | |  | Chain A, The X-Ray Crystal Structure Refinements Of Normal Human Transthyretin And The Amyloidogenic Val30met Variant To 1.7 Angstroms Resolution |  |  | [gi|443299](http://www.matrixscience.com/cgi/protein_view.pl?file=../data/20100727/FtemCaamt.dat&hit=gi|443299&db_idx=1&px=1&ave_thresh=43&_ignoreionsscorebelow=0&report=5&_sigthreshold=0.05&_msresflags=1089&_msresflags2=2&percolate=-1&percolate_rt=0)    **Mass:** 13842    **Score:** 188    **Matches:** 5(3)  **Sequences:** 5(3) | | --- | --- | |  | Chain A, The X-Ray Crystal Structure Refinements Of Normal Human Transthyretin And The Amyloidogenic Val30met Variant To 1.7 Angstroms Resolution |  |  | [gi|1827569](http://www.matrixscience.com/cgi/protein_view.pl?file=../data/20100727/FtemCaamt.dat&hit=gi|1827569&db_idx=1&px=1&ave_thresh=43&_ignoreionsscorebelow=0&report=5&_sigthreshold=0.05&_msresflags=1089&_msresflags2=2&percolate=-1&percolate_rt=0)    **Mass:** 13824    **Score:** 188    **Matches:** 5(3)  **Sequences:** 5(3) | | --- | --- | |  | Chain A, Transthyretin-V122I CARDIOMYOPATHIC MUTANT |  |  | [gi|2098255](http://www.matrixscience.com/cgi/protein_view.pl?file=../data/20100727/FtemCaamt.dat&hit=gi|2098255&db_idx=1&px=1&ave_thresh=43&_ignoreionsscorebelow=0&report=5&_sigthreshold=0.05&_msresflags=1089&_msresflags2=2&percolate=-1&percolate_rt=0)    **Mass:** 13806    **Score:** 188    **Matches:** 5(3)  **Sequences:** 5(3) | | --- | --- | |  | Chain A, Tertiary Structures Of Three Amyloidogenic Transthyretin Variants And Implications For Amyloid Fibril Formation |  |  | [gi|3891451](http://www.matrixscience.com/cgi/protein_view.pl?file=../data/20100727/FtemCaamt.dat&hit=gi|3891451&db_idx=1&px=1&ave_thresh=43&_ignoreionsscorebelow=0&report=5&_sigthreshold=0.05&_msresflags=1089&_msresflags2=2&percolate=-1&percolate_rt=0)    **Mass:** 13711    **Score:** 188    **Matches:** 5(3)  **Sequences:** 5(3) | | --- | --- | |  | Chain A, Transthyretin (Del Val122) |  |  | [gi|3891560](http://www.matrixscience.com/cgi/protein_view.pl?file=../data/20100727/FtemCaamt.dat&hit=gi|3891560&db_idx=1&px=1&ave_thresh=43&_ignoreionsscorebelow=0&report=5&_sigthreshold=0.05&_msresflags=1089&_msresflags2=2&percolate=-1&percolate_rt=0)    **Mass:** 13840    **Score:** 188    **Matches:** 5(3)  **Sequences:** 5(3) | | --- | --- | |  | Chain A, Tertiary Structures Of Three Amyloidogenic Transthyretin Variants And Implications For Amyloid Fibril Formation |  |  | [gi|3891562](http://www.matrixscience.com/cgi/protein_view.pl?file=../data/20100727/FtemCaamt.dat&hit=gi|3891562&db_idx=1&px=1&ave_thresh=43&_ignoreionsscorebelow=0&report=5&_sigthreshold=0.05&_msresflags=1089&_msresflags2=2&percolate=-1&percolate_rt=0)    **Mass:** 13840    **Score:** 188    **Matches:** 5(3)  **Sequences:** 5(3) | | --- | --- | |  | Chain A, Tertiary Structures Of Three Amyloidogenic Transthyretin Variants And Implications For Amyloid Fibril Formation |  |  | [gi|4507725](http://www.matrixscience.com/cgi/protein_view.pl?file=../data/20100727/FtemCaamt.dat&hit=gi|4507725&db_idx=1&px=1&ave_thresh=43&_ignoreionsscorebelow=0&report=5&_sigthreshold=0.05&_msresflags=1089&_msresflags2=2&percolate=-1&percolate_rt=0)    **Mass:** 15991    **Score:** 188    **Matches:** 5(3)  **Sequences:** 5(3) | | --- | --- | |  | transthyretin precursor [Homo sapiens] |  |  | [gi|14719497](http://www.matrixscience.com/cgi/protein_view.pl?file=../data/20100727/FtemCaamt.dat&hit=gi|14719497&db_idx=1&px=1&ave_thresh=43&_ignoreionsscorebelow=0&report=5&_sigthreshold=0.05&_msresflags=1089&_msresflags2=2&percolate=-1&percolate_rt=0)    **Mass:** 12671    **Score:** 188    **Matches:** 5(3)  **Sequences:** 5(3) | | --- | --- | |  | Chain A, Transthyretin Thr119met Protein Stabilisation |  |  | [gi|27065112](http://www.matrixscience.com/cgi/protein_view.pl?file=../data/20100727/FtemCaamt.dat&hit=gi|27065112&db_idx=1&px=1&ave_thresh=43&_ignoreionsscorebelow=0&report=5&_sigthreshold=0.05&_msresflags=1089&_msresflags2=2&percolate=-1&percolate_rt=0)    **Mass:** 13807    **Score:** 188    **Matches:** 5(3)  **Sequences:** 5(3) | | --- | --- | |  | Chain A, Crystal Structure Of The Transthyretin Mutant Ttr Y114c- Data Collected At Room Temperature |  |  | [gi|31615374](http://www.matrixscience.com/cgi/protein_view.pl?file=../data/20100727/FtemCaamt.dat&hit=gi|31615374&db_idx=1&px=1&ave_thresh=43&_ignoreionsscorebelow=0&report=5&_sigthreshold=0.05&_msresflags=1089&_msresflags2=2&percolate=-1&percolate_rt=0)    **Mass:** 13718    **Score:** 188    **Matches:** 5(3)  **Sequences:** 5(3) | | --- | --- | |  | Chain A, Crystal Structure Of The Transthyretin Mutant Ttr C10aY114C |  |  | [gi|48145933](http://www.matrixscience.com/cgi/protein_view.pl?file=../data/20100727/FtemCaamt.dat&hit=gi|48145933&db_idx=1&px=1&ave_thresh=43&_ignoreionsscorebelow=0&report=5&_sigthreshold=0.05&_msresflags=1089&_msresflags2=2&percolate=-1&percolate_rt=0)    **Mass:** 15977    **Score:** 188    **Matches:** 5(3)  **Sequences:** 5(3) | | --- | --- | |  | TTR [Homo sapiens] |  |  | [gi|50513594](http://www.matrixscience.com/cgi/protein_view.pl?file=../data/20100727/FtemCaamt.dat&hit=gi|50513594&db_idx=1&px=1&ave_thresh=43&_ignoreionsscorebelow=0&report=5&_sigthreshold=0.05&_msresflags=1089&_msresflags2=2&percolate=-1&percolate_rt=0)    **Mass:** 13918    **Score:** 188    **Matches:** 5(3)  **Sequences:** 5(3) | | --- | --- | |  | Chain A, Crystal Structure Of The Transthyretin Mutant A108yL110E Solved In Space Group P21212 |  |  | [gi|55669575](http://www.matrixscience.com/cgi/protein_view.pl?file=../data/20100727/FtemCaamt.dat&hit=gi|55669575&db_idx=1&px=1&ave_thresh=43&_ignoreionsscorebelow=0&report=5&_sigthreshold=0.05&_msresflags=1089&_msresflags2=2&percolate=-1&percolate_rt=0)    **Mass:** 12836    **Score:** 188    **Matches:** 5(3)  **Sequences:** 5(3) | | --- | --- | |  | Chain A, A Covalent Dimer Of Transthyretin That Affects The Amyloid Pathway |  |  | [gi|62738388](http://www.matrixscience.com/cgi/protein_view.pl?file=../data/20100727/FtemCaamt.dat&hit=gi|62738388&db_idx=1&px=1&ave_thresh=43&_ignoreionsscorebelow=0&report=5&_sigthreshold=0.05&_msresflags=1089&_msresflags2=2&percolate=-1&percolate_rt=0)    **Mass:** 13791    **Score:** 188    **Matches:** 5(3)  **Sequences:** 5(3) | | --- | --- | |  | Chain A, Structure Of Ttr R104h: A Non-Amyloidogenic Variant With Protective Clinical Effects |  |  | [gi|73535906](http://www.matrixscience.com/cgi/protein_view.pl?file=../data/20100727/FtemCaamt.dat&hit=gi|73535906&db_idx=1&px=1&ave_thresh=43&_ignoreionsscorebelow=0&report=5&_sigthreshold=0.05&_msresflags=1089&_msresflags2=2&percolate=-1&percolate_rt=0)    **Mass:** 13941    **Score:** 188    **Matches:** 5(3)  **Sequences:** 5(3) | | --- | --- | |  | Chain A, Crystal Structure Of Human Transthyretin With Bound Iodide |  |  | [gi|114319005](http://www.matrixscience.com/cgi/protein_view.pl?file=../data/20100727/FtemCaamt.dat&hit=gi|114319005&db_idx=1&px=1&ave_thresh=43&_ignoreionsscorebelow=0&report=5&_sigthreshold=0.05&_msresflags=1089&_msresflags2=2&percolate=-1&percolate_rt=0)    **Mass:** 15062    **Score:** 188    **Matches:** 5(3)  **Sequences:** 5(3) | | --- | --- | |  | transthyretin [Homo sapiens] |  |  | [gi|212374952](http://www.matrixscience.com/cgi/protein_view.pl?file=../data/20100727/FtemCaamt.dat&hit=gi|212374952&db_idx=1&px=1&ave_thresh=43&_ignoreionsscorebelow=0&report=5&_sigthreshold=0.05&_msresflags=1089&_msresflags2=2&percolate=-1&percolate_rt=0)    **Mass:** 13798    **Score:** 188    **Matches:** 5(3)  **Sequences:** 5(3) | | --- | --- | |  | Chain A, Crystal Structure Of Transthyretin Variant V20s |  |  | [gi|223674041](http://www.matrixscience.com/cgi/protein_view.pl?file=../data/20100727/FtemCaamt.dat&hit=gi|223674041&db_idx=1&px=1&ave_thresh=43&_ignoreionsscorebelow=0&report=5&_sigthreshold=0.05&_msresflags=1089&_msresflags2=2&percolate=-1&percolate_rt=0)    **Mass:** 13456    **Score:** 188    **Matches:** 5(3)  **Sequences:** 5(3) | | --- | --- | |  | Chain A, Crystal Structure Of Transthyretin In Complex With Iododiflunisal-Betaalaome |  |  | [gi|224510585](http://www.matrixscience.com/cgi/protein_view.pl?file=../data/20100727/FtemCaamt.dat&hit=gi|224510585&db_idx=1&px=1&ave_thresh=43&_ignoreionsscorebelow=0&report=5&_sigthreshold=0.05&_msresflags=1089&_msresflags2=2&percolate=-1&percolate_rt=0)    **Mass:** 12996    **Score:** 188    **Matches:** 5(3)  **Sequences:** 5(3) | | --- | --- | |  | Chain A, Crystal Structure Of The Apo Form Of Human Wild-Type Transthyretin |  |  | [gi|226438187](http://www.matrixscience.com/cgi/protein_view.pl?file=../data/20100727/FtemCaamt.dat&hit=gi|226438187&db_idx=1&px=1&ave_thresh=43&_ignoreionsscorebelow=0&report=5&_sigthreshold=0.05&_msresflags=1089&_msresflags2=2&percolate=-1&percolate_rt=0)    **Mass:** 13784    **Score:** 188    **Matches:** 5(3)  **Sequences:** 5(3) | | --- | --- | |  | Chain A, Crystal Structure Of Transthyretin Variant Y114h |  |  | [gi|253722168](http://www.matrixscience.com/cgi/protein_view.pl?file=../data/20100727/FtemCaamt.dat&hit=gi|253722168&db_idx=1&px=1&ave_thresh=43&_ignoreionsscorebelow=0&report=5&_sigthreshold=0.05&_msresflags=1089&_msresflags2=2&percolate=-1&percolate_rt=0)    **Mass:** 12789    **Score:** 188    **Matches:** 5(3)  **Sequences:** 5(3) | | --- | --- | |  | Chain A, Transthyretin Stability As A Key Factor In Amyloidogenesis |  |  | [gi|259045651](http://www.matrixscience.com/cgi/protein_view.pl?file=../data/20100727/FtemCaamt.dat&hit=gi|259045651&db_idx=1&px=1&ave_thresh=43&_ignoreionsscorebelow=0&report=5&_sigthreshold=0.05&_msresflags=1089&_msresflags2=2&percolate=-1&percolate_rt=0)    **Mass:** 13941    **Score:** 188    **Matches:** 5(3)  **Sequences:** 5(3) | | --- | --- | |  | Chain 1, The X-Ray Crystal Structure Refinements Of Normal Human Transthyretin And The Amyloidogenic Val 30-->met Variant To 1.7 Angstroms Resolution |  |  | [gi|261824993](http://www.matrixscience.com/cgi/protein_view.pl?file=../data/20100727/FtemCaamt.dat&hit=gi|261824993&db_idx=1&px=1&ave_thresh=43&_ignoreionsscorebelow=0&report=5&_sigthreshold=0.05&_msresflags=1089&_msresflags2=2&percolate=-1&percolate_rt=0)    **Mass:** 12738    **Score:** 188    **Matches:** 5(3)  **Sequences:** 5(3) | | --- | --- | |  | Chain A, Crystal Structure Of Human Transthyretin - Wild Type |  |  | [gi|114318993](http://www.matrixscience.com/cgi/protein_view.pl?file=../data/20100727/FtemCaamt.dat&hit=gi|114318993&db_idx=1&px=1&ave_thresh=43&_ignoreionsscorebelow=0&report=5&_sigthreshold=0.05&_msresflags=1089&_msresflags2=2&percolate=-1&percolate_rt=0)    **Mass:** 20300    **Score:** 188    **Matches:** 5(3)  **Sequences:** 5(3) | | --- | --- | |  | transthyretin [Homo sapiens] |  |  | [gi|126030594](http://www.matrixscience.com/cgi/protein_view.pl?file=../data/20100727/FtemCaamt.dat&hit=gi|126030594&db_idx=1&px=1&ave_thresh=43&_ignoreionsscorebelow=0&report=5&_sigthreshold=0.05&_msresflags=1089&_msresflags2=2&percolate=-1&percolate_rt=0)    **Mass:** 13761    **Score:** 188    **Matches:** 5(3)  **Sequences:** 5(3) | | --- | --- | |  | Chain A, Crystal Structure Of Cys10 Sulfonated Transthyretin | |

Bottom of Form

**Mascot Search Results**

**Spot 1049**

**User : JITENDRA VASHIST**

**Email : jvashist@yahoo.co.uk**

**Search title : H:\mass res\LOKESH 31.5.10\TTR2A.wiff (sample number 1)**

**MS data file : C:\DOCUME~1\DRAF40~1.JIT\LOCALS~1\Temp\mas20.tmp**

**Database : NCBInr 20100724 (11505486 sequences; 3925745078 residues)**

**Taxonomy : Homo sapiens (human) (232657 sequences)**

**Timestamp : 27 Jul 2010 at 02:43:37 GMT**

| **Protein hits    :** | **[gi|443295](http://www.matrixscience.com/cgi/master_results.pl?file=..%2Fdata%2F20100727%2FFtemCaaOR.dat;report=5#Hit1)** | Chain A, The X-Ray Crystal Structure Refinements Of Normal Human Transthyretin And The Amyloidogenic Val30met Variant To 1.7 Angstroms Resolution |
| --- | --- | --- |
|  | **[gi|435476](http://www.matrixscience.com/cgi/master_results.pl?file=..%2Fdata%2F20100727%2FFtemCaaOR.dat;report=5#Hit2)** | cytokeratin 9 [Homo sapiens] |
|  | **[gi|7331218](http://www.matrixscience.com/cgi/master_results.pl?file=..%2Fdata%2F20100727%2FFtemCaaOR.dat;report=5#Hit3)** | keratin 1 [Homo sapiens] |
|  | **[gi|28317](http://www.matrixscience.com/cgi/master_results.pl?file=..%2Fdata%2F20100727%2FFtemCaaOR.dat;report=5#Hit4)** | unnamed protein product [Homo sapiens] |
|  | **[gi|181402](http://www.matrixscience.com/cgi/master_results.pl?file=..%2Fdata%2F20100727%2FFtemCaaOR.dat;report=5#Hit5)** | epidermal cytokeratin 2 [Homo sapiens] |

**Mascot Score Histogram**

Ions score is -10*Log(P), where P is the probability that the observed match is a random event.
Individual ions scores > 43 indicate identity or extensive homology (p<0.05).
Protein scores are derived from ions scores as a non-probabilistic basis for ranking protein hits.


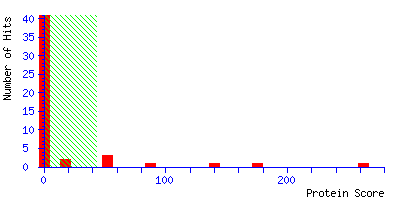


Top of Form

Peptide Summary Report

|  |  |  | [Help](http://www.matrixscience.com/help/msms_summaries_help.html) |
| --- | --- | --- | --- |
|  | Significance threshold p< | Max. number of hits |  |
|  | Standard scoring  MudPIT scoring | Ions score or expect cut-off | Show sub-sets |
|  | Show pop-ups  Suppress pop-ups | Sort unassigned | Require bold red |

Bottom of Form

Top of Form

**Error tolerant**

| **1.** | [gi|443295](http://www.matrixscience.com/cgi/protein_view.pl?file=../data/20100727/FtemCaaOR.dat&hit=gi|443295&db_idx=1&px=1&ave_thresh=43&_ignoreionsscorebelow=0&report=5&_sigthreshold=0.05&_msresflags=1089&_msresflags2=2&percolate=-1&percolate_rt=0)    **Mass:** 13810    **Score:** 262    **Matches:** 5(5)  **Sequences:** 5(5)  **emPAI:** 2.51 |
| --- | --- |
|  | Chain A, The X-Ray Crystal Structure Refinements Of Normal Human Transthyretin And The Amyloidogenic Val30met Variant To 1.7 Angstroms Resolution |

|  | Check to include this hit in error tolerant search |
| --- | --- |
|  |  |

|  | **Query** | **Observed** | **Mr(expt)** | **Mr(calc)** | **Delta** | **Miss** | **Score** | **Expect** | **Rank** | **Unique** | **Peptide** |
| --- | --- | --- | --- | --- | --- | --- | --- | --- | --- | --- | --- |
|  | [610](http://www.matrixscience.com/cgi/peptide_view.pl?file=../data/20100727/FtemCaaOR.dat&query=610&hit=1&index=gi|443295&px=1&section=5&ave_thresh=43&_ignoreionsscorebelow=0&report=5&_sigthreshold=0.05&_msresflags=1089&_msresflags2=2&percolate=-1&percolate_rt=0) | **683.9003** | **1365.7861** | **1365.7517** | **0.0344** | **0** | **69** | **0.00012** | **1** | **U** | **R.GSPAINVAVHVFR.K** |
|  | [622](http://www.matrixscience.com/cgi/peptide_view.pl?file=../data/20100727/FtemCaaOR.dat&query=622&hit=1&index=gi|443295&px=1&section=5&ave_thresh=43&_ignoreionsscorebelow=0&report=5&_sigthreshold=0.05&_msresflags=1089&_msresflags2=2&percolate=-1&percolate_rt=0) | **697.8268** | **1393.6391** | **1393.6150** | **0.0241** | **0** | **75** | **3e-05** | **1** | **U** | **K.AADDTWEPFASGK.T** |
|  | [663](http://www.matrixscience.com/cgi/peptide_view.pl?file=../data/20100727/FtemCaaOR.dat&query=663&hit=1&index=gi|443295&px=1&section=5&ave_thresh=43&_ignoreionsscorebelow=0&report=5&_sigthreshold=0.05&_msresflags=1089&_msresflags2=2&percolate=-1&percolate_rt=0) | **747.9481** | **1493.8816** | **1493.8467** | **0.0349** | **1** | **54** | **0.0034** | **1** | **U** | **R.GSPAINVAVHVFRK.A** |
|  | [671](http://www.matrixscience.com/cgi/peptide_view.pl?file=../data/20100727/FtemCaaOR.dat&query=671&hit=1&index=gi|443295&px=1&section=5&ave_thresh=43&_ignoreionsscorebelow=0&report=5&_sigthreshold=0.05&_msresflags=1089&_msresflags2=2&percolate=-1&percolate_rt=0) | **761.8731** | **1521.7317** | **1521.7100** | **0.0217** | **1** | **97** | **1.9e-07** | **1** | **U** | **R.KAADDTWEPFASGK.T** |
|  | [730](http://www.matrixscience.com/cgi/peptide_view.pl?file=../data/20100727/FtemCaaOR.dat&query=730&hit=1&index=gi|443295&px=1&section=5&ave_thresh=43&_ignoreionsscorebelow=0&report=5&_sigthreshold=0.05&_msresflags=1089&_msresflags2=2&percolate=-1&percolate_rt=0) | **819.4029** | **2455.1869** | **2454.1438** | **1.0431** | **0** | **64** | **0.0002** | **1** | **U** | **K.TSESGELHGLTTEEEFVEGIYK.V** |

|  | |
| --- | --- |
|  | **Proteins matching the same set of peptides:** |

|  | [gi|443297](http://www.matrixscience.com/cgi/protein_view.pl?file=../data/20100727/FtemCaaOR.dat&hit=gi|443297&db_idx=1&px=1&ave_thresh=43&_ignoreionsscorebelow=0&report=5&_sigthreshold=0.05&_msresflags=1089&_msresflags2=2&percolate=-1&percolate_rt=0)    **Mass:** 13840    **Score:** 262    **Matches:** 5(5)  **Sequences:** 5(5) |
| --- | --- |
|  | Chain A, The X-Ray Crystal Structure Refinements Of Normal Human Transthyretin And The Amyloidogenic Val30met Variant To 1.7 Angstroms Resolution |

|  | [gi|1827569](http://www.matrixscience.com/cgi/protein_view.pl?file=../data/20100727/FtemCaaOR.dat&hit=gi|1827569&db_idx=1&px=1&ave_thresh=43&_ignoreionsscorebelow=0&report=5&_sigthreshold=0.05&_msresflags=1089&_msresflags2=2&percolate=-1&percolate_rt=0)    **Mass:** 13824    **Score:** 262    **Matches:** 5(5)  **Sequences:** 5(5) |
| --- | --- |
|  | Chain A, Transthyretin-V122I CARDIOMYOPATHIC MUTANT |

|  | [gi|2098255](http://www.matrixscience.com/cgi/protein_view.pl?file=../data/20100727/FtemCaaOR.dat&hit=gi|2098255&db_idx=1&px=1&ave_thresh=43&_ignoreionsscorebelow=0&report=5&_sigthreshold=0.05&_msresflags=1089&_msresflags2=2&percolate=-1&percolate_rt=0)    **Mass:** 13806    **Score:** 262    **Matches:** 5(5)  **Sequences:** 5(5) |
| --- | --- |
|  | Chain A, Tertiary Structures Of Three Amyloidogenic Transthyretin Variants And Implications For Amyloid Fibril Formation |

|  | [gi|2098257](http://www.matrixscience.com/cgi/protein_view.pl?file=../data/20100727/FtemCaaOR.dat&hit=gi|2098257&db_idx=1&px=1&ave_thresh=43&_ignoreionsscorebelow=0&report=5&_sigthreshold=0.05&_msresflags=1089&_msresflags2=2&percolate=-1&percolate_rt=0)    **Mass:** 13886    **Score:** 262    **Matches:** 5(5)  **Sequences:** 5(5) |
| --- | --- |
|  | Chain A, Tertiary Structures Of Three Amyloidogenic Transthyretin Variants And Implications For Amyloid Fibril Formation |

|  | [gi|3891451](http://www.matrixscience.com/cgi/protein_view.pl?file=../data/20100727/FtemCaaOR.dat&hit=gi|3891451&db_idx=1&px=1&ave_thresh=43&_ignoreionsscorebelow=0&report=5&_sigthreshold=0.05&_msresflags=1089&_msresflags2=2&percolate=-1&percolate_rt=0)    **Mass:** 13711    **Score:** 262    **Matches:** 5(5)  **Sequences:** 5(5) |
| --- | --- |
|  | Chain A, Transthyretin (Del Val122) |

|  | [gi|3891560](http://www.matrixscience.com/cgi/protein_view.pl?file=../data/20100727/FtemCaaOR.dat&hit=gi|3891560&db_idx=1&px=1&ave_thresh=43&_ignoreionsscorebelow=0&report=5&_sigthreshold=0.05&_msresflags=1089&_msresflags2=2&percolate=-1&percolate_rt=0)    **Mass:** 13840    **Score:** 262    **Matches:** 5(5)  **Sequences:** 5(5) |
| --- | --- |
|  | Chain A, Tertiary Structures Of Three Amyloidogenic Transthyretin Variants And Implications For Amyloid Fibril Formation |

|  | [gi|3891562](http://www.matrixscience.com/cgi/protein_view.pl?file=../data/20100727/FtemCaaOR.dat&hit=gi|3891562&db_idx=1&px=1&ave_thresh=43&_ignoreionsscorebelow=0&report=5&_sigthreshold=0.05&_msresflags=1089&_msresflags2=2&percolate=-1&percolate_rt=0)    **Mass:** 13840    **Score:** 262    **Matches:** 5(5)  **Sequences:** 5(5) |
| --- | --- |
|  | Chain A, Tertiary Structures Of Three Amyloidogenic Transthyretin Variants And Implications For Amyloid Fibril Formation |

|  | [gi|4507725](http://www.matrixscience.com/cgi/protein_view.pl?file=../data/20100727/FtemCaaOR.dat&hit=gi|4507725&db_idx=1&px=1&ave_thresh=43&_ignoreionsscorebelow=0&report=5&_sigthreshold=0.05&_msresflags=1089&_msresflags2=2&percolate=-1&percolate_rt=0)    **Mass:** 15991    **Score:** 262    **Matches:** 5(5)  **Sequences:** 5(5) |
| --- | --- |
|  | transthyretin precursor [Homo sapiens] |

|  | [gi|14719497](http://www.matrixscience.com/cgi/protein_view.pl?file=../data/20100727/FtemCaaOR.dat&hit=gi|14719497&db_idx=1&px=1&ave_thresh=43&_ignoreionsscorebelow=0&report=5&_sigthreshold=0.05&_msresflags=1089&_msresflags2=2&percolate=-1&percolate_rt=0)    **Mass:** 12671    **Score:** 262    **Matches:** 5(5)  **Sequences:** 5(5) |
| --- | --- |
|  | Chain A, Transthyretin Thr119met Protein Stabilisation |

|  | [gi|17942890](http://www.matrixscience.com/cgi/protein_view.pl?file=../data/20100727/FtemCaaOR.dat&hit=gi|17942890&db_idx=1&px=1&ave_thresh=43&_ignoreionsscorebelow=0&report=5&_sigthreshold=0.05&_msresflags=1089&_msresflags2=2&percolate=-1&percolate_rt=0)    **Mass:** 13812    **Score:** 262    **Matches:** 5(5)  **Sequences:** 5(5) |
| --- | --- |
|  | Chain A, An Engineered Transthyretin Monomer That Is Non-Amyloidogenic - Unless Partially Denatured |

|  | [gi|27065112](http://www.matrixscience.com/cgi/protein_view.pl?file=../data/20100727/FtemCaaOR.dat&hit=gi|27065112&db_idx=1&px=1&ave_thresh=43&_ignoreionsscorebelow=0&report=5&_sigthreshold=0.05&_msresflags=1089&_msresflags2=2&percolate=-1&percolate_rt=0)    **Mass:** 13807    **Score:** 262    **Matches:** 5(5)  **Sequences:** 5(5) |
| --- | --- |
|  | Chain A, Crystal Structure Of The Transthyretin Mutant Ttr Y114c- Data Collected At Room Temperature |

|  | [gi|31615374](http://www.matrixscience.com/cgi/protein_view.pl?file=../data/20100727/FtemCaaOR.dat&hit=gi|31615374&db_idx=1&px=1&ave_thresh=43&_ignoreionsscorebelow=0&report=5&_sigthreshold=0.05&_msresflags=1089&_msresflags2=2&percolate=-1&percolate_rt=0)    **Mass:** 13718    **Score:** 262    **Matches:** 5(5)  **Sequences:** 5(5) |
| --- | --- |
|  | Chain A, Crystal Structure Of The Transthyretin Mutant Ttr C10aY114C |

|  | [gi|48145933](http://www.matrixscience.com/cgi/protein_view.pl?file=../data/20100727/FtemCaaOR.dat&hit=gi|48145933&db_idx=1&px=1&ave_thresh=43&_ignoreionsscorebelow=0&report=5&_sigthreshold=0.05&_msresflags=1089&_msresflags2=2&percolate=-1&percolate_rt=0)    **Mass:** 15977    **Score:** 262    **Matches:** 5(5)  **Sequences:** 5(5) |
| --- | --- |
|  | TTR [Homo sapiens] |

|  | [gi|50513594](http://www.matrixscience.com/cgi/protein_view.pl?file=../data/20100727/FtemCaaOR.dat&hit=gi|50513594&db_idx=1&px=1&ave_thresh=43&_ignoreionsscorebelow=0&report=5&_sigthreshold=0.05&_msresflags=1089&_msresflags2=2&percolate=-1&percolate_rt=0)    **Mass:** 13918    **Score:** 262    **Matches:** 5(5)  **Sequences:** 5(5) |
| --- | --- |
|  | Chain A, Crystal Structure Of The Transthyretin Mutant A108yL110E Solved In Space Group P21212 |

|  | [gi|55669575](http://www.matrixscience.com/cgi/protein_view.pl?file=../data/20100727/FtemCaaOR.dat&hit=gi|55669575&db_idx=1&px=1&ave_thresh=43&_ignoreionsscorebelow=0&report=5&_sigthreshold=0.05&_msresflags=1089&_msresflags2=2&percolate=-1&percolate_rt=0)    **Mass:** 12836    **Score:** 262    **Matches:** 5(5)  **Sequences:** 5(5) |
| --- | --- |
|  | Chain A, A Covalent Dimer Of Transthyretin That Affects The Amyloid Pathway |

|  | [gi|62738386](http://www.matrixscience.com/cgi/protein_view.pl?file=../data/20100727/FtemCaaOR.dat&hit=gi|62738386&db_idx=1&px=1&ave_thresh=43&_ignoreionsscorebelow=0&report=5&_sigthreshold=0.05&_msresflags=1089&_msresflags2=2&percolate=-1&percolate_rt=0)    **Mass:** 13794    **Score:** 262    **Matches:** 5(5)  **Sequences:** 5(5) |
| --- | --- |
|  | Chain A, The X-Ray Crystallographic Structure Of The Amyloidogenic Variant Ttr Tyr78phe |

|  | [gi|62738388](http://www.matrixscience.com/cgi/protein_view.pl?file=../data/20100727/FtemCaaOR.dat&hit=gi|62738388&db_idx=1&px=1&ave_thresh=43&_ignoreionsscorebelow=0&report=5&_sigthreshold=0.05&_msresflags=1089&_msresflags2=2&percolate=-1&percolate_rt=0)    **Mass:** 13791    **Score:** 262    **Matches:** 5(5)  **Sequences:** 5(5) |
| --- | --- |
|  | Chain A, Structure Of Ttr R104h: A Non-Amyloidogenic Variant With Protective Clinical Effects |

|  | [gi|73535906](http://www.matrixscience.com/cgi/protein_view.pl?file=../data/20100727/FtemCaaOR.dat&hit=gi|73535906&db_idx=1&px=1&ave_thresh=43&_ignoreionsscorebelow=0&report=5&_sigthreshold=0.05&_msresflags=1089&_msresflags2=2&percolate=-1&percolate_rt=0)    **Mass:** 13941    **Score:** 262    **Matches:** 5(5)  **Sequences:** 5(5) |
| --- | --- |
|  | Chain A, Crystal Structure Of Human Transthyretin With Bound Iodide |

|  | [gi|114318993](http://www.matrixscience.com/cgi/protein_view.pl?file=../data/20100727/FtemCaaOR.dat&hit=gi|114318993&db_idx=1&px=1&ave_thresh=43&_ignoreionsscorebelow=0&report=5&_sigthreshold=0.05&_msresflags=1089&_msresflags2=2&percolate=-1&percolate_rt=0)    **Mass:** 20300    **Score:** 262    **Matches:** 5(5)  **Sequences:** 5(5) |
| --- | --- |
|  | transthyretin [Homo sapiens] |

|  | [gi|114319005](http://www.matrixscience.com/cgi/protein_view.pl?file=../data/20100727/FtemCaaOR.dat&hit=gi|114319005&db_idx=1&px=1&ave_thresh=43&_ignoreionsscorebelow=0&report=5&_sigthreshold=0.05&_msresflags=1089&_msresflags2=2&percolate=-1&percolate_rt=0)    **Mass:** 15062    **Score:** 262    **Matches:** 5(5)  **Sequences:** 5(5) |
| --- | --- |
|  | transthyretin [Homo sapiens] |

|  | [gi|119621670](http://www.matrixscience.com/cgi/protein_view.pl?file=../data/20100727/FtemCaaOR.dat&hit=gi|119621670&db_idx=1&px=1&ave_thresh=43&_ignoreionsscorebelow=0&report=5&_sigthreshold=0.05&_msresflags=1089&_msresflags2=2&percolate=-1&percolate_rt=0)    **Mass:** 15689    **Score:** 262    **Matches:** 5(5)  **Sequences:** 5(5) |
| --- | --- |
|  | transthyretin (prealbumin, amyloidosis type I), isoform CRA_b [Homo sapiens] |

|  | [gi|126030508](http://www.matrixscience.com/cgi/protein_view.pl?file=../data/20100727/FtemCaaOR.dat&hit=gi|126030508&db_idx=1&px=1&ave_thresh=43&_ignoreionsscorebelow=0&report=5&_sigthreshold=0.05&_msresflags=1089&_msresflags2=2&percolate=-1&percolate_rt=0)    **Mass:** 13784    **Score:** 262    **Matches:** 5(5)  **Sequences:** 5(5) |
| --- | --- |
|  | Chain A, Crystal Structure Of Transthyretin Mutant I84s At Acidic Ph |

|  | [gi|126030510](http://www.matrixscience.com/cgi/protein_view.pl?file=../data/20100727/FtemCaaOR.dat&hit=gi|126030510&db_idx=1&px=1&ave_thresh=43&_ignoreionsscorebelow=0&report=5&_sigthreshold=0.05&_msresflags=1089&_msresflags2=2&percolate=-1&percolate_rt=0)    **Mass:** 13768    **Score:** 262    **Matches:** 5(5)  **Sequences:** 5(5) |
| --- | --- |
|  | Chain A, Crystal Structure Of Transthyretin Mutant I84a At Low Ph |

|  | [gi|189339646](http://www.matrixscience.com/cgi/protein_view.pl?file=../data/20100727/FtemCaaOR.dat&hit=gi|189339646&db_idx=1&px=1&ave_thresh=43&_ignoreionsscorebelow=0&report=5&_sigthreshold=0.05&_msresflags=1089&_msresflags2=2&percolate=-1&percolate_rt=0)    **Mass:** 12740    **Score:** 262    **Matches:** 5(5)  **Sequences:** 5(5) |
| --- | --- |
|  | Chain A, Crystal Structure Of The F87mL110M MUTANT OF HUMAN Transthyretin At Ph 4.6. |

|  | [gi|212374952](http://www.matrixscience.com/cgi/protein_view.pl?file=../data/20100727/FtemCaaOR.dat&hit=gi|212374952&db_idx=1&px=1&ave_thresh=43&_ignoreionsscorebelow=0&report=5&_sigthreshold=0.05&_msresflags=1089&_msresflags2=2&percolate=-1&percolate_rt=0)    **Mass:** 13798    **Score:** 262    **Matches:** 5(5)  **Sequences:** 5(5) |
| --- | --- |
|  | Chain A, Crystal Structure Of Transthyretin Variant V20s |

|  | [gi|223674041](http://www.matrixscience.com/cgi/protein_view.pl?file=../data/20100727/FtemCaaOR.dat&hit=gi|223674041&db_idx=1&px=1&ave_thresh=43&_ignoreionsscorebelow=0&report=5&_sigthreshold=0.05&_msresflags=1089&_msresflags2=2&percolate=-1&percolate_rt=0)    **Mass:** 13456    **Score:** 262    **Matches:** 5(5)  **Sequences:** 5(5) |
| --- | --- |
|  | Chain A, Crystal Structure Of Transthyretin In Complex With Iododiflunisal-Betaalaome |

|  | [gi|224510585](http://www.matrixscience.com/cgi/protein_view.pl?file=../data/20100727/FtemCaaOR.dat&hit=gi|224510585&db_idx=1&px=1&ave_thresh=43&_ignoreionsscorebelow=0&report=5&_sigthreshold=0.05&_msresflags=1089&_msresflags2=2&percolate=-1&percolate_rt=0)    **Mass:** 12996    **Score:** 262    **Matches:** 5(5)  **Sequences:** 5(5) |
| --- | --- |
|  | Chain A, Crystal Structure Of The Apo Form Of Human Wild-Type Transthyretin |

|  | [gi|226438187](http://www.matrixscience.com/cgi/protein_view.pl?file=../data/20100727/FtemCaaOR.dat&hit=gi|226438187&db_idx=1&px=1&ave_thresh=43&_ignoreionsscorebelow=0&report=5&_sigthreshold=0.05&_msresflags=1089&_msresflags2=2&percolate=-1&percolate_rt=0)    **Mass:** 13784    **Score:** 262    **Matches:** 5(5)  **Sequences:** 5(5) |
| --- | --- |
|  | Chain A, Crystal Structure Of Transthyretin Variant Y114h |

|  | [gi|261824993](http://www.matrixscience.com/cgi/protein_view.pl?file=../data/20100727/FtemCaaOR.dat&hit=gi|261824993&db_idx=1&px=1&ave_thresh=43&_ignoreionsscorebelow=0&report=5&_sigthreshold=0.05&_msresflags=1089&_msresflags2=2&percolate=-1&percolate_rt=0)    **Mass:** 12738    **Score:** 262    **Matches:** 5(5)  **Sequences:** 5(5) |
| --- | --- |
|  | Chain A, Crystal Structure Of Human Transthyretin - Wild Type |

|  | [gi|126030594](http://www.matrixscience.com/cgi/protein_view.pl?file=../data/20100727/FtemCaaOR.dat&hit=gi|126030594&db_idx=1&px=1&ave_thresh=43&_ignoreionsscorebelow=0&report=5&_sigthreshold=0.05&_msresflags=1089&_msresflags2=2&percolate=-1&percolate_rt=0)    **Mass:** 13761    **Score:** 262    **Matches:** 5(5)  **Sequences:** 5(5) |
| --- | --- |
|  | Chain A, Crystal Structure Of Cys10 Sulfonated Transthyretin |

Bottom of Form
