# Supplementary material for: Two-dimensional difference gel electrophoresis (DIGE) analysis of sera from visceral leishmaniasis patients
Source: Clin Proteomics. 2011 May 31;8(1):4. doi: 10.1186/1559-0275-8-4 (PMC3167202; doi:10.1186/1559-0275-8-4)
Supplement: Additional file 3 — Detailed Mascot search results for identified proteins. Detailed Mascot search result showing the protein view. Score and sequence coverage for the identified protein and list of all the peptides matched is shown. [file 1559-0275-8-4-S3.DOC]

**Protein View**

**Spot 746**

Top of Form

Match to: **LPHUA1** Score: **314**

**apolipoprotein A-I precursor [validated] - human**

Found in search of C:\Temp\mas4D.tmp

Nominal mass (M_r_): **30759**; Calculated pI value: **5.56**

NCBI BLAST search of [LPHUA1](http://www.ncbi.nlm.nih.gov/blast/Blast.cgi?ALIGNMENTS=50&ALIGNMENT_VIEW=Pairwise&AUTO_FORMAT=Semiauto&CDD_SEARCH=on&CLIENT=web&COMPOSITION_BASED_STATISTICS=on&DATABASE=nr&DESCRIPTIONS=100&ENTREZ_QUERY=(none)&EXPECT=10&FILTER=L&FORMAT_BLOCK_ON_RESPAGE=None&FORMAT_OBJECT=Alignment&FORMAT_TYPE=HTML&GAPCOSTS=11+1&I_THRESH=0.001&LAYOUT=TwoWindows&MATRIX_NAME=BLOSUM62&NCBI_GI=on&PAGE=Proteins&PROGRAM=blastp&QUERY=MKAAVLTLAVLFLTGSQARHFWQQDEPPQSPWDRVKDLATVYVDVLKDSGRDYVSQFEGSALGKQLNLKLLDNWDSVTSTFSKLREQLGPVTQEFWDNLEKETEGLRQEMSKDLEEVKAKVQPYLDDFQKKWQEEMELYRQKVEPLRAELQEGARQKLHELQEKLSPLGEEMRDRARAHVDALRTHLAPYSDELRQRLAARLEALKENGGARLAEYHAKATEHLSTLSEKAKPALEDLRQGLLPVLESFKVSFLSALEEYTKKLNTQ&SERVICE=plain&SET_DEFAULTS.x=9&SET_DEFAULTS.y=5&SHOW_OVERVIEW=on&WORD_SIZE=3&END_OF_HTTPGET=Yes) against nr

Unformatted [sequence string](http://www.matrixscience.com/cgi/getseq.pl?MSDB+LPHUA1+seq) for pasting into other applications

Taxonomy: [Homo sapiens](http://www.ncbi.nlm.nih.gov/Taxonomy/Browser/wwwtax.cgi?lvl=0&id=9606)

Links to retrieve other entries containing this sequence from NCBI Entrez:

[CAA03488](http://www.ncbi.nlm.nih.gov/entrez/query.fcgi?cmd=search&db=protein&doptcmdl=genpept&tool=mascot&term=CAA03488%5Baccn%5D) from [unidentified](http://www.ncbi.nlm.nih.gov/Taxonomy/Browser/wwwtax.cgi?lvl=0&id=32644)

[E974220](http://www.ncbi.nlm.nih.gov/entrez/query.fcgi?cmd=search&db=protein&doptcmdl=genpept&tool=mascot&term=E974220%5Baccn%5D) from [Homo sapiens](http://www.ncbi.nlm.nih.gov/Taxonomy/Browser/wwwtax.cgi?lvl=0&id=9606)

[CAA01198](http://www.ncbi.nlm.nih.gov/entrez/query.fcgi?cmd=search&db=protein&doptcmdl=genpept&tool=mascot&term=CAA01198%5Baccn%5D) from [Homo sapiens](http://www.ncbi.nlm.nih.gov/Taxonomy/Browser/wwwtax.cgi?lvl=0&id=9606)

[AAQ91811](http://www.ncbi.nlm.nih.gov/entrez/query.fcgi?cmd=search&db=protein&doptcmdl=genpept&tool=mascot&term=AAQ91811%5Baccn%5D) from [Homo sapiens](http://www.ncbi.nlm.nih.gov/Taxonomy/Browser/wwwtax.cgi?lvl=0&id=9606)

[AAS68227](http://www.ncbi.nlm.nih.gov/entrez/query.fcgi?cmd=search&db=protein&doptcmdl=genpept&tool=mascot&term=AAS68227%5Baccn%5D) from [Homo sapiens](http://www.ncbi.nlm.nih.gov/Taxonomy/Browser/wwwtax.cgi?lvl=0&id=9606)

[AAH05380](http://www.ncbi.nlm.nih.gov/entrez/query.fcgi?cmd=search&db=protein&doptcmdl=genpept&tool=mascot&term=AAH05380%5Baccn%5D) from [Homo sapiens](http://www.ncbi.nlm.nih.gov/Taxonomy/Browser/wwwtax.cgi?lvl=0&id=9606)

[AAI10287](http://www.ncbi.nlm.nih.gov/entrez/query.fcgi?cmd=search&db=protein&doptcmdl=genpept&tool=mascot&term=AAI10287%5Baccn%5D) from [Homo sapiens](http://www.ncbi.nlm.nih.gov/Taxonomy/Browser/wwwtax.cgi?lvl=0&id=9606)

[CAA25232](http://www.ncbi.nlm.nih.gov/entrez/query.fcgi?cmd=search&db=protein&doptcmdl=genpept&tool=mascot&term=CAA25232%5Baccn%5D) from [Homo sapiens](http://www.ncbi.nlm.nih.gov/Taxonomy/Browser/wwwtax.cgi?lvl=0&id=9606)

[CAA25519](http://www.ncbi.nlm.nih.gov/entrez/query.fcgi?cmd=search&db=protein&doptcmdl=genpept&tool=mascot&term=CAA25519%5Baccn%5D) from [Homo sapiens](http://www.ncbi.nlm.nih.gov/Taxonomy/Browser/wwwtax.cgi?lvl=0&id=9606)

[CAA26097](http://www.ncbi.nlm.nih.gov/entrez/query.fcgi?cmd=search&db=protein&doptcmdl=genpept&tool=mascot&term=CAA26097%5Baccn%5D) from [Homo sapiens](http://www.ncbi.nlm.nih.gov/Taxonomy/Browser/wwwtax.cgi?lvl=0&id=9606)

[CAA30377](http://www.ncbi.nlm.nih.gov/entrez/query.fcgi?cmd=search&db=protein&doptcmdl=genpept&tool=mascot&term=CAA30377%5Baccn%5D) from [Homo sapiens](http://www.ncbi.nlm.nih.gov/Taxonomy/Browser/wwwtax.cgi?lvl=0&id=9606)

[AAA62829](http://www.ncbi.nlm.nih.gov/entrez/query.fcgi?cmd=search&db=protein&doptcmdl=genpept&tool=mascot&term=AAA62829%5Baccn%5D) from [Homo sapiens](http://www.ncbi.nlm.nih.gov/Taxonomy/Browser/wwwtax.cgi?lvl=0&id=9606)

[AAB59514](http://www.ncbi.nlm.nih.gov/entrez/query.fcgi?cmd=search&db=protein&doptcmdl=genpept&tool=mascot&term=AAB59514%5Baccn%5D) from [Homo sapiens](http://www.ncbi.nlm.nih.gov/Taxonomy/Browser/wwwtax.cgi?lvl=0&id=9606)

[APOA1_HUMAN](http://www.ncbi.nlm.nih.gov/entrez/query.fcgi?cmd=search&db=protein&doptcmdl=genpept&tool=mascot&term=APOA1_HUMAN%5Baccn%5D) from [Homo sapiens](http://www.ncbi.nlm.nih.gov/Taxonomy/Browser/wwwtax.cgi?lvl=0&id=9606)

Fixed modifications: Carbamidomethyl (C)

Variable modifications: Oxidation (M)

Cleavage by Trypsin: cuts C-term side of KR unless next residue is P

Sequence Coverage: **63%**

Matched peptides shown in **Bold Red**

**1** MKAAVLTLAV LFLTGSQARH FWQQDEPPQS PWDR**VKDLAT VYVDVLK**DSG

**51** R**DYVSQFEGS ALGK**QLNLK**L LDNWDSVTST FSKLREQLGP VTQEFWDNLE**

**101 K**ETEGLRQEM SKDLEEVKAK **VQPYLDDFQK KWQEEMELYR QKVEPLRAEL**

**151 QEGAR**QK**LHE LQEKLSPLGE EMR**DRARAHV DALR**THLAPY SDELR**QRLAA

**201** RLEALKENGG AR**LAEYHAKA TEHLSTLSEK AKPALEDLRQ GLLPVLESFK**

**251 VSFLSALEEY TK**KLNTQ

  Residue Number  Increasing Mass  Decreasing Mass

**Start - End Observed Mr(expt) Mr(calc) Delta Miss Sequence**

**35 - 47 488.2864 1461.8374 1461.8443 -0.0068 1 R.VKDLATVYVDVLK.D**  ([Ions score 36](http://www.matrixscience.com/cgi/peptide_view.pl?file=../data/20080404/FtgoSrETt.dat&query=123&hit=1&index=LPHUA1&px=1&section=5&ave_thresh=40))

**37 - 47 618.3441 1234.6736 1234.6809 -0.0073 0 K.DLATVYVDVLK.D**  ([Ions score 47](http://www.matrixscience.com/cgi/peptide_view.pl?file=../data/20080404/FtgoSrETt.dat&query=75&hit=1&index=LPHUA1&px=1&section=5&ave_thresh=40))

**52 - 64 467.2090 1398.6052 1399.6620 -1.0567 0 R.DYVSQFEGSALGK.Q**  ([Ions score 15](http://www.matrixscience.com/cgi/peptide_view.pl?file=../data/20080404/FtgoSrETt.dat&query=113&hit=1&index=LPHUA1&px=1&section=5&ave_thresh=40))

**52 - 64 700.8403 1399.6661 1399.6620 0.0042 0 R.DYVSQFEGSALGK.Q**  ([Ions score 47](http://www.matrixscience.com/cgi/peptide_view.pl?file=../data/20080404/FtgoSrETt.dat&query=114&hit=1&index=LPHUA1&px=1&section=5&ave_thresh=40))

**70 - 83 806.8888 1611.7630 1611.7781 -0.0150 0 K.LLDNWDSVTSTFSK.L**  ([Ions score 85](http://www.matrixscience.com/cgi/peptide_view.pl?file=../data/20080404/FtgoSrETt.dat&query=133&hit=1&index=LPHUA1&px=1&section=5&ave_thresh=40))

**84 - 101 734.6992 2201.0757 2201.1117 -0.0359 1 K.LREQLGPVTQEFWDNLEK.E**  ([Ions score 44](http://www.matrixscience.com/cgi/peptide_view.pl?file=../data/20080404/FtgoSrETt.dat&query=152&hit=1&index=LPHUA1&px=1&section=5&ave_thresh=40))

**84 - 101 735.0296 2202.0668 2201.1117 0.9552 1 K.LREQLGPVTQEFWDNLEK.E**  ([Ions score 32](http://www.matrixscience.com/cgi/peptide_view.pl?file=../data/20080404/FtgoSrETt.dat&query=153&hit=1&index=LPHUA1&px=1&section=5&ave_thresh=40))

**86 - 101 645.3143 1932.9211 1931.9265 0.9946 0 R.EQLGPVTQEFWDNLEK.E**  ([Ions score 1](http://www.matrixscience.com/cgi/peptide_view.pl?file=../data/20080404/FtgoSrETt.dat&query=143&hit=4&index=LPHUA1&px=1&section=5&ave_thresh=40))

**121 - 131 460.9002 1379.6788 1379.7085 -0.0298 1 K.VQPYLDDFQKK.W**  ([Ions score 11](http://www.matrixscience.com/cgi/peptide_view.pl?file=../data/20080404/FtgoSrETt.dat&query=109&hit=1&index=LPHUA1&px=1&section=5&ave_thresh=40))

**121 - 131 461.2444 1380.7114 1379.7085 1.0029 1 K.VQPYLDDFQKK.W**  ([Ions score 14](http://www.matrixscience.com/cgi/peptide_view.pl?file=../data/20080404/FtgoSrETt.dat&query=110&hit=1&index=LPHUA1&px=1&section=5&ave_thresh=40))

**131 - 140 471.2562 1410.7469 1410.6601 0.0867 1 K.KWQEEMELYR.Q**  ([Ions score 19](http://www.matrixscience.com/cgi/peptide_view.pl?file=../data/20080404/FtgoSrETt.dat&query=117&hit=1&index=LPHUA1&px=1&section=5&ave_thresh=40))

**131 - 140 476.5541 1426.6406 1426.6551 -0.0145 1 K.KWQEEMELYR.Q**  Oxidation (M) ([Ions score 8](http://www.matrixscience.com/cgi/peptide_view.pl?file=../data/20080404/FtgoSrETt.dat&query=119&hit=5&index=LPHUA1&px=1&section=5&ave_thresh=40))

**132 - 140 642.2958 1282.5770 1282.5652 0.0118 0 K.WQEEMELYR.Q**  ([Ions score 34](http://www.matrixscience.com/cgi/peptide_view.pl?file=../data/20080404/FtgoSrETt.dat&query=85&hit=1&index=LPHUA1&px=1&section=5&ave_thresh=40))

**132 - 140 650.2916 1298.5686 1298.5601 0.0085 0 K.WQEEMELYR.Q**  Oxidation (M) ([Ions score 7](http://www.matrixscience.com/cgi/peptide_view.pl?file=../data/20080404/FtgoSrETt.dat&query=91&hit=1&index=LPHUA1&px=1&section=5&ave_thresh=40))

**141 - 147 435.2465 868.4784 868.5130 -0.0347 1 R.QKVEPLR.A**  ([Ions score 15](http://www.matrixscience.com/cgi/peptide_view.pl?file=../data/20080404/FtgoSrETt.dat&query=21&hit=1&index=LPHUA1&px=1&section=5&ave_thresh=40))

**148 - 155 437.2162 872.4179 872.4352 -0.0173 0 R.AELQEGAR.Q**  ([Ions score 48](http://www.matrixscience.com/cgi/peptide_view.pl?file=../data/20080404/FtgoSrETt.dat&query=22&hit=1&index=LPHUA1&px=1&section=5&ave_thresh=40))

**158 - 164 448.2365 894.4585 895.4763 -1.0178 0 K.LHELQEK.L**  ([Ions score 19](http://www.matrixscience.com/cgi/peptide_view.pl?file=../data/20080404/FtgoSrETt.dat&query=25&hit=1&index=LPHUA1&px=1&section=5&ave_thresh=40))

**158 - 164 448.7388 895.4631 895.4763 -0.0132 0 K.LHELQEK.L**  ([Ions score 27](http://www.matrixscience.com/cgi/peptide_view.pl?file=../data/20080404/FtgoSrETt.dat&query=26&hit=1&index=LPHUA1&px=1&section=5&ave_thresh=40))

**165 - 173 516.2606 1030.5067 1030.5117 -0.0050 0 K.LSPLGEEMR.D**  ([Ions score 50](http://www.matrixscience.com/cgi/peptide_view.pl?file=../data/20080404/FtgoSrETt.dat&query=47&hit=1&index=LPHUA1&px=1&section=5&ave_thresh=40))

**165 - 173 524.7558 1047.4971 1046.5066 0.9905 0 K.LSPLGEEMR.D**  Oxidation (M) ([Ions score 33](http://www.matrixscience.com/cgi/peptide_view.pl?file=../data/20080404/FtgoSrETt.dat&query=53&hit=1&index=LPHUA1&px=1&section=5&ave_thresh=40))

**185 - 195 434.2027 1299.5863 1300.6411 -1.0549 0 R.THLAPYSDELR.Q**  ([Ions score 37](http://www.matrixscience.com/cgi/peptide_view.pl?file=../data/20080404/FtgoSrETt.dat&query=92&hit=1&index=LPHUA1&px=1&section=5&ave_thresh=40))

**185 - 195 434.5505 1300.6296 1300.6411 -0.0115 0 R.THLAPYSDELR.Q**  ([Ions score 34](http://www.matrixscience.com/cgi/peptide_view.pl?file=../data/20080404/FtgoSrETt.dat&query=93&hit=1&index=LPHUA1&px=1&section=5&ave_thresh=40))

**213 - 219 416.2180 830.4215 830.4286 -0.0072 0 R.LAEYHAK.A**  ([Ions score 23](http://www.matrixscience.com/cgi/peptide_view.pl?file=../data/20080404/FtgoSrETt.dat&query=11&hit=1&index=LPHUA1&px=1&section=5&ave_thresh=40))

**220 - 230 607.8360 1213.6574 1214.6143 -0.9568 0 K.ATEHLSTLSEK.A**  ([Ions score 40](http://www.matrixscience.com/cgi/peptide_view.pl?file=../data/20080404/FtgoSrETt.dat&query=69&hit=1&index=LPHUA1&px=1&section=5&ave_thresh=40))

**220 - 230 405.8749 1214.6029 1214.6143 -0.0114 0 K.ATEHLSTLSEK.A**  ([Ions score 36](http://www.matrixscience.com/cgi/peptide_view.pl?file=../data/20080404/FtgoSrETt.dat&query=70&hit=1&index=LPHUA1&px=1&section=5&ave_thresh=40))

**231 - 239 506.7874 1011.5602 1011.5713 -0.0110 0 K.AKPALEDLR.Q**  ([Ions score 34](http://www.matrixscience.com/cgi/peptide_view.pl?file=../data/20080404/FtgoSrETt.dat&query=44&hit=1&index=LPHUA1&px=1&section=5&ave_thresh=40))

**240 - 250 615.8476 1229.6807 1229.7020 -0.0213 0 R.QGLLPVLESFK.V**  ([Ions score 50](http://www.matrixscience.com/cgi/peptide_view.pl?file=../data/20080404/FtgoSrETt.dat&query=73&hit=1&index=LPHUA1&px=1&section=5&ave_thresh=40))

**251 - 262 693.8542 1385.6939 1385.7078 -0.0139 0 K.VSFLSALEEYTK.K**  ([Ions score 68](http://www.matrixscience.com/cgi/peptide_view.pl?file=../data/20080404/FtgoSrETt.dat&query=111&hit=1&index=LPHUA1&px=1&section=5&ave_thresh=40))

Bottom of Form

**Protein View**

**Spot 673**

Top of Form

Match to: **LPHUA1** Score: **114**

**apolipoprotein A-I precursor [validated] - human**

Found in search of C:\Temp\mas15.tmp

Nominal mass (M_r_): **30759**; Calculated pI value: **5.56**

NCBI BLAST search of [LPHUA1](http://www.ncbi.nlm.nih.gov/blast/Blast.cgi?ALIGNMENTS=50&ALIGNMENT_VIEW=Pairwise&AUTO_FORMAT=Semiauto&CDD_SEARCH=on&CLIENT=web&COMPOSITION_BASED_STATISTICS=on&DATABASE=nr&DESCRIPTIONS=100&ENTREZ_QUERY=(none)&EXPECT=10&FILTER=L&FORMAT_BLOCK_ON_RESPAGE=None&FORMAT_OBJECT=Alignment&FORMAT_TYPE=HTML&GAPCOSTS=11+1&I_THRESH=0.001&LAYOUT=TwoWindows&MATRIX_NAME=BLOSUM62&NCBI_GI=on&PAGE=Proteins&PROGRAM=blastp&QUERY=MKAAVLTLAVLFLTGSQARHFWQQDEPPQSPWDRVKDLATVYVDVLKDSGRDYVSQFEGSALGKQLNLKLLDNWDSVTSTFSKLREQLGPVTQEFWDNLEKETEGLRQEMSKDLEEVKAKVQPYLDDFQKKWQEEMELYRQKVEPLRAELQEGARQKLHELQEKLSPLGEEMRDRARAHVDALRTHLAPYSDELRQRLAARLEALKENGGARLAEYHAKATEHLSTLSEKAKPALEDLRQGLLPVLESFKVSFLSALEEYTKKLNTQ&SERVICE=plain&SET_DEFAULTS.x=9&SET_DEFAULTS.y=5&SHOW_OVERVIEW=on&WORD_SIZE=3&END_OF_HTTPGET=Yes) against nr

Unformatted [sequence string](http://www.matrixscience.com/cgi/getseq.pl?MSDB+LPHUA1+seq) for pasting into other applications

Taxonomy: [Homo sapiens](http://www.ncbi.nlm.nih.gov/Taxonomy/Browser/wwwtax.cgi?lvl=0&id=9606)

Links to retrieve other entries containing this sequence from NCBI Entrez:

[CAA03488](http://www.ncbi.nlm.nih.gov/entrez/query.fcgi?cmd=search&db=protein&doptcmdl=genpept&tool=mascot&term=CAA03488%5Baccn%5D) from [unidentified](http://www.ncbi.nlm.nih.gov/Taxonomy/Browser/wwwtax.cgi?lvl=0&id=32644)

[E974220](http://www.ncbi.nlm.nih.gov/entrez/query.fcgi?cmd=search&db=protein&doptcmdl=genpept&tool=mascot&term=E974220%5Baccn%5D) from [Homo sapiens](http://www.ncbi.nlm.nih.gov/Taxonomy/Browser/wwwtax.cgi?lvl=0&id=9606)

[CAA01198](http://www.ncbi.nlm.nih.gov/entrez/query.fcgi?cmd=search&db=protein&doptcmdl=genpept&tool=mascot&term=CAA01198%5Baccn%5D) from [Homo sapiens](http://www.ncbi.nlm.nih.gov/Taxonomy/Browser/wwwtax.cgi?lvl=0&id=9606)

[AAQ91811](http://www.ncbi.nlm.nih.gov/entrez/query.fcgi?cmd=search&db=protein&doptcmdl=genpept&tool=mascot&term=AAQ91811%5Baccn%5D) from [Homo sapiens](http://www.ncbi.nlm.nih.gov/Taxonomy/Browser/wwwtax.cgi?lvl=0&id=9606)

[AAS68227](http://www.ncbi.nlm.nih.gov/entrez/query.fcgi?cmd=search&db=protein&doptcmdl=genpept&tool=mascot&term=AAS68227%5Baccn%5D) from [Homo sapiens](http://www.ncbi.nlm.nih.gov/Taxonomy/Browser/wwwtax.cgi?lvl=0&id=9606)

[AAH05380](http://www.ncbi.nlm.nih.gov/entrez/query.fcgi?cmd=search&db=protein&doptcmdl=genpept&tool=mascot&term=AAH05380%5Baccn%5D) from [Homo sapiens](http://www.ncbi.nlm.nih.gov/Taxonomy/Browser/wwwtax.cgi?lvl=0&id=9606)

[AAI10287](http://www.ncbi.nlm.nih.gov/entrez/query.fcgi?cmd=search&db=protein&doptcmdl=genpept&tool=mascot&term=AAI10287%5Baccn%5D) from [Homo sapiens](http://www.ncbi.nlm.nih.gov/Taxonomy/Browser/wwwtax.cgi?lvl=0&id=9606)

[CAA25232](http://www.ncbi.nlm.nih.gov/entrez/query.fcgi?cmd=search&db=protein&doptcmdl=genpept&tool=mascot&term=CAA25232%5Baccn%5D) from [Homo sapiens](http://www.ncbi.nlm.nih.gov/Taxonomy/Browser/wwwtax.cgi?lvl=0&id=9606)

[CAA25519](http://www.ncbi.nlm.nih.gov/entrez/query.fcgi?cmd=search&db=protein&doptcmdl=genpept&tool=mascot&term=CAA25519%5Baccn%5D) from [Homo sapiens](http://www.ncbi.nlm.nih.gov/Taxonomy/Browser/wwwtax.cgi?lvl=0&id=9606)

[CAA26097](http://www.ncbi.nlm.nih.gov/entrez/query.fcgi?cmd=search&db=protein&doptcmdl=genpept&tool=mascot&term=CAA26097%5Baccn%5D) from [Homo sapiens](http://www.ncbi.nlm.nih.gov/Taxonomy/Browser/wwwtax.cgi?lvl=0&id=9606)

[CAA30377](http://www.ncbi.nlm.nih.gov/entrez/query.fcgi?cmd=search&db=protein&doptcmdl=genpept&tool=mascot&term=CAA30377%5Baccn%5D) from [Homo sapiens](http://www.ncbi.nlm.nih.gov/Taxonomy/Browser/wwwtax.cgi?lvl=0&id=9606)

[AAA62829](http://www.ncbi.nlm.nih.gov/entrez/query.fcgi?cmd=search&db=protein&doptcmdl=genpept&tool=mascot&term=AAA62829%5Baccn%5D) from [Homo sapiens](http://www.ncbi.nlm.nih.gov/Taxonomy/Browser/wwwtax.cgi?lvl=0&id=9606)

[AAB59514](http://www.ncbi.nlm.nih.gov/entrez/query.fcgi?cmd=search&db=protein&doptcmdl=genpept&tool=mascot&term=AAB59514%5Baccn%5D) from [Homo sapiens](http://www.ncbi.nlm.nih.gov/Taxonomy/Browser/wwwtax.cgi?lvl=0&id=9606)

[APOA1_HUMAN](http://www.ncbi.nlm.nih.gov/entrez/query.fcgi?cmd=search&db=protein&doptcmdl=genpept&tool=mascot&term=APOA1_HUMAN%5Baccn%5D) from [Homo sapiens](http://www.ncbi.nlm.nih.gov/Taxonomy/Browser/wwwtax.cgi?lvl=0&id=9606)

Fixed modifications: Carbamidomethyl (C)

Variable modifications: Oxidation (M)

Cleavage by Trypsin: cuts C-term side of KR unless next residue is P

Sequence Coverage: **27%**

Matched peptides shown in **Bold Red**

**1** MKAAVLTLAV LFLTGSQARH FWQQDEPPQS PWDRVKDLAT VYVDVLKDSG

**51** R**DYVSQFEGS ALGK**QLNLK**L LDNWDSVTST FSK**LREQLGP VTQEFWDNLE

**101** KETEGLRQEM SKDLEEVKAK **VQPYLDDFQK** K**WQEEMELYR** QKVEPLR**AEL**

**151 QEGAR**QKLHE LQEK**LSPLGE EMR**DRARAHV DALR**THLAPY SDELR**QRLAA

**201** RLEALKENGG ARLAEYHAKA TEHLSTLSEK AKPALEDLRQ GLLPVLESFK

**251** VSFLSALEEY TKKLNTQ

  Residue Number  Increasing Mass  Decreasing Mass

**Start - End Observed Mr(expt) Mr(calc) Delta Miss Sequence**

**52 - 64 701.3393 1400.6641 1399.6620 1.0022 0 R.DYVSQFEGSALGK.Q**  ([Ions score 12](http://www.matrixscience.com/cgi/peptide_view.pl?file=../data/20080417/FtgmriYOE.dat&query=36&hit=2&index=LPHUA1&px=1&section=5&ave_thresh=40))

**70 - 83 807.3853 1612.7560 1611.7781 0.9780 0 K.LLDNWDSVTSTFSK.L**  ([Ions score 18](http://www.matrixscience.com/cgi/peptide_view.pl?file=../data/20080417/FtgmriYOE.dat&query=38&hit=1&index=LPHUA1&px=1&section=5&ave_thresh=40))

**121 - 130 626.8052 1251.5958 1251.6136 -0.0177 0 K.VQPYLDDFQK.K**  ([Ions score 22](http://www.matrixscience.com/cgi/peptide_view.pl?file=../data/20080417/FtgmriYOE.dat&query=25&hit=1&index=LPHUA1&px=1&section=5&ave_thresh=40))

**132 - 140 642.3076 1282.6007 1282.5652 0.0355 0 K.WQEEMELYR.Q**  ([Ions score 8](http://www.matrixscience.com/cgi/peptide_view.pl?file=../data/20080417/FtgmriYOE.dat&query=27&hit=2&index=LPHUA1&px=1&section=5&ave_thresh=40))

**148 - 155 437.2117 872.4088 872.4352 -0.0263 0 R.AELQEGAR.Q**  ([Ions score 16](http://www.matrixscience.com/cgi/peptide_view.pl?file=../data/20080417/FtgmriYOE.dat&query=9&hit=2&index=LPHUA1&px=1&section=5&ave_thresh=40))

**165 - 173 516.2865 1030.5585 1030.5117 0.0468 0 K.LSPLGEEMR.D**  ([Ions score 15](http://www.matrixscience.com/cgi/peptide_view.pl?file=../data/20080417/FtgmriYOE.dat&query=17&hit=1&index=LPHUA1&px=1&section=5&ave_thresh=40))

**165 - 173 524.3222 1046.6298 1046.5066 0.1232 0 K.LSPLGEEMR.D**  Oxidation (M) ([Ions score 15](http://www.matrixscience.com/cgi/peptide_view.pl?file=../data/20080417/FtgmriYOE.dat&query=20&hit=1&index=LPHUA1&px=1&section=5&ave_thresh=40))

**185 - 195 434.8881 1301.6425 1300.6411 1.0014 0 R.THLAPYSDELR.Q**  ([Ions score 24](http://www.matrixscience.com/cgi/peptide_view.pl?file=../data/20080417/FtgmriYOE.dat&query=29&hit=1&index=LPHUA1&px=1&section=5&ave_thresh=40))

Bottom of Form

**Protein View**

**Spot 816**

Top of Form

Match to: **1QABE** Score: **110**

**retinol binding protein, chain E - human**

Found in search of C:\Temp\masA.tmp

Nominal mass (M_r_): **20745**; Calculated pI value: **4.94**

NCBI BLAST search of [1QABE](http://www.ncbi.nlm.nih.gov/blast/Blast.cgi?ALIGNMENTS=50&ALIGNMENT_VIEW=Pairwise&AUTO_FORMAT=Semiauto&CDD_SEARCH=on&CLIENT=web&COMPOSITION_BASED_STATISTICS=on&DATABASE=nr&DESCRIPTIONS=100&ENTREZ_QUERY=(none)&EXPECT=10&FILTER=L&FORMAT_BLOCK_ON_RESPAGE=None&FORMAT_OBJECT=Alignment&FORMAT_TYPE=HTML&GAPCOSTS=11+1&I_THRESH=0.001&LAYOUT=TwoWindows&MATRIX_NAME=BLOSUM62&NCBI_GI=on&PAGE=Proteins&PROGRAM=blastp&QUERY=CAVSSFRVKENFDKARFSGTWYAMAKKDPEGLFLQDNIVAEFSVDETGQMSATAKGRVRLLNNWDVCADMVGTFTDTEDPAKFKMKYWGVASFLQKGNDDHWIVDTDYDTYAVQYSCRLLNLDGTCADSYSFVFSRDPNGLPPEAQKIVAQRQEELCLAAQYRLIVHNGYCDGRSERNLL&SERVICE=plain&SET_DEFAULTS.x=9&SET_DEFAULTS.y=5&SHOW_OVERVIEW=on&WORD_SIZE=3&END_OF_HTTPGET=Yes) against nr

Unformatted [sequence string](http://www.matrixscience.com/cgi/getseq.pl?MSDB+1QABE+seq) for pasting into other applications

Taxonomy: [Homo sapiens](http://www.ncbi.nlm.nih.gov/Taxonomy/Browser/wwwtax.cgi?lvl=0&id=9606)

Fixed modifications: Carbamidomethyl (C)

Variable modifications: Oxidation (M)

Cleavage by Trypsin: cuts C-term side of KR unless next residue is P

Sequence Coverage: **27%**

Matched peptides shown in **Bold Red**

**1** CAVSSFRVKE NFDKAR**FSGT WYAMAK**KDPE GLFLQDNIVA EFSVDETGQM

**51** SATAKGRVRL LNNWDVCADM VGTFTDTEDP AKFKMK**YWGV ASFLQK**GNDD

**101** HWIVDTDYDT YAVQYSCR**LL NLDGTCADSY SFVFSR**DPNG LPPEAQKIVA

**151** QRQEELCLAA QYR**LIVHNGY CDGR**SERNLL

  Residue Number  Increasing Mass  Decreasing Mass

**Start - End Observed Mr(expt) Mr(calc) Delta Miss Sequence**

**17 - 26 581.2623 1160.5100 1160.5325 -0.0225 0 R.FSGTWYAMAK.K**  ([Ions score 23](http://www.matrixscience.com/cgi/peptide_view.pl?file=../data/20080411/FtgoCnumR.dat&query=140&hit=1&index=1QABE&px=1&section=5&ave_thresh=40&_ignoreionsscorebelow=0&report=&_sigthreshold=0.05&_msresflags=&_msresflags2=&percolate=&percolate_rt=))

**87 - 96 599.8079 1197.6013 1197.6182 -0.0169 0 K.YWGVASFLQK.G**  ([Ions score 31](http://www.matrixscience.com/cgi/peptide_view.pl?file=../data/20080411/FtgoCnumR.dat&query=145&hit=1&index=1QABE&px=1&section=5&ave_thresh=40&_ignoreionsscorebelow=0&report=&_sigthreshold=0.05&_msresflags=&_msresflags2=&percolate=&percolate_rt=))

**119 - 136 688.9734 2063.8985 2063.9623 -0.0638 0 R.LLNLDGTCADSYSFVFSR.D**  ([Ions score 19](http://www.matrixscience.com/cgi/peptide_view.pl?file=../data/20080411/FtgoCnumR.dat&query=229&hit=1&index=1QABE&px=1&section=5&ave_thresh=40&_ignoreionsscorebelow=0&report=&_sigthreshold=0.05&_msresflags=&_msresflags2=&percolate=&percolate_rt=))

**164 - 174 434.8925 1301.6558 1302.6139 -0.9581 0 R.LIVHNGYCDGR.S**  ([Ions score 36](http://www.matrixscience.com/cgi/peptide_view.pl?file=../data/20080411/FtgoCnumR.dat&query=168&hit=1&index=1QABE&px=1&section=5&ave_thresh=40&_ignoreionsscorebelow=0&report=&_sigthreshold=0.05&_msresflags=&_msresflags2=&percolate=&percolate_rt=))

**164 - 174 435.2249 1302.6530 1302.6139 0.0391 0 R.LIVHNGYCDGR.S**  ([Ions score 29](http://www.matrixscience.com/cgi/peptide_view.pl?file=../data/20080411/FtgoCnumR.dat&query=169&hit=1&index=1QABE&px=1&section=5&ave_thresh=40&_ignoreionsscorebelow=0&report=&_sigthreshold=0.05&_msresflags=&_msresflags2=&percolate=&percolate_rt=))

**164 - 174 435.5379 1303.5919 1302.6139 0.9780 0 R.LIVHNGYCDGR.S**  ([Ions score 18](http://www.matrixscience.com/cgi/peptide_view.pl?file=../data/20080411/FtgoCnumR.dat&query=171&hit=1&index=1QABE&px=1&section=5&ave_thresh=40&_ignoreionsscorebelow=0&report=&_sigthreshold=0.05&_msresflags=&_msresflags2=&percolate=&percolate_rt=))

Bottom of Form

**Protein View**

**Spot 129**

Top of Form

Match to: **ITHUC1** Score: **190**

**complement C1 inhibitor precursor [validated] - human**

Found in search of C:\Temp\mas58.tmp

Nominal mass (M_r_): **55347**; Calculated pI value: **6.09**

NCBI BLAST search of [ITHUC1](http://www.ncbi.nlm.nih.gov/blast/Blast.cgi?ALIGNMENTS=50&ALIGNMENT_VIEW=Pairwise&AUTO_FORMAT=Semiauto&CDD_SEARCH=on&CLIENT=web&COMPOSITION_BASED_STATISTICS=on&DATABASE=nr&DESCRIPTIONS=100&ENTREZ_QUERY=(none)&EXPECT=10&FILTER=L&FORMAT_BLOCK_ON_RESPAGE=None&FORMAT_OBJECT=Alignment&FORMAT_TYPE=HTML&GAPCOSTS=11+1&I_THRESH=0.001&LAYOUT=TwoWindows&MATRIX_NAME=BLOSUM62&NCBI_GI=on&PAGE=Proteins&PROGRAM=blastp&QUERY=MASRLTLLTLLLLLLAGDRASSNPNATSSSSQDPESLQDRGEGKVATTVISKMLFVEPILEVSSLPTTNSTTNSATKITANTTDEPTTQPTTEPTTQPTIQPTQPTTQLPTDSPTQPTTGSFCPGPVTLCSDLESHSTEAVLGDALVDFSLKLYHAFSAMKKVETNMAFSPFSIASLLTQVLLGAGENTKTNLESILSYPKDFTCVHQALKGFTTKGVTSVSQIFHSPDLAIRDTFVNASRTLYSSSPRVLSNNSDANLELINTWVAKNTNNKISRLLDSLPSDTRLVLLNAIYLSAKWKTTFDPKKTRMEPFHFKNSVIKVPMMNSKKYPVAHFIDQTLKAKVGQLQLSHNLSLVILVPQNLKHRLEDMEQALSPSVFKAIMEKLEMSKFQPTLLTLPRIKVTTSQDMLSIMEKLEFFDFSYDLNLCGLTEDPDLQVSAMQHQTVLELTETGVEAAAASAISVARTLLVFEVQQPFLFVLWDQQHKFPVFMGRVYDPRA&SERVICE=plain&SET_DEFAULTS.x=9&SET_DEFAULTS.y=5&SHOW_OVERVIEW=on&WORD_SIZE=3&END_OF_HTTPGET=Yes) against nr

Unformatted [sequence string](http://www.matrixscience.com/cgi/getseq.pl?MSDB+ITHUC1+seq) for pasting into other applications

Taxonomy: [Homo sapiens](http://www.ncbi.nlm.nih.gov/Taxonomy/Browser/wwwtax.cgi?lvl=0&id=9606)

Links to retrieve other entries containing this sequence from NCBI Entrez:

[Q547W3_HUMAN](http://www.ncbi.nlm.nih.gov/entrez/query.fcgi?cmd=search&db=protein&doptcmdl=genpept&tool=mascot&term=Q547W3_HUMAN%5Baccn%5D) from [Homo sapiens](http://www.ncbi.nlm.nih.gov/Taxonomy/Browser/wwwtax.cgi?lvl=0&id=9606)

[AAM21515](http://www.ncbi.nlm.nih.gov/entrez/query.fcgi?cmd=search&db=protein&doptcmdl=genpept&tool=mascot&term=AAM21515%5Baccn%5D) from [Homo sapiens](http://www.ncbi.nlm.nih.gov/Taxonomy/Browser/wwwtax.cgi?lvl=0&id=9606)

[AAW69393](http://www.ncbi.nlm.nih.gov/entrez/query.fcgi?cmd=search&db=protein&doptcmdl=genpept&tool=mascot&term=AAW69393%5Baccn%5D) from [Homo sapiens](http://www.ncbi.nlm.nih.gov/Taxonomy/Browser/wwwtax.cgi?lvl=0&id=9606)

[CAA30314](http://www.ncbi.nlm.nih.gov/entrez/query.fcgi?cmd=search&db=protein&doptcmdl=genpept&tool=mascot&term=CAA30314%5Baccn%5D) from [Homo sapiens](http://www.ncbi.nlm.nih.gov/Taxonomy/Browser/wwwtax.cgi?lvl=0&id=9606)

[CAA38358](http://www.ncbi.nlm.nih.gov/entrez/query.fcgi?cmd=search&db=protein&doptcmdl=genpept&tool=mascot&term=CAA38358%5Baccn%5D) from [Homo sapiens](http://www.ncbi.nlm.nih.gov/Taxonomy/Browser/wwwtax.cgi?lvl=0&id=9606)

[IC1_HUMAN](http://www.ncbi.nlm.nih.gov/entrez/query.fcgi?cmd=search&db=protein&doptcmdl=genpept&tool=mascot&term=IC1_HUMAN%5Baccn%5D) from [Homo sapiens](http://www.ncbi.nlm.nih.gov/Taxonomy/Browser/wwwtax.cgi?lvl=0&id=9606)

Fixed modifications: Carbamidomethyl (C)

Variable modifications: Oxidation (M)

Cleavage by Trypsin: cuts C-term side of KR unless next residue is P

Sequence Coverage: **32%**

Matched peptides shown in **Bold Red**

**1** MASRLTLLTL LLLLLAGDRA SSNPNATSSS SQDPESLQDR GEGKVATTVI

**51** SKMLFVEPIL EVSSLPTTNS TTNSATKITA NTTDEPTTQP TTEPTTQPTI

**101** QPTQPTTQLP TDSPTQPTTG SFCPGPVTLC SDLESHSTEA VLGDALVDFS

**151** LK**LYHAFSAM K**KVETNMAFS PFSIASLLTQ VLLGAGENTK **TNLESILSYP**

**201 KDFTCVHQAL K**GFTTK**GVTS VSQIFHSPDL AIR**DTFVNAS R**TLYSSSPR**V

**251** LSNNSDANLE LINTWVAKNT NNKISR**LLDS LPSDTRLVLL NAIYLSAK**WK

**301** TTFDPKKTR**M EPFHFK**NSVI K**VPMMNSK**K**Y PVAHFIDQTL K**AKVGQLQLS

**351** HNLSLVILVP QNLKHRLEDM EQALSPSVFK **AIMEK**LEMSK **FQPTLLTLPR**

**401 IKVTTSQDML SIMEK**LEFFD FSYDLNLCGL TEDPDLQVSA MQHQTVLELT

**451** ETGVEAAAAS AISVAR**TLLV FEVQQPFLFV LWDQQHKFPV FMGR**VYDPRA

**501**

  Residue Number  Increasing Mass  Decreasing Mass

**Start - End Observed Mr(expt) Mr(calc) Delta Miss Sequence**

**153 - 161 534.2755 1066.5365 1066.5270 0.0096 0 K.LYHAFSAMK.K**  ([Ions score 12](http://www.matrixscience.com/cgi/peptide_view.pl?file=../data/20080407/FtgoinEwE.dat&query=82&hit=1&index=ITHUC1&px=1&section=5&ave_thresh=40))

**153 - 161 534.7675 1067.5205 1066.5270 0.9936 0 K.LYHAFSAMK.K**  ([Ions score 9](http://www.matrixscience.com/cgi/peptide_view.pl?file=../data/20080407/FtgoinEwE.dat&query=83&hit=2&index=ITHUC1&px=1&section=5&ave_thresh=40))

**153 - 161 542.2970 1082.5793 1082.5219 0.0575 0 K.LYHAFSAMK.K**  Oxidation (M) ([Ions score 3](http://www.matrixscience.com/cgi/peptide_view.pl?file=../data/20080407/FtgoinEwE.dat&query=86&hit=6&index=ITHUC1&px=1&section=5&ave_thresh=40))

**191 - 201 632.8465 1263.6785 1263.6710 0.0075 0 K.TNLESILSYPK.D**  ([Ions score 75](http://www.matrixscience.com/cgi/peptide_view.pl?file=../data/20080407/FtgoinEwE.dat&query=127&hit=1&index=ITHUC1&px=1&section=5&ave_thresh=40))

**202 - 211 609.8000 1217.5854 1217.5863 -0.0009 0 K.DFTCVHQALK.G**  ([Ions score 9](http://www.matrixscience.com/cgi/peptide_view.pl?file=../data/20080407/FtgoinEwE.dat&query=116&hit=2&index=ITHUC1&px=1&section=5&ave_thresh=40))

**202 - 211 406.8762 1217.6068 1217.5863 0.0205 0 K.DFTCVHQALK.G**  ([Ions score 11](http://www.matrixscience.com/cgi/peptide_view.pl?file=../data/20080407/FtgoinEwE.dat&query=117&hit=3&index=ITHUC1&px=1&section=5&ave_thresh=40))

**202 - 211 407.2053 1218.5941 1217.5863 1.0078 0 K.DFTCVHQALK.G**  ([Ions score 18](http://www.matrixscience.com/cgi/peptide_view.pl?file=../data/20080407/FtgoinEwE.dat&query=118&hit=1&index=ITHUC1&px=1&section=5&ave_thresh=40))

**217 - 233 609.8000 1826.3782 1825.9687 0.4095 0 K.GVTSVSQIFHSPDLAIR.D**  ([Ions score 39](http://www.matrixscience.com/cgi/peptide_view.pl?file=../data/20080407/FtgoinEwE.dat&query=176&hit=1&index=ITHUC1&px=1&section=5&ave_thresh=40))

**217 - 233 610.0014 1826.9824 1825.9687 1.0138 0 K.GVTSVSQIFHSPDLAIR.D**  ([Ions score 23](http://www.matrixscience.com/cgi/peptide_view.pl?file=../data/20080407/FtgoinEwE.dat&query=177&hit=1&index=ITHUC1&px=1&section=5&ave_thresh=40))

**242 - 249 455.7361 909.4576 909.4556 0.0021 0 R.TLYSSSPR.V**  ([Ions score 25](http://www.matrixscience.com/cgi/peptide_view.pl?file=../data/20080407/FtgoinEwE.dat&query=48&hit=1&index=ITHUC1&px=1&section=5&ave_thresh=40))

**277 - 286 558.8005 1115.5864 1115.5822 0.0042 0 R.LLDSLPSDTR.L**  ([Ions score 50](http://www.matrixscience.com/cgi/peptide_view.pl?file=../data/20080407/FtgoinEwE.dat&query=91&hit=1&index=ITHUC1&px=1&section=5&ave_thresh=40))

**287 - 298 659.4127 1316.8109 1316.8067 0.0042 0 R.LVLLNAIYLSAK.W**  ([Ions score 62](http://www.matrixscience.com/cgi/peptide_view.pl?file=../data/20080407/FtgoinEwE.dat&query=139&hit=1&index=ITHUC1&px=1&section=5&ave_thresh=40))

**310 - 316 468.2266 934.4387 934.4371 0.0016 0 R.MEPFHFK.N**  ([Ions score 11](http://www.matrixscience.com/cgi/peptide_view.pl?file=../data/20080407/FtgoinEwE.dat&query=54&hit=1&index=ITHUC1&px=1&section=5&ave_thresh=40))

**322 - 328 404.1992 806.3839 805.3826 1.0012 0 K.VPMMNSK.K**  ([Ions score 16](http://www.matrixscience.com/cgi/peptide_view.pl?file=../data/20080407/FtgoinEwE.dat&query=27&hit=2&index=ITHUC1&px=1&section=5&ave_thresh=40))

**322 - 328 412.2034 822.3923 821.3775 1.0147 0 K.VPMMNSK.K**  Oxidation (M) ([Ions score 23](http://www.matrixscience.com/cgi/peptide_view.pl?file=../data/20080407/FtgoinEwE.dat&query=32&hit=1&index=ITHUC1&px=1&section=5&ave_thresh=40))

**330 - 341 478.2602 1431.7589 1430.7558 1.0031 0 K.YPVAHFIDQTLK.A**  ([Ions score 28](http://www.matrixscience.com/cgi/peptide_view.pl?file=../data/20080407/FtgoinEwE.dat&query=153&hit=1&index=ITHUC1&px=1&section=5&ave_thresh=40))

**381 - 385 591.3000 590.2927 590.3098 -0.0170 0 K.AIMEK.L**  ([Ions score 2](http://www.matrixscience.com/cgi/peptide_view.pl?file=../data/20080407/FtgoinEwE.dat&query=17&hit=6&index=ITHUC1&px=1&section=5&ave_thresh=40))

**391 - 400 593.3532 1184.6919 1184.6917 0.0002 0 K.FQPTLLTLPR.I**  ([Ions score 25](http://www.matrixscience.com/cgi/peptide_view.pl?file=../data/20080407/FtgoinEwE.dat&query=107&hit=1&index=ITHUC1&px=1&section=5&ave_thresh=40))

**401 - 415 575.3108 1722.9105 1722.8896 0.0210 1 R.IKVTTSQDMLSIMEK.L**  ([Ions score 24](http://www.matrixscience.com/cgi/peptide_view.pl?file=../data/20080407/FtgoinEwE.dat&query=169&hit=1&index=ITHUC1&px=1&section=5&ave_thresh=40))

**403 - 415 749.3602 1496.7058 1497.7055 -0.9996 0 K.VTTSQDMLSIMEK.L**  Oxidation (M) ([Ions score 38](http://www.matrixscience.com/cgi/peptide_view.pl?file=../data/20080407/FtgoinEwE.dat&query=159&hit=1&index=ITHUC1&px=1&section=5&ave_thresh=40))

**467 - 487 872.7997 2615.3772 2614.3948 0.9824 0 R.TLLVFEVQQPFLFVLWDQQHK.F**  ([Ions score 43](http://www.matrixscience.com/cgi/peptide_view.pl?file=../data/20080407/FtgoinEwE.dat&query=196&hit=1&index=ITHUC1&px=1&section=5&ave_thresh=40))

**488 - 494 427.2547 852.4948 852.4316 0.0632 0 K.FPVFMGR.V**  ([Ions score 14](http://www.matrixscience.com/cgi/peptide_view.pl?file=../data/20080407/FtgoinEwE.dat&query=36&hit=1&index=ITHUC1&px=1&section=5&ave_thresh=40))

Bottom of Form

**Protein View**

**Spot 1051**

Top of Form

Match to: **OMHU1** Score: **166**

**alpha-1-acid glycoprotein 1 precursor [validated] - human**

Found in search of C:\Temp\mas57.tmp

Nominal mass (M_r_): **23725**; Calculated pI value: **4.93**

NCBI BLAST search of [OMHU1](http://www.ncbi.nlm.nih.gov/blast/Blast.cgi?ALIGNMENTS=50&ALIGNMENT_VIEW=Pairwise&AUTO_FORMAT=Semiauto&CDD_SEARCH=on&CLIENT=web&COMPOSITION_BASED_STATISTICS=on&DATABASE=nr&DESCRIPTIONS=100&ENTREZ_QUERY=(none)&EXPECT=10&FILTER=L&FORMAT_BLOCK_ON_RESPAGE=None&FORMAT_OBJECT=Alignment&FORMAT_TYPE=HTML&GAPCOSTS=11+1&I_THRESH=0.001&LAYOUT=TwoWindows&MATRIX_NAME=BLOSUM62&NCBI_GI=on&PAGE=Proteins&PROGRAM=blastp&QUERY=MALSWVLTVLSLLPLLEAQIPLCANLVPVPITNATLDQITGKWFYIASAFRNEEYNKSVQEIQATFFYFTPNKTEDTIFLREYQTRQDQCIYNTTYLNVQRENGTISRYVGGQEHFAHLLILRDTKTYMLAFDVNDEKNWGLSVYADKPETTKEQLGEFYEALDCLRIPKSDVVYTDWKKDKCEPLEKQHEKERKQEEGES&SERVICE=plain&SET_DEFAULTS.x=9&SET_DEFAULTS.y=5&SHOW_OVERVIEW=on&WORD_SIZE=3&END_OF_HTTPGET=Yes) against nr

Unformatted [sequence string](http://www.matrixscience.com/cgi/getseq.pl?MSDB+OMHU1+seq) for pasting into other applications

Taxonomy: [Homo sapiens](http://www.ncbi.nlm.nih.gov/Taxonomy/Browser/wwwtax.cgi?lvl=0&id=9606)

Links to retrieve other entries containing this sequence from NCBI Entrez:

[Q5U067_HUMAN](http://www.ncbi.nlm.nih.gov/entrez/query.fcgi?cmd=search&db=protein&doptcmdl=genpept&tool=mascot&term=Q5U067_HUMAN%5Baccn%5D) from [Homo sapiens](http://www.ncbi.nlm.nih.gov/Taxonomy/Browser/wwwtax.cgi?lvl=0&id=9606)

[CAE93805](http://www.ncbi.nlm.nih.gov/entrez/query.fcgi?cmd=search&db=protein&doptcmdl=genpept&tool=mascot&term=CAE93805%5Baccn%5D) from [Homo sapiens](http://www.ncbi.nlm.nih.gov/Taxonomy/Browser/wwwtax.cgi?lvl=0&id=9606)

[AAV38593](http://www.ncbi.nlm.nih.gov/entrez/query.fcgi?cmd=search&db=protein&doptcmdl=genpept&tool=mascot&term=AAV38593%5Baccn%5D) from [Homo sapiens](http://www.ncbi.nlm.nih.gov/Taxonomy/Browser/wwwtax.cgi?lvl=0&id=9606)

[CAA26397](http://www.ncbi.nlm.nih.gov/entrez/query.fcgi?cmd=search&db=protein&doptcmdl=genpept&tool=mascot&term=CAA26397%5Baccn%5D) from [Homo sapiens](http://www.ncbi.nlm.nih.gov/Taxonomy/Browser/wwwtax.cgi?lvl=0&id=9606)

[AAA35515](http://www.ncbi.nlm.nih.gov/entrez/query.fcgi?cmd=search&db=protein&doptcmdl=genpept&tool=mascot&term=AAA35515%5Baccn%5D) from [Homo sapiens](http://www.ncbi.nlm.nih.gov/Taxonomy/Browser/wwwtax.cgi?lvl=0&id=9606)

[AAX41394](http://www.ncbi.nlm.nih.gov/entrez/query.fcgi?cmd=search&db=protein&doptcmdl=genpept&tool=mascot&term=AAX41394%5Baccn%5D) from [synthetic construct](http://www.ncbi.nlm.nih.gov/Taxonomy/Browser/wwwtax.cgi?lvl=0&id=32630)

[A1AG1_HUMAN](http://www.ncbi.nlm.nih.gov/entrez/query.fcgi?cmd=search&db=protein&doptcmdl=genpept&tool=mascot&term=A1AG1_HUMAN%5Baccn%5D) from [Homo sapiens](http://www.ncbi.nlm.nih.gov/Taxonomy/Browser/wwwtax.cgi?lvl=0&id=9606)

Fixed modifications: Carbamidomethyl (C)

Variable modifications: Oxidation (M)

Cleavage by Trypsin: cuts C-term side of KR unless next residue is P

Sequence Coverage: **48%**

Matched peptides shown in **Bold Red**

**1** MALSWVLTVL SLLPLLEAQI PLCANLVPVP ITNATLDQIT GK**WFYIASAF**

**51 R**NEEYNKSVQ EIQATFFYFT PNK**TEDTIFL R**EYQTRQDQC IYNTTYLNVQ

**101** RENGTISR**YV GGQEHFAHLL ILR**DTK**TYML AFDVNDEKNW GLSVYADKPE**

**151 TTKEQLGEFY EALDCLR**IPK **SDVVYTDWK**K **DKCEPLEK**QH EKER**KQEEGE**

**201 S**

  Residue Number  Increasing Mass  Decreasing Mass

**Start - End Observed Mr(expt) Mr(calc) Delta Miss Sequence**

**43 - 51 580.7968 1159.5791 1159.5815 -0.0023 0 K.WFYIASAFR.N**  ([Ions score 47](http://www.matrixscience.com/cgi/peptide_view.pl?file=../data/20080407/FtgoinTne.dat&query=80&hit=1&index=OMHU1&px=1&section=5&ave_thresh=40))

**74 - 81 497.7657 993.5168 993.5131 0.0037 0 K.TEDTIFLR.E**  ([Ions score 54](http://www.matrixscience.com/cgi/peptide_view.pl?file=../data/20080407/FtgoinTne.dat&query=49&hit=1&index=OMHU1&px=1&section=5&ave_thresh=40))

**74 - 81 497.9000 993.7854 993.5131 0.2723 0 K.TEDTIFLR.E**  ([Ions score 54](http://www.matrixscience.com/cgi/peptide_view.pl?file=../data/20080407/FtgoinTne.dat&query=50&hit=1&index=OMHU1&px=1&section=5&ave_thresh=40))

**109 - 123 439.2452 1752.9516 1751.9471 1.0045 0 R.YVGGQEHFAHLLILR.D**  ([Ions score 13](http://www.matrixscience.com/cgi/peptide_view.pl?file=../data/20080407/FtgoinTne.dat&query=139&hit=1&index=OMHU1&px=1&section=5&ave_thresh=40))

**127 - 138 723.3700 1444.7254 1444.6544 0.0710 0 K.TYMLAFDVNDEK.N**  ([Ions score 42](http://www.matrixscience.com/cgi/peptide_view.pl?file=../data/20080407/FtgoinTne.dat&query=120&hit=1&index=OMHU1&px=1&section=5&ave_thresh=40))

**127 - 138 731.3468 1460.6790 1460.6493 0.0297 0 K.TYMLAFDVNDEK.N**  Oxidation (M) ([Ions score 10](http://www.matrixscience.com/cgi/peptide_view.pl?file=../data/20080407/FtgoinTne.dat&query=122&hit=1&index=OMHU1&px=1&section=5&ave_thresh=40))

**127 - 153 784.6363 3134.5161 3134.4906 0.0255 1 K.TYMLAFDVNDEKNWGLSVYADKPETTK.E**  ([Ions score 20](http://www.matrixscience.com/cgi/peptide_view.pl?file=../data/20080407/FtgoinTne.dat&query=166&hit=1&index=OMHU1&px=1&section=5&ave_thresh=40))

**139 - 153 570.2828 1707.8265 1707.8468 -0.0203 0 K.NWGLSVYADKPETTK.E**  ([Ions score 26](http://www.matrixscience.com/cgi/peptide_view.pl?file=../data/20080407/FtgoinTne.dat&query=134&hit=1&index=OMHU1&px=1&section=5&ave_thresh=40))

**154 - 167 872.3926 1742.7707 1741.7981 0.9725 0 K.EQLGEFYEALDCLR.I**  ([Ions score 68](http://www.matrixscience.com/cgi/peptide_view.pl?file=../data/20080407/FtgoinTne.dat&query=137&hit=1&index=OMHU1&px=1&section=5&ave_thresh=40))

**171 - 179 556.7613 1111.5081 1111.5186 -0.0105 0 K.SDVVYTDWK.K**  ([Ions score 42](http://www.matrixscience.com/cgi/peptide_view.pl?file=../data/20080407/FtgoinTne.dat&query=71&hit=1&index=OMHU1&px=1&section=5&ave_thresh=40))

**181 - 188 509.2574 1016.5002 1017.4801 -0.9798 1 K.DKCEPLEK.Q**  ([Ions score 29](http://www.matrixscience.com/cgi/peptide_view.pl?file=../data/20080407/FtgoinTne.dat&query=54&hit=1&index=OMHU1&px=1&section=5&ave_thresh=40))

**181 - 188 509.7441 1017.4736 1017.4801 -0.0064 1 K.DKCEPLEK.Q**  ([Ions score 21](http://www.matrixscience.com/cgi/peptide_view.pl?file=../data/20080407/FtgoinTne.dat&query=55&hit=1&index=OMHU1&px=1&section=5&ave_thresh=40))

**195 - 201 404.2000 806.3854 805.3454 1.0401 1 R.KQEEGES.-**  ([Ions score 11](http://www.matrixscience.com/cgi/peptide_view.pl?file=../data/20080407/FtgoinTne.dat&query=19&hit=8&index=OMHU1&px=1&section=5&ave_thresh=40))

Bottom of Form

**Protein View**

**Spot 954**

Top of Form

Match to: **gi|126030594** Score: **503**

**Chain A, Crystal Structure Of Cys10 Sulfonated Transthyretin**

Found in search of C:\DOCUME~1\DRAF40~1.JIT\LOCALS~1\Temp\mas1B.tmp

Nominal mass (M_r_): **13761**; Calculated pI value: **5.35**

NCBI BLAST search of [gi|126030594](http://www.ncbi.nlm.nih.gov/blast/Blast.cgi?ALIGNMENTS=50&ALIGNMENT_VIEW=Pairwise&AUTO_FORMAT=Semiauto&CDD_SEARCH=on&CLIENT=web&COMPOSITION_BASED_STATISTICS=on&DATABASE=nr&DESCRIPTIONS=100&ENTREZ_QUERY=(none)&EXPECT=10&FILTER=L&FORMAT_BLOCK_ON_RESPAGE=None&FORMAT_OBJECT=Alignment&FORMAT_TYPE=HTML&GAPCOSTS=11+1&I_THRESH=0.001&LAYOUT=TwoWindows&MATRIX_NAME=BLOSUM62&NCBI_GI=on&PAGE=Proteins&PROGRAM=blastp&QUERY=GPTGTGESKXPLMVKVLDAVRGSPAINVAVHVFRKAADDTWEPFASGKTSESGELHGLTTEEEFVEGIYKVEIDTKSYWKALGISPFHEHAEVVFTANDSGPRRYTIAALLSPYSYSTTAVVTNPKE&SERVICE=plain&SET_DEFAULTS.x=9&SET_DEFAULTS.y=5&SHOW_OVERVIEW=on&WORD_SIZE=3&END_OF_HTTPGET=Yes) against nr

Unformatted [sequence string](http://www.matrixscience.com/cgi/getseq.pl?NCBInr+gi|126030594+seq) for pasting into other applications

Taxonomy: [Homo sapiens](http://www.ncbi.nlm.nih.gov/Taxonomy/Browser/wwwtax.cgi?lvl=0&id=9606)

Links to retrieve other entries containing this sequence from NCBI Entrez:

[gi|126030595](http://www.ncbi.nlm.nih.gov/entrez/query.fcgi?cmd=search&db=protein&doptcmdl=genpept&tool=mascot&term=126030595) from [Homo sapiens](http://www.ncbi.nlm.nih.gov/Taxonomy/Browser/wwwtax.cgi?lvl=0&id=9606)

[gi|281500772](http://www.ncbi.nlm.nih.gov/entrez/query.fcgi?cmd=search&db=protein&doptcmdl=genpept&tool=mascot&term=281500772) from [Homo sapiens](http://www.ncbi.nlm.nih.gov/Taxonomy/Browser/wwwtax.cgi?lvl=0&id=9606)

[gi|281500773](http://www.ncbi.nlm.nih.gov/entrez/query.fcgi?cmd=search&db=protein&doptcmdl=genpept&tool=mascot&term=281500773) from [Homo sapiens](http://www.ncbi.nlm.nih.gov/Taxonomy/Browser/wwwtax.cgi?lvl=0&id=9606)

Fixed modifications: Carbamidomethyl (C)

Variable modifications: Oxidation (M)

Cleavage by Trypsin: cuts C-term side of KR unless next residue is P

Sequence Coverage: **84%**

Matched peptides shown in **Bold Red**

**1** GPTGTGESK**X PLMVKVLDAV RGSPAINVAV HVFRKAADDT WEPFASGKTS**

**51 ESGELHGLTT EEEFVEGIYK** VEIDTKSYWK **ALGISPFHEH AEVVFTANDS**

**101 GPRRYTIAAL LSPYSYSTTA VVTNPK**E

  Residue Number  Increasing Mass  Decreasing Mass

**Start - End Observed Mr(expt) Mr(calc) Delta Miss Sequence**

**10 - 21 692.3883 1382.7621 1383.8272 -1.0651 1 K.KPLMVKVLDAVR.G**  Oxidation (M) ([Ions score 23](http://www.matrixscience.com/cgi/peptide_view.pl?file=../data/20100727/FtemCacte.dat&query=470&hit=1&index=gi|126030594&px=1&section=5&ave_thresh=42&_ignoreionsscorebelow=0&report=5&_sigthreshold=0.05&_msresflags=1089&_msresflags2=2&percolate=-1&percolate_rt=0))

**22 - 34 683.8951 1365.7757 1365.7517 0.0240 0 R.GSPAINVAVHVFR.K**  ([Ions score 74](http://www.matrixscience.com/cgi/peptide_view.pl?file=../data/20100727/FtemCacte.dat&query=462&hit=1&index=gi|126030594&px=1&section=5&ave_thresh=42&_ignoreionsscorebelow=0&report=5&_sigthreshold=0.05&_msresflags=1089&_msresflags2=2&percolate=-1&percolate_rt=0))

**22 - 34 684.3916 1366.7687 1365.7517 1.0170 0 R.GSPAINVAVHVFR.K**  ([Ions score 48](http://www.matrixscience.com/cgi/peptide_view.pl?file=../data/20100727/FtemCacte.dat&query=463&hit=1&index=gi|126030594&px=1&section=5&ave_thresh=42&_ignoreionsscorebelow=0&report=5&_sigthreshold=0.05&_msresflags=1089&_msresflags2=2&percolate=-1&percolate_rt=0))

**22 - 35 747.9448 1493.8750 1493.8467 0.0283 1 R.GSPAINVAVHVFRK.A**  ([Ions score 71](http://www.matrixscience.com/cgi/peptide_view.pl?file=../data/20100727/FtemCacte.dat&query=515&hit=1&index=gi|126030594&px=1&section=5&ave_thresh=42&_ignoreionsscorebelow=0&report=5&_sigthreshold=0.05&_msresflags=1089&_msresflags2=2&percolate=-1&percolate_rt=0))

**35 - 48 761.8735 1521.7324 1521.7100 0.0225 1 R.KAADDTWEPFASGK.T**  ([Ions score 131](http://www.matrixscience.com/cgi/peptide_view.pl?file=../data/20100727/FtemCacte.dat&query=524&hit=1&index=gi|126030594&px=1&section=5&ave_thresh=42&_ignoreionsscorebelow=0&report=5&_sigthreshold=0.05&_msresflags=1089&_msresflags2=2&percolate=-1&percolate_rt=0))

**36 - 48 697.8249 1393.6352 1393.6150 0.0202 0 K.AADDTWEPFASGK.T**  ([Ions score 80](http://www.matrixscience.com/cgi/peptide_view.pl?file=../data/20100727/FtemCacte.dat&query=473&hit=1&index=gi|126030594&px=1&section=5&ave_thresh=42&_ignoreionsscorebelow=0&report=5&_sigthreshold=0.05&_msresflags=1089&_msresflags2=2&percolate=-1&percolate_rt=0))

**49 - 70 819.4052 2455.1939 2454.1438 1.0501 0 K.TSESGELHGLTTEEEFVEGIYK.V**  ([Ions score 44](http://www.matrixscience.com/cgi/peptide_view.pl?file=../data/20100727/FtemCacte.dat&query=607&hit=1&index=gi|126030594&px=1&section=5&ave_thresh=42&_ignoreionsscorebelow=0&report=5&_sigthreshold=0.05&_msresflags=1089&_msresflags2=2&percolate=-1&percolate_rt=0))

**49 - 70 819.4056 2455.1951 2454.1438 1.0513 0 K.TSESGELHGLTTEEEFVEGIYK.V**  ([Ions score 47](http://www.matrixscience.com/cgi/peptide_view.pl?file=../data/20100727/FtemCacte.dat&query=608&hit=1&index=gi|126030594&px=1&section=5&ave_thresh=42&_ignoreionsscorebelow=0&report=5&_sigthreshold=0.05&_msresflags=1089&_msresflags2=2&percolate=-1&percolate_rt=0))

**49 - 70 819.4066 2455.1979 2454.1438 1.0541 0 K.TSESGELHGLTTEEEFVEGIYK.V**  ([Ions score 71](http://www.matrixscience.com/cgi/peptide_view.pl?file=../data/20100727/FtemCacte.dat&query=609&hit=1&index=gi|126030594&px=1&section=5&ave_thresh=42&_ignoreionsscorebelow=0&report=5&_sigthreshold=0.05&_msresflags=1089&_msresflags2=2&percolate=-1&percolate_rt=0))

**81 - 103 818.0922 2451.2547 2450.1979 1.0568 0 K.ALGISPFHEHAEVVFTANDSGPR.R**  ([Ions score 75](http://www.matrixscience.com/cgi/peptide_view.pl?file=../data/20100727/FtemCacte.dat&query=606&hit=2&index=gi|126030594&px=1&section=5&ave_thresh=42&_ignoreionsscorebelow=0&report=5&_sigthreshold=0.05&_msresflags=1089&_msresflags2=2&percolate=-1&percolate_rt=0))

**104 - 126 839.7968 2516.3685 2515.3322 1.0363 1 R.RYTIAALLSPYSYSTTAVVTNPK.E**  ([Ions score 84](http://www.matrixscience.com/cgi/peptide_view.pl?file=../data/20100727/FtemCacte.dat&query=618&hit=1&index=gi|126030594&px=1&section=5&ave_thresh=42&_ignoreionsscorebelow=0&report=5&_sigthreshold=0.05&_msresflags=1089&_msresflags2=2&percolate=-1&percolate_rt=0))

**105 - 126 1181.1316 2360.2487 2359.2311 1.0176 0 R.YTIAALLSPYSYSTTAVVTNPK.E**  ([Ions score 40](http://www.matrixscience.com/cgi/peptide_view.pl?file=../data/20100727/FtemCacte.dat&query=600&hit=1&index=gi|126030594&px=1&section=5&ave_thresh=42&_ignoreionsscorebelow=0&report=5&_sigthreshold=0.05&_msresflags=1089&_msresflags2=2&percolate=-1&percolate_rt=0))

Bottom of Form

**Protein View**

**Spot 958**

Top of Form

Match to: **gi|126030594** Score: **449**

**Chain A, Crystal Structure Of Cys10 Sulfonated Transthyretin**

Found in search of C:\DOCUME~1\DRAF40~1.JIT\LOCALS~1\Temp\mas18.tmp

Nominal mass (M_r_): **13761**; Calculated pI value: **5.35**

NCBI BLAST search of [gi|126030594](http://www.ncbi.nlm.nih.gov/blast/Blast.cgi?ALIGNMENTS=50&ALIGNMENT_VIEW=Pairwise&AUTO_FORMAT=Semiauto&CDD_SEARCH=on&CLIENT=web&COMPOSITION_BASED_STATISTICS=on&DATABASE=nr&DESCRIPTIONS=100&ENTREZ_QUERY=(none)&EXPECT=10&FILTER=L&FORMAT_BLOCK_ON_RESPAGE=None&FORMAT_OBJECT=Alignment&FORMAT_TYPE=HTML&GAPCOSTS=11+1&I_THRESH=0.001&LAYOUT=TwoWindows&MATRIX_NAME=BLOSUM62&NCBI_GI=on&PAGE=Proteins&PROGRAM=blastp&QUERY=GPTGTGESKXPLMVKVLDAVRGSPAINVAVHVFRKAADDTWEPFASGKTSESGELHGLTTEEEFVEGIYKVEIDTKSYWKALGISPFHEHAEVVFTANDSGPRRYTIAALLSPYSYSTTAVVTNPKE&SERVICE=plain&SET_DEFAULTS.x=9&SET_DEFAULTS.y=5&SHOW_OVERVIEW=on&WORD_SIZE=3&END_OF_HTTPGET=Yes) against nr

Unformatted [sequence string](http://www.matrixscience.com/cgi/getseq.pl?NCBInr+gi|126030594+seq) for pasting into other applications

Taxonomy: [Homo sapiens](http://www.ncbi.nlm.nih.gov/Taxonomy/Browser/wwwtax.cgi?lvl=0&id=9606)

Links to retrieve other entries containing this sequence from NCBI Entrez:

[gi|126030595](http://www.ncbi.nlm.nih.gov/entrez/query.fcgi?cmd=search&db=protein&doptcmdl=genpept&tool=mascot&term=126030595) from [Homo sapiens](http://www.ncbi.nlm.nih.gov/Taxonomy/Browser/wwwtax.cgi?lvl=0&id=9606)

[gi|281500772](http://www.ncbi.nlm.nih.gov/entrez/query.fcgi?cmd=search&db=protein&doptcmdl=genpept&tool=mascot&term=281500772) from [Homo sapiens](http://www.ncbi.nlm.nih.gov/Taxonomy/Browser/wwwtax.cgi?lvl=0&id=9606)

[gi|281500773](http://www.ncbi.nlm.nih.gov/entrez/query.fcgi?cmd=search&db=protein&doptcmdl=genpept&tool=mascot&term=281500773) from [Homo sapiens](http://www.ncbi.nlm.nih.gov/Taxonomy/Browser/wwwtax.cgi?lvl=0&id=9606)

Fixed modifications: Carbamidomethyl (C)

Variable modifications: Oxidation (M)

Cleavage by Trypsin: cuts C-term side of KR unless next residue is P

Sequence Coverage: **95%**

Matched peptides shown in **Bold Red**

**1** **GPTGTGESKX PLMVK**VLDAV R**GSPAINVAV HVFRKAADDT WEPFASGKTS**

**51 ESGELHGLTT EEEFVEGIYK VEIDTKSYWK ALGISPFHEH AEVVFTANDS**

**101 GPRRYTIAAL LSPYSYSTTA VVTNPKE**

  Residue Number  Increasing Mass  Decreasing Mass

**Start - End Observed Mr(expt) Mr(calc) Delta Miss Sequence**

**1 - 15 492.2786 1473.8140 1473.7497 0.0642 1 -.GPTGTGESKGPLMVK.V**  Oxidation (M) ([Ions score 18](http://www.matrixscience.com/cgi/peptide_view.pl?file=../data/20100727/FtemCacah.dat&query=549&hit=1&index=gi|126030594&px=1&section=5&ave_thresh=42&_ignoreionsscorebelow=0&report=5&_sigthreshold=0.05&_msresflags=1089&_msresflags2=2&percolate=-1&percolate_rt=0))

**1 - 15 526.2709 1575.7909 1576.7589 -0.9680 1 -.GPTGTGESKCPLMVK.V**  Oxidation (M) ([Ions score 32](http://www.matrixscience.com/cgi/peptide_view.pl?file=../data/20100727/FtemCacah.dat&query=594&hit=2&index=gi|126030594&px=1&section=5&ave_thresh=42&_ignoreionsscorebelow=0&report=5&_sigthreshold=0.05&_msresflags=1089&_msresflags2=2&percolate=-1&percolate_rt=0))

**22 - 34 683.8955 1365.7764 1365.7517 0.0246 0 R.GSPAINVAVHVFR.K**  ([Ions score 71](http://www.matrixscience.com/cgi/peptide_view.pl?file=../data/20100727/FtemCacah.dat&query=503&hit=1&index=gi|126030594&px=1&section=5&ave_thresh=42&_ignoreionsscorebelow=0&report=5&_sigthreshold=0.05&_msresflags=1089&_msresflags2=2&percolate=-1&percolate_rt=0))

**22 - 35 747.9383 1493.8621 1493.8467 0.0154 1 R.GSPAINVAVHVFRK.A**  ([Ions score 90](http://www.matrixscience.com/cgi/peptide_view.pl?file=../data/20100727/FtemCacah.dat&query=560&hit=1&index=gi|126030594&px=1&section=5&ave_thresh=42&_ignoreionsscorebelow=0&report=5&_sigthreshold=0.05&_msresflags=1089&_msresflags2=2&percolate=-1&percolate_rt=0))

**35 - 48 761.8779 1521.7412 1521.7100 0.0312 1 R.KAADDTWEPFASGK.T**  ([Ions score 97](http://www.matrixscience.com/cgi/peptide_view.pl?file=../data/20100727/FtemCacah.dat&query=571&hit=1&index=gi|126030594&px=1&section=5&ave_thresh=42&_ignoreionsscorebelow=0&report=5&_sigthreshold=0.05&_msresflags=1089&_msresflags2=2&percolate=-1&percolate_rt=0))

**36 - 48 697.3707 1392.7268 1393.6150 -0.8882 0 K.AADDTWEPFASGK.T**  ([Ions score 86](http://www.matrixscience.com/cgi/peptide_view.pl?file=../data/20100727/FtemCacah.dat&query=510&hit=1&index=gi|126030594&px=1&section=5&ave_thresh=42&_ignoreionsscorebelow=0&report=5&_sigthreshold=0.05&_msresflags=1089&_msresflags2=2&percolate=-1&percolate_rt=0))

**49 - 70 819.4109 2455.2109 2454.1438 1.0671 0 K.TSESGELHGLTTEEEFVEGIYK.V**  ([Ions score 68](http://www.matrixscience.com/cgi/peptide_view.pl?file=../data/20100727/FtemCacah.dat&query=664&hit=1&index=gi|126030594&px=1&section=5&ave_thresh=42&_ignoreionsscorebelow=0&report=5&_sigthreshold=0.05&_msresflags=1089&_msresflags2=2&percolate=-1&percolate_rt=0))

**49 - 70 819.4197 2455.2372 2454.1438 1.0934 0 K.TSESGELHGLTTEEEFVEGIYK.V**  ([Ions score 12](http://www.matrixscience.com/cgi/peptide_view.pl?file=../data/20100727/FtemCacah.dat&query=666&hit=1&index=gi|126030594&px=1&section=5&ave_thresh=42&_ignoreionsscorebelow=0&report=5&_sigthreshold=0.05&_msresflags=1089&_msresflags2=2&percolate=-1&percolate_rt=0))

**71 - 80 635.3548 1268.6951 1267.6448 1.0502 1 K.VEIDTKSYWK.A**  ([Ions score 51](http://www.matrixscience.com/cgi/peptide_view.pl?file=../data/20100727/FtemCacah.dat&query=451&hit=1&index=gi|126030594&px=1&section=5&ave_thresh=42&_ignoreionsscorebelow=0&report=5&_sigthreshold=0.05&_msresflags=1089&_msresflags2=2&percolate=-1&percolate_rt=0))

**81 - 103 818.1043 2451.2911 2450.1979 1.0932 0 K.ALGISPFHEHAEVVFTANDSGPR.R**  ([Ions score 85](http://www.matrixscience.com/cgi/peptide_view.pl?file=../data/20100727/FtemCacah.dat&query=662&hit=1&index=gi|126030594&px=1&section=5&ave_thresh=42&_ignoreionsscorebelow=0&report=5&_sigthreshold=0.05&_msresflags=1089&_msresflags2=2&percolate=-1&percolate_rt=0))

**104 - 126 839.7993 2516.3761 2515.3322 1.0439 1 R.RYTIAALLSPYSYSTTAVVTNPK.E**  ([Ions score 76](http://www.matrixscience.com/cgi/peptide_view.pl?file=../data/20100727/FtemCacah.dat&query=674&hit=1&index=gi|126030594&px=1&section=5&ave_thresh=42&_ignoreionsscorebelow=0&report=5&_sigthreshold=0.05&_msresflags=1089&_msresflags2=2&percolate=-1&percolate_rt=0))

**105 - 126 1181.1312 2360.2478 2359.2311 1.0167 0 R.YTIAALLSPYSYSTTAVVTNPK.E**  ([Ions score 43](http://www.matrixscience.com/cgi/peptide_view.pl?file=../data/20100727/FtemCacah.dat&query=654&hit=1&index=gi|126030594&px=1&section=5&ave_thresh=42&_ignoreionsscorebelow=0&report=5&_sigthreshold=0.05&_msresflags=1089&_msresflags2=2&percolate=-1&percolate_rt=0))

**105 - 127 830.7868 2489.3386 2488.2737 1.0649 1 R.YTIAALLSPYSYSTTAVVTNPKE.-**  ([Ions score 19](http://www.matrixscience.com/cgi/peptide_view.pl?file=../data/20100727/FtemCacah.dat&query=673&hit=1&index=gi|126030594&px=1&section=5&ave_thresh=42&_ignoreionsscorebelow=0&report=5&_sigthreshold=0.05&_msresflags=1089&_msresflags2=2&percolate=-1&percolate_rt=0))

Bottom of Form

**Protein View**

**Spot 1050**

Top of Form

Match to: **gi|219978** Score: **188**

**prealbumin [Homo sapiens]**

Found in search of C:\DOCUME~1\DRAF40~1.JIT\LOCALS~1\Temp\mas1D.tmp

Nominal mass (M_r_): **16023**; Calculated pI value: **5.52**

NCBI BLAST search of [gi|219978](http://www.ncbi.nlm.nih.gov/blast/Blast.cgi?ALIGNMENTS=50&ALIGNMENT_VIEW=Pairwise&AUTO_FORMAT=Semiauto&CDD_SEARCH=on&CLIENT=web&COMPOSITION_BASED_STATISTICS=on&DATABASE=nr&DESCRIPTIONS=100&ENTREZ_QUERY=(none)&EXPECT=10&FILTER=L&FORMAT_BLOCK_ON_RESPAGE=None&FORMAT_OBJECT=Alignment&FORMAT_TYPE=HTML&GAPCOSTS=11+1&I_THRESH=0.001&LAYOUT=TwoWindows&MATRIX_NAME=BLOSUM62&NCBI_GI=on&PAGE=Proteins&PROGRAM=blastp&QUERY=MASHRLLLLCLAGLVFVSEAGPTGTGESKCPLMVKVLDAVRGSPAINVAMHVFRKAADDTWEPFASGKTSESGELHGLTTEEEFVEGIYKVEIDTKSYWKALGISPFHEHAEVVFTANDSGPRRYTIAALLSPYSYSTTAVVTNPKE&SERVICE=plain&SET_DEFAULTS.x=9&SET_DEFAULTS.y=5&SHOW_OVERVIEW=on&WORD_SIZE=3&END_OF_HTTPGET=Yes) against nr

Unformatted [sequence string](http://www.matrixscience.com/cgi/getseq.pl?NCBInr+gi|219978+seq) for pasting into other applications

Taxonomy: [Homo sapiens](http://www.ncbi.nlm.nih.gov/Taxonomy/Browser/wwwtax.cgi?lvl=0&id=9606)

Links to retrieve other entries containing this sequence from NCBI Entrez:

[gi|387000](http://www.ncbi.nlm.nih.gov/entrez/query.fcgi?cmd=search&db=protein&doptcmdl=genpept&tool=mascot&term=387000) from [Homo sapiens](http://www.ncbi.nlm.nih.gov/Taxonomy/Browser/wwwtax.cgi?lvl=0&id=9606)

Fixed modifications: Carbamidomethyl (C)

Variable modifications: Oxidation (M)

Cleavage by Trypsin: cuts C-term side of KR unless next residue is P

Sequence Coverage: **46%**

Matched peptides shown in **Bold Red**

**1** MASHRLLLLC LAGLVFVSEA GPTGTGESKC PLMVKVLDAV RGSPAINVAM

**51** HVFR**KAADDT WEPFASGKTS ESGELHGLTT EEEFVEGIYK VEIDTKSYWK**

**101 ALGISPFHEH AEVVFTANDS GPR**RYTIAAL LSPYSYSTTA VVTNPKE

  Residue Number  Increasing Mass  Decreasing Mass

**Start - End Observed Mr(expt) Mr(calc) Delta Miss Sequence**

**55 - 68 761.8814 1521.7483 1521.7100 0.0383 1 R.KAADDTWEPFASGK.T**  ([Ions score 30](http://www.matrixscience.com/cgi/peptide_view.pl?file=../data/20100727/FtemCaamt.dat&query=588&hit=1&index=gi|219978&px=1&section=5&ave_thresh=43&_ignoreionsscorebelow=0&report=5&_sigthreshold=0.05&_msresflags=1089&_msresflags2=2&percolate=-1&percolate_rt=0))

**56 - 68 697.3776 1392.7407 1393.6150 -0.8743 0 K.AADDTWEPFASGK.T**  ([Ions score 96](http://www.matrixscience.com/cgi/peptide_view.pl?file=../data/20100727/FtemCaamt.dat&query=550&hit=1&index=gi|219978&px=1&section=5&ave_thresh=43&_ignoreionsscorebelow=0&report=5&_sigthreshold=0.05&_msresflags=1089&_msresflags2=2&percolate=-1&percolate_rt=0))

**69 - 90 819.4158 2455.2255 2454.1438 1.0817 0 K.TSESGELHGLTTEEEFVEGIYK.V**  ([Ions score 79](http://www.matrixscience.com/cgi/peptide_view.pl?file=../data/20100727/FtemCaamt.dat&query=660&hit=1&index=gi|219978&px=1&section=5&ave_thresh=43&_ignoreionsscorebelow=0&report=5&_sigthreshold=0.05&_msresflags=1089&_msresflags2=2&percolate=-1&percolate_rt=0))

**91 - 100 635.3575 1268.7004 1267.6448 1.0555 1 K.VEIDTKSYWK.A**  ([Ions score 5](http://www.matrixscience.com/cgi/peptide_view.pl?file=../data/20100727/FtemCaamt.dat&query=467&hit=2&index=gi|219978&px=1&section=5&ave_thresh=43&_ignoreionsscorebelow=0&report=5&_sigthreshold=0.05&_msresflags=1089&_msresflags2=2&percolate=-1&percolate_rt=0))

**101 - 123 818.1086 2451.3040 2450.1979 1.1061 0 K.ALGISPFHEHAEVVFTANDSGPR.R**  ([Ions score 59](http://www.matrixscience.com/cgi/peptide_view.pl?file=../data/20100727/FtemCaamt.dat&query=659&hit=1&index=gi|219978&px=1&section=5&ave_thresh=43&_ignoreionsscorebelow=0&report=5&_sigthreshold=0.05&_msresflags=1089&_msresflags2=2&percolate=-1&percolate_rt=0))

**Protein View**

**Spot 1049**

Top of Form

Match to: **gi|443295** Score: **262**

**Chain A, The X-Ray Crystal Structure Refinements Of Normal Human Transthyretin And The Amyloidogenic Val30met Variant To 1.7 Angstroms Resolution**

Found in search of C:\DOCUME~1\DRAF40~1.JIT\LOCALS~1\Temp\mas20.tmp

Nominal mass (M_r_): **13810**; Calculated pI value: **5.35**

NCBI BLAST search of [gi|443295](http://www.ncbi.nlm.nih.gov/blast/Blast.cgi?ALIGNMENTS=50&ALIGNMENT_VIEW=Pairwise&AUTO_FORMAT=Semiauto&CDD_SEARCH=on&CLIENT=web&COMPOSITION_BASED_STATISTICS=on&DATABASE=nr&DESCRIPTIONS=100&ENTREZ_QUERY=(none)&EXPECT=10&FILTER=L&FORMAT_BLOCK_ON_RESPAGE=None&FORMAT_OBJECT=Alignment&FORMAT_TYPE=HTML&GAPCOSTS=11+1&I_THRESH=0.001&LAYOUT=TwoWindows&MATRIX_NAME=BLOSUM62&NCBI_GI=on&PAGE=Proteins&PROGRAM=blastp&QUERY=GPTGTGESKCPLMVKVLDAVRGSPAINVAVHVFRKAADDTWEPFASGKTSESGELHGLTTEEEFVEGIYKVEIDTKSYWKALGISPFHEHAEVVFTANDSGPRRYTIAALLSPYSYSTTAVVTNPKE&SERVICE=plain&SET_DEFAULTS.x=9&SET_DEFAULTS.y=5&SHOW_OVERVIEW=on&WORD_SIZE=3&END_OF_HTTPGET=Yes) against nr

Unformatted [sequence string](http://www.matrixscience.com/cgi/getseq.pl?NCBInr+gi|443295+seq) for pasting into other applications

Taxonomy: [Homo sapiens](http://www.ncbi.nlm.nih.gov/Taxonomy/Browser/wwwtax.cgi?lvl=0&id=9606)

Links to retrieve other entries containing this sequence from NCBI Entrez:

[gi|443296](http://www.ncbi.nlm.nih.gov/entrez/query.fcgi?cmd=search&db=protein&doptcmdl=genpept&tool=mascot&term=443296) from [Homo sapiens](http://www.ncbi.nlm.nih.gov/Taxonomy/Browser/wwwtax.cgi?lvl=0&id=9606)

[gi|1127058](http://www.ncbi.nlm.nih.gov/entrez/query.fcgi?cmd=search&db=protein&doptcmdl=genpept&tool=mascot&term=1127058) from [Homo sapiens](http://www.ncbi.nlm.nih.gov/Taxonomy/Browser/wwwtax.cgi?lvl=0&id=9606)

[gi|1127059](http://www.ncbi.nlm.nih.gov/entrez/query.fcgi?cmd=search&db=protein&doptcmdl=genpept&tool=mascot&term=1127059) from [Homo sapiens](http://www.ncbi.nlm.nih.gov/Taxonomy/Browser/wwwtax.cgi?lvl=0&id=9606)

[gi|2098264](http://www.ncbi.nlm.nih.gov/entrez/query.fcgi?cmd=search&db=protein&doptcmdl=genpept&tool=mascot&term=2098264) from [Homo sapiens](http://www.ncbi.nlm.nih.gov/Taxonomy/Browser/wwwtax.cgi?lvl=0&id=9606)

[gi|2098265](http://www.ncbi.nlm.nih.gov/entrez/query.fcgi?cmd=search&db=protein&doptcmdl=genpept&tool=mascot&term=2098265) from [Homo sapiens](http://www.ncbi.nlm.nih.gov/Taxonomy/Browser/wwwtax.cgi?lvl=0&id=9606)

[gi|2098266](http://www.ncbi.nlm.nih.gov/entrez/query.fcgi?cmd=search&db=protein&doptcmdl=genpept&tool=mascot&term=2098266) from [Homo sapiens](http://www.ncbi.nlm.nih.gov/Taxonomy/Browser/wwwtax.cgi?lvl=0&id=9606)

[gi|2098267](http://www.ncbi.nlm.nih.gov/entrez/query.fcgi?cmd=search&db=protein&doptcmdl=genpept&tool=mascot&term=2098267) from [Homo sapiens](http://www.ncbi.nlm.nih.gov/Taxonomy/Browser/wwwtax.cgi?lvl=0&id=9606)

[gi|3401967](http://www.ncbi.nlm.nih.gov/entrez/query.fcgi?cmd=search&db=protein&doptcmdl=genpept&tool=mascot&term=3401967) from [Homo sapiens](http://www.ncbi.nlm.nih.gov/Taxonomy/Browser/wwwtax.cgi?lvl=0&id=9606)

[gi|3401968](http://www.ncbi.nlm.nih.gov/entrez/query.fcgi?cmd=search&db=protein&doptcmdl=genpept&tool=mascot&term=3401968) from [Homo sapiens](http://www.ncbi.nlm.nih.gov/Taxonomy/Browser/wwwtax.cgi?lvl=0&id=9606)

[gi|3401969](http://www.ncbi.nlm.nih.gov/entrez/query.fcgi?cmd=search&db=protein&doptcmdl=genpept&tool=mascot&term=3401969) from [Homo sapiens](http://www.ncbi.nlm.nih.gov/Taxonomy/Browser/wwwtax.cgi?lvl=0&id=9606)

[gi|3401970](http://www.ncbi.nlm.nih.gov/entrez/query.fcgi?cmd=search&db=protein&doptcmdl=genpept&tool=mascot&term=3401970) from [Homo sapiens](http://www.ncbi.nlm.nih.gov/Taxonomy/Browser/wwwtax.cgi?lvl=0&id=9606)

[gi|10120703](http://www.ncbi.nlm.nih.gov/entrez/query.fcgi?cmd=search&db=protein&doptcmdl=genpept&tool=mascot&term=10120703) from [Homo sapiens](http://www.ncbi.nlm.nih.gov/Taxonomy/Browser/wwwtax.cgi?lvl=0&id=9606)

[gi|10120704](http://www.ncbi.nlm.nih.gov/entrez/query.fcgi?cmd=search&db=protein&doptcmdl=genpept&tool=mascot&term=10120704) from [Homo sapiens](http://www.ncbi.nlm.nih.gov/Taxonomy/Browser/wwwtax.cgi?lvl=0&id=9606)

[gi|10120705](http://www.ncbi.nlm.nih.gov/entrez/query.fcgi?cmd=search&db=protein&doptcmdl=genpept&tool=mascot&term=10120705) from [Homo sapiens](http://www.ncbi.nlm.nih.gov/Taxonomy/Browser/wwwtax.cgi?lvl=0&id=9606)

[gi|10120706](http://www.ncbi.nlm.nih.gov/entrez/query.fcgi?cmd=search&db=protein&doptcmdl=genpept&tool=mascot&term=10120706) from [Homo sapiens](http://www.ncbi.nlm.nih.gov/Taxonomy/Browser/wwwtax.cgi?lvl=0&id=9606)

[gi|10120707](http://www.ncbi.nlm.nih.gov/entrez/query.fcgi?cmd=search&db=protein&doptcmdl=genpept&tool=mascot&term=10120707) from [Homo sapiens](http://www.ncbi.nlm.nih.gov/Taxonomy/Browser/wwwtax.cgi?lvl=0&id=9606)

[gi|10120708](http://www.ncbi.nlm.nih.gov/entrez/query.fcgi?cmd=search&db=protein&doptcmdl=genpept&tool=mascot&term=10120708) from [Homo sapiens](http://www.ncbi.nlm.nih.gov/Taxonomy/Browser/wwwtax.cgi?lvl=0&id=9606)

[gi|10835700](http://www.ncbi.nlm.nih.gov/entrez/query.fcgi?cmd=search&db=protein&doptcmdl=genpept&tool=mascot&term=10835700) from [Homo sapiens](http://www.ncbi.nlm.nih.gov/Taxonomy/Browser/wwwtax.cgi?lvl=0&id=9606)

[gi|10835701](http://www.ncbi.nlm.nih.gov/entrez/query.fcgi?cmd=search&db=protein&doptcmdl=genpept&tool=mascot&term=10835701) from [Homo sapiens](http://www.ncbi.nlm.nih.gov/Taxonomy/Browser/wwwtax.cgi?lvl=0&id=9606)

[gi|20150160](http://www.ncbi.nlm.nih.gov/entrez/query.fcgi?cmd=search&db=protein&doptcmdl=genpept&tool=mascot&term=20150160) from [Homo sapiens](http://www.ncbi.nlm.nih.gov/Taxonomy/Browser/wwwtax.cgi?lvl=0&id=9606)

[gi|20150161](http://www.ncbi.nlm.nih.gov/entrez/query.fcgi?cmd=search&db=protein&doptcmdl=genpept&tool=mascot&term=20150161) from [Homo sapiens](http://www.ncbi.nlm.nih.gov/Taxonomy/Browser/wwwtax.cgi?lvl=0&id=9606)

[gi|20150162](http://www.ncbi.nlm.nih.gov/entrez/query.fcgi?cmd=search&db=protein&doptcmdl=genpept&tool=mascot&term=20150162) from [Homo sapiens](http://www.ncbi.nlm.nih.gov/Taxonomy/Browser/wwwtax.cgi?lvl=0&id=9606)

[gi|20150163](http://www.ncbi.nlm.nih.gov/entrez/query.fcgi?cmd=search&db=protein&doptcmdl=genpept&tool=mascot&term=20150163) from [Homo sapiens](http://www.ncbi.nlm.nih.gov/Taxonomy/Browser/wwwtax.cgi?lvl=0&id=9606)

[gi|20150164](http://www.ncbi.nlm.nih.gov/entrez/query.fcgi?cmd=search&db=protein&doptcmdl=genpept&tool=mascot&term=20150164) from [Homo sapiens](http://www.ncbi.nlm.nih.gov/Taxonomy/Browser/wwwtax.cgi?lvl=0&id=9606)

[gi|20150165](http://www.ncbi.nlm.nih.gov/entrez/query.fcgi?cmd=search&db=protein&doptcmdl=genpept&tool=mascot&term=20150165) from [Homo sapiens](http://www.ncbi.nlm.nih.gov/Taxonomy/Browser/wwwtax.cgi?lvl=0&id=9606)

[gi|20150166](http://www.ncbi.nlm.nih.gov/entrez/query.fcgi?cmd=search&db=protein&doptcmdl=genpept&tool=mascot&term=20150166) from [Homo sapiens](http://www.ncbi.nlm.nih.gov/Taxonomy/Browser/wwwtax.cgi?lvl=0&id=9606)

[gi|20150167](http://www.ncbi.nlm.nih.gov/entrez/query.fcgi?cmd=search&db=protein&doptcmdl=genpept&tool=mascot&term=20150167) from [Homo sapiens](http://www.ncbi.nlm.nih.gov/Taxonomy/Browser/wwwtax.cgi?lvl=0&id=9606)

[gi|56554010](http://www.ncbi.nlm.nih.gov/entrez/query.fcgi?cmd=search&db=protein&doptcmdl=genpept&tool=mascot&term=56554010) from [Homo sapiens](http://www.ncbi.nlm.nih.gov/Taxonomy/Browser/wwwtax.cgi?lvl=0&id=9606)

[gi|56554011](http://www.ncbi.nlm.nih.gov/entrez/query.fcgi?cmd=search&db=protein&doptcmdl=genpept&tool=mascot&term=56554011) from [Homo sapiens](http://www.ncbi.nlm.nih.gov/Taxonomy/Browser/wwwtax.cgi?lvl=0&id=9606)

[gi|56554064](http://www.ncbi.nlm.nih.gov/entrez/query.fcgi?cmd=search&db=protein&doptcmdl=genpept&tool=mascot&term=56554064) from [Homo sapiens](http://www.ncbi.nlm.nih.gov/Taxonomy/Browser/wwwtax.cgi?lvl=0&id=9606)

[gi|56554065](http://www.ncbi.nlm.nih.gov/entrez/query.fcgi?cmd=search&db=protein&doptcmdl=genpept&tool=mascot&term=56554065) from [Homo sapiens](http://www.ncbi.nlm.nih.gov/Taxonomy/Browser/wwwtax.cgi?lvl=0&id=9606)

[gi|56554066](http://www.ncbi.nlm.nih.gov/entrez/query.fcgi?cmd=search&db=protein&doptcmdl=genpept&tool=mascot&term=56554066) from [Homo sapiens](http://www.ncbi.nlm.nih.gov/Taxonomy/Browser/wwwtax.cgi?lvl=0&id=9606)

[gi|56554067](http://www.ncbi.nlm.nih.gov/entrez/query.fcgi?cmd=search&db=protein&doptcmdl=genpept&tool=mascot&term=56554067) from [Homo sapiens](http://www.ncbi.nlm.nih.gov/Taxonomy/Browser/wwwtax.cgi?lvl=0&id=9606)

[gi|71041677](http://www.ncbi.nlm.nih.gov/entrez/query.fcgi?cmd=search&db=protein&doptcmdl=genpept&tool=mascot&term=71041677) from [Homo sapiens](http://www.ncbi.nlm.nih.gov/Taxonomy/Browser/wwwtax.cgi?lvl=0&id=9606)

[gi|71041678](http://www.ncbi.nlm.nih.gov/entrez/query.fcgi?cmd=search&db=protein&doptcmdl=genpept&tool=mascot&term=71041678) from [Homo sapiens](http://www.ncbi.nlm.nih.gov/Taxonomy/Browser/wwwtax.cgi?lvl=0&id=9606)

[gi|71042206](http://www.ncbi.nlm.nih.gov/entrez/query.fcgi?cmd=search&db=protein&doptcmdl=genpept&tool=mascot&term=71042206) from [Homo sapiens](http://www.ncbi.nlm.nih.gov/Taxonomy/Browser/wwwtax.cgi?lvl=0&id=9606)

[gi|71042207](http://www.ncbi.nlm.nih.gov/entrez/query.fcgi?cmd=search&db=protein&doptcmdl=genpept&tool=mascot&term=71042207) from [Homo sapiens](http://www.ncbi.nlm.nih.gov/Taxonomy/Browser/wwwtax.cgi?lvl=0&id=9606)

[gi|73535560](http://www.ncbi.nlm.nih.gov/entrez/query.fcgi?cmd=search&db=protein&doptcmdl=genpept&tool=mascot&term=73535560) from [Homo sapiens](http://www.ncbi.nlm.nih.gov/Taxonomy/Browser/wwwtax.cgi?lvl=0&id=9606)

[gi|73535561](http://www.ncbi.nlm.nih.gov/entrez/query.fcgi?cmd=search&db=protein&doptcmdl=genpept&tool=mascot&term=73535561) from [Homo sapiens](http://www.ncbi.nlm.nih.gov/Taxonomy/Browser/wwwtax.cgi?lvl=0&id=9606)

[gi|82408024](http://www.ncbi.nlm.nih.gov/entrez/query.fcgi?cmd=search&db=protein&doptcmdl=genpept&tool=mascot&term=82408024) from [Homo sapiens](http://www.ncbi.nlm.nih.gov/Taxonomy/Browser/wwwtax.cgi?lvl=0&id=9606)

[gi|82408025](http://www.ncbi.nlm.nih.gov/entrez/query.fcgi?cmd=search&db=protein&doptcmdl=genpept&tool=mascot&term=82408025) from [Homo sapiens](http://www.ncbi.nlm.nih.gov/Taxonomy/Browser/wwwtax.cgi?lvl=0&id=9606)

[gi|82408042](http://www.ncbi.nlm.nih.gov/entrez/query.fcgi?cmd=search&db=protein&doptcmdl=genpept&tool=mascot&term=82408042) from [Homo sapiens](http://www.ncbi.nlm.nih.gov/Taxonomy/Browser/wwwtax.cgi?lvl=0&id=9606)

[gi|82408043](http://www.ncbi.nlm.nih.gov/entrez/query.fcgi?cmd=search&db=protein&doptcmdl=genpept&tool=mascot&term=82408043) from [Homo sapiens](http://www.ncbi.nlm.nih.gov/Taxonomy/Browser/wwwtax.cgi?lvl=0&id=9606)

[gi|85544634](http://www.ncbi.nlm.nih.gov/entrez/query.fcgi?cmd=search&db=protein&doptcmdl=genpept&tool=mascot&term=85544634) from [Homo sapiens](http://www.ncbi.nlm.nih.gov/Taxonomy/Browser/wwwtax.cgi?lvl=0&id=9606)

[gi|85544635](http://www.ncbi.nlm.nih.gov/entrez/query.fcgi?cmd=search&db=protein&doptcmdl=genpept&tool=mascot&term=85544635) from [Homo sapiens](http://www.ncbi.nlm.nih.gov/Taxonomy/Browser/wwwtax.cgi?lvl=0&id=9606)

[gi|85544641](http://www.ncbi.nlm.nih.gov/entrez/query.fcgi?cmd=search&db=protein&doptcmdl=genpept&tool=mascot&term=85544641) from [Homo sapiens](http://www.ncbi.nlm.nih.gov/Taxonomy/Browser/wwwtax.cgi?lvl=0&id=9606)

[gi|85544642](http://www.ncbi.nlm.nih.gov/entrez/query.fcgi?cmd=search&db=protein&doptcmdl=genpept&tool=mascot&term=85544642) from [Homo sapiens](http://www.ncbi.nlm.nih.gov/Taxonomy/Browser/wwwtax.cgi?lvl=0&id=9606)

[gi|85544663](http://www.ncbi.nlm.nih.gov/entrez/query.fcgi?cmd=search&db=protein&doptcmdl=genpept&tool=mascot&term=85544663) from [Homo sapiens](http://www.ncbi.nlm.nih.gov/Taxonomy/Browser/wwwtax.cgi?lvl=0&id=9606)

[gi|85544664](http://www.ncbi.nlm.nih.gov/entrez/query.fcgi?cmd=search&db=protein&doptcmdl=genpept&tool=mascot&term=85544664) from [Homo sapiens](http://www.ncbi.nlm.nih.gov/Taxonomy/Browser/wwwtax.cgi?lvl=0&id=9606)

[gi|90109527](http://www.ncbi.nlm.nih.gov/entrez/query.fcgi?cmd=search&db=protein&doptcmdl=genpept&tool=mascot&term=90109527) from [Homo sapiens](http://www.ncbi.nlm.nih.gov/Taxonomy/Browser/wwwtax.cgi?lvl=0&id=9606)

[gi|90109528](http://www.ncbi.nlm.nih.gov/entrez/query.fcgi?cmd=search&db=protein&doptcmdl=genpept&tool=mascot&term=90109528) from [Homo sapiens](http://www.ncbi.nlm.nih.gov/Taxonomy/Browser/wwwtax.cgi?lvl=0&id=9606)

[gi|93279880](http://www.ncbi.nlm.nih.gov/entrez/query.fcgi?cmd=search&db=protein&doptcmdl=genpept&tool=mascot&term=93279880) from [Homo sapiens](http://www.ncbi.nlm.nih.gov/Taxonomy/Browser/wwwtax.cgi?lvl=0&id=9606)

[gi|93279881](http://www.ncbi.nlm.nih.gov/entrez/query.fcgi?cmd=search&db=protein&doptcmdl=genpept&tool=mascot&term=93279881) from [Homo sapiens](http://www.ncbi.nlm.nih.gov/Taxonomy/Browser/wwwtax.cgi?lvl=0&id=9606)

[gi|93279938](http://www.ncbi.nlm.nih.gov/entrez/query.fcgi?cmd=search&db=protein&doptcmdl=genpept&tool=mascot&term=93279938) from [Homo sapiens](http://www.ncbi.nlm.nih.gov/Taxonomy/Browser/wwwtax.cgi?lvl=0&id=9606)

[gi|93279939](http://www.ncbi.nlm.nih.gov/entrez/query.fcgi?cmd=search&db=protein&doptcmdl=genpept&tool=mascot&term=93279939) from [Homo sapiens](http://www.ncbi.nlm.nih.gov/Taxonomy/Browser/wwwtax.cgi?lvl=0&id=9606)

[gi|93279947](http://www.ncbi.nlm.nih.gov/entrez/query.fcgi?cmd=search&db=protein&doptcmdl=genpept&tool=mascot&term=93279947) from [Homo sapiens](http://www.ncbi.nlm.nih.gov/Taxonomy/Browser/wwwtax.cgi?lvl=0&id=9606)

[gi|93279948](http://www.ncbi.nlm.nih.gov/entrez/query.fcgi?cmd=search&db=protein&doptcmdl=genpept&tool=mascot&term=93279948) from [Homo sapiens](http://www.ncbi.nlm.nih.gov/Taxonomy/Browser/wwwtax.cgi?lvl=0&id=9606)

[gi|112490037](http://www.ncbi.nlm.nih.gov/entrez/query.fcgi?cmd=search&db=protein&doptcmdl=genpept&tool=mascot&term=112490037) from [Homo sapiens](http://www.ncbi.nlm.nih.gov/Taxonomy/Browser/wwwtax.cgi?lvl=0&id=9606)

[gi|112490038](http://www.ncbi.nlm.nih.gov/entrez/query.fcgi?cmd=search&db=protein&doptcmdl=genpept&tool=mascot&term=112490038) from [Homo sapiens](http://www.ncbi.nlm.nih.gov/Taxonomy/Browser/wwwtax.cgi?lvl=0&id=9606)

[gi|126030518](http://www.ncbi.nlm.nih.gov/entrez/query.fcgi?cmd=search&db=protein&doptcmdl=genpept&tool=mascot&term=126030518) from [Homo sapiens](http://www.ncbi.nlm.nih.gov/Taxonomy/Browser/wwwtax.cgi?lvl=0&id=9606)

[gi|126030519](http://www.ncbi.nlm.nih.gov/entrez/query.fcgi?cmd=search&db=protein&doptcmdl=genpept&tool=mascot&term=126030519) from [Homo sapiens](http://www.ncbi.nlm.nih.gov/Taxonomy/Browser/wwwtax.cgi?lvl=0&id=9606)

[gi|165761299](http://www.ncbi.nlm.nih.gov/entrez/query.fcgi?cmd=search&db=protein&doptcmdl=genpept&tool=mascot&term=165761299) from [Homo sapiens](http://www.ncbi.nlm.nih.gov/Taxonomy/Browser/wwwtax.cgi?lvl=0&id=9606)

[gi|165761300](http://www.ncbi.nlm.nih.gov/entrez/query.fcgi?cmd=search&db=protein&doptcmdl=genpept&tool=mascot&term=165761300) from [Homo sapiens](http://www.ncbi.nlm.nih.gov/Taxonomy/Browser/wwwtax.cgi?lvl=0&id=9606)

[gi|167013274](http://www.ncbi.nlm.nih.gov/entrez/query.fcgi?cmd=search&db=protein&doptcmdl=genpept&tool=mascot&term=167013274) from [Homo sapiens](http://www.ncbi.nlm.nih.gov/Taxonomy/Browser/wwwtax.cgi?lvl=0&id=9606)

[gi|167013275](http://www.ncbi.nlm.nih.gov/entrez/query.fcgi?cmd=search&db=protein&doptcmdl=genpept&tool=mascot&term=167013275) from [Homo sapiens](http://www.ncbi.nlm.nih.gov/Taxonomy/Browser/wwwtax.cgi?lvl=0&id=9606)

[gi|167013276](http://www.ncbi.nlm.nih.gov/entrez/query.fcgi?cmd=search&db=protein&doptcmdl=genpept&tool=mascot&term=167013276) from [Homo sapiens](http://www.ncbi.nlm.nih.gov/Taxonomy/Browser/wwwtax.cgi?lvl=0&id=9606)

[gi|167013277](http://www.ncbi.nlm.nih.gov/entrez/query.fcgi?cmd=search&db=protein&doptcmdl=genpept&tool=mascot&term=167013277) from [Homo sapiens](http://www.ncbi.nlm.nih.gov/Taxonomy/Browser/wwwtax.cgi?lvl=0&id=9606)

[gi|167013278](http://www.ncbi.nlm.nih.gov/entrez/query.fcgi?cmd=search&db=protein&doptcmdl=genpept&tool=mascot&term=167013278) from [Homo sapiens](http://www.ncbi.nlm.nih.gov/Taxonomy/Browser/wwwtax.cgi?lvl=0&id=9606)

[gi|167013279](http://www.ncbi.nlm.nih.gov/entrez/query.fcgi?cmd=search&db=protein&doptcmdl=genpept&tool=mascot&term=167013279) from [Homo sapiens](http://www.ncbi.nlm.nih.gov/Taxonomy/Browser/wwwtax.cgi?lvl=0&id=9606)

[gi|167013280](http://www.ncbi.nlm.nih.gov/entrez/query.fcgi?cmd=search&db=protein&doptcmdl=genpept&tool=mascot&term=167013280) from [Homo sapiens](http://www.ncbi.nlm.nih.gov/Taxonomy/Browser/wwwtax.cgi?lvl=0&id=9606)

[gi|167013281](http://www.ncbi.nlm.nih.gov/entrez/query.fcgi?cmd=search&db=protein&doptcmdl=genpept&tool=mascot&term=167013281) from [Homo sapiens](http://www.ncbi.nlm.nih.gov/Taxonomy/Browser/wwwtax.cgi?lvl=0&id=9606)

[gi|188596508](http://www.ncbi.nlm.nih.gov/entrez/query.fcgi?cmd=search&db=protein&doptcmdl=genpept&tool=mascot&term=188596508) from [Homo sapiens](http://www.ncbi.nlm.nih.gov/Taxonomy/Browser/wwwtax.cgi?lvl=0&id=9606)

[gi|188596509](http://www.ncbi.nlm.nih.gov/entrez/query.fcgi?cmd=search&db=protein&doptcmdl=genpept&tool=mascot&term=188596509) from [Homo sapiens](http://www.ncbi.nlm.nih.gov/Taxonomy/Browser/wwwtax.cgi?lvl=0&id=9606)

[gi|196049767](http://www.ncbi.nlm.nih.gov/entrez/query.fcgi?cmd=search&db=protein&doptcmdl=genpept&tool=mascot&term=196049767) from [Homo sapiens](http://www.ncbi.nlm.nih.gov/Taxonomy/Browser/wwwtax.cgi?lvl=0&id=9606)

[gi|196049768](http://www.ncbi.nlm.nih.gov/entrez/query.fcgi?cmd=search&db=protein&doptcmdl=genpept&tool=mascot&term=196049768) from [Homo sapiens](http://www.ncbi.nlm.nih.gov/Taxonomy/Browser/wwwtax.cgi?lvl=0&id=9606)

[gi|196049804](http://www.ncbi.nlm.nih.gov/entrez/query.fcgi?cmd=search&db=protein&doptcmdl=genpept&tool=mascot&term=196049804) from [Homo sapiens](http://www.ncbi.nlm.nih.gov/Taxonomy/Browser/wwwtax.cgi?lvl=0&id=9606)

[gi|196049805](http://www.ncbi.nlm.nih.gov/entrez/query.fcgi?cmd=search&db=protein&doptcmdl=genpept&tool=mascot&term=196049805) from [Homo sapiens](http://www.ncbi.nlm.nih.gov/Taxonomy/Browser/wwwtax.cgi?lvl=0&id=9606)

[gi|210060937](http://www.ncbi.nlm.nih.gov/entrez/query.fcgi?cmd=search&db=protein&doptcmdl=genpept&tool=mascot&term=210060937) from [Homo sapiens](http://www.ncbi.nlm.nih.gov/Taxonomy/Browser/wwwtax.cgi?lvl=0&id=9606)

[gi|210060938](http://www.ncbi.nlm.nih.gov/entrez/query.fcgi?cmd=search&db=protein&doptcmdl=genpept&tool=mascot&term=210060938) from [Homo sapiens](http://www.ncbi.nlm.nih.gov/Taxonomy/Browser/wwwtax.cgi?lvl=0&id=9606)

[gi|210060939](http://www.ncbi.nlm.nih.gov/entrez/query.fcgi?cmd=search&db=protein&doptcmdl=genpept&tool=mascot&term=210060939) from [Homo sapiens](http://www.ncbi.nlm.nih.gov/Taxonomy/Browser/wwwtax.cgi?lvl=0&id=9606)

[gi|210060940](http://www.ncbi.nlm.nih.gov/entrez/query.fcgi?cmd=search&db=protein&doptcmdl=genpept&tool=mascot&term=210060940) from [Homo sapiens](http://www.ncbi.nlm.nih.gov/Taxonomy/Browser/wwwtax.cgi?lvl=0&id=9606)

[gi|210060941](http://www.ncbi.nlm.nih.gov/entrez/query.fcgi?cmd=search&db=protein&doptcmdl=genpept&tool=mascot&term=210060941) from [Homo sapiens](http://www.ncbi.nlm.nih.gov/Taxonomy/Browser/wwwtax.cgi?lvl=0&id=9606)

[gi|210060942](http://www.ncbi.nlm.nih.gov/entrez/query.fcgi?cmd=search&db=protein&doptcmdl=genpept&tool=mascot&term=210060942) from [Homo sapiens](http://www.ncbi.nlm.nih.gov/Taxonomy/Browser/wwwtax.cgi?lvl=0&id=9606)

[gi|210060943](http://www.ncbi.nlm.nih.gov/entrez/query.fcgi?cmd=search&db=protein&doptcmdl=genpept&tool=mascot&term=210060943) from [Homo sapiens](http://www.ncbi.nlm.nih.gov/Taxonomy/Browser/wwwtax.cgi?lvl=0&id=9606)

[gi|210060944](http://www.ncbi.nlm.nih.gov/entrez/query.fcgi?cmd=search&db=protein&doptcmdl=genpept&tool=mascot&term=210060944) from [Homo sapiens](http://www.ncbi.nlm.nih.gov/Taxonomy/Browser/wwwtax.cgi?lvl=0&id=9606)

[gi|210060945](http://www.ncbi.nlm.nih.gov/entrez/query.fcgi?cmd=search&db=protein&doptcmdl=genpept&tool=mascot&term=210060945) from [Homo sapiens](http://www.ncbi.nlm.nih.gov/Taxonomy/Browser/wwwtax.cgi?lvl=0&id=9606)

[gi|210060946](http://www.ncbi.nlm.nih.gov/entrez/query.fcgi?cmd=search&db=protein&doptcmdl=genpept&tool=mascot&term=210060946) from [Homo sapiens](http://www.ncbi.nlm.nih.gov/Taxonomy/Browser/wwwtax.cgi?lvl=0&id=9606)

[gi|212374942](http://www.ncbi.nlm.nih.gov/entrez/query.fcgi?cmd=search&db=protein&doptcmdl=genpept&tool=mascot&term=212374942) from [Homo sapiens](http://www.ncbi.nlm.nih.gov/Taxonomy/Browser/wwwtax.cgi?lvl=0&id=9606)

[gi|212374943](http://www.ncbi.nlm.nih.gov/entrez/query.fcgi?cmd=search&db=protein&doptcmdl=genpept&tool=mascot&term=212374943) from [Homo sapiens](http://www.ncbi.nlm.nih.gov/Taxonomy/Browser/wwwtax.cgi?lvl=0&id=9606)

[gi|212374944](http://www.ncbi.nlm.nih.gov/entrez/query.fcgi?cmd=search&db=protein&doptcmdl=genpept&tool=mascot&term=212374944) from [Homo sapiens](http://www.ncbi.nlm.nih.gov/Taxonomy/Browser/wwwtax.cgi?lvl=0&id=9606)

[gi|212374945](http://www.ncbi.nlm.nih.gov/entrez/query.fcgi?cmd=search&db=protein&doptcmdl=genpept&tool=mascot&term=212374945) from [Homo sapiens](http://www.ncbi.nlm.nih.gov/Taxonomy/Browser/wwwtax.cgi?lvl=0&id=9606)

[gi|254839337](http://www.ncbi.nlm.nih.gov/entrez/query.fcgi?cmd=search&db=protein&doptcmdl=genpept&tool=mascot&term=254839337) from [Homo sapiens](http://www.ncbi.nlm.nih.gov/Taxonomy/Browser/wwwtax.cgi?lvl=0&id=9606)

[gi|254839338](http://www.ncbi.nlm.nih.gov/entrez/query.fcgi?cmd=search&db=protein&doptcmdl=genpept&tool=mascot&term=254839338) from [Homo sapiens](http://www.ncbi.nlm.nih.gov/Taxonomy/Browser/wwwtax.cgi?lvl=0&id=9606)

[gi|254839350](http://www.ncbi.nlm.nih.gov/entrez/query.fcgi?cmd=search&db=protein&doptcmdl=genpept&tool=mascot&term=254839350) from [Homo sapiens](http://www.ncbi.nlm.nih.gov/Taxonomy/Browser/wwwtax.cgi?lvl=0&id=9606)

[gi|254839351](http://www.ncbi.nlm.nih.gov/entrez/query.fcgi?cmd=search&db=protein&doptcmdl=genpept&tool=mascot&term=254839351) from [Homo sapiens](http://www.ncbi.nlm.nih.gov/Taxonomy/Browser/wwwtax.cgi?lvl=0&id=9606)

[gi|254839352](http://www.ncbi.nlm.nih.gov/entrez/query.fcgi?cmd=search&db=protein&doptcmdl=genpept&tool=mascot&term=254839352) from [Homo sapiens](http://www.ncbi.nlm.nih.gov/Taxonomy/Browser/wwwtax.cgi?lvl=0&id=9606)

[gi|254839353](http://www.ncbi.nlm.nih.gov/entrez/query.fcgi?cmd=search&db=protein&doptcmdl=genpept&tool=mascot&term=254839353) from [Homo sapiens](http://www.ncbi.nlm.nih.gov/Taxonomy/Browser/wwwtax.cgi?lvl=0&id=9606)

[gi|254839354](http://www.ncbi.nlm.nih.gov/entrez/query.fcgi?cmd=search&db=protein&doptcmdl=genpept&tool=mascot&term=254839354) from [Homo sapiens](http://www.ncbi.nlm.nih.gov/Taxonomy/Browser/wwwtax.cgi?lvl=0&id=9606)

[gi|254839355](http://www.ncbi.nlm.nih.gov/entrez/query.fcgi?cmd=search&db=protein&doptcmdl=genpept&tool=mascot&term=254839355) from [Homo sapiens](http://www.ncbi.nlm.nih.gov/Taxonomy/Browser/wwwtax.cgi?lvl=0&id=9606)

[gi|268612512](http://www.ncbi.nlm.nih.gov/entrez/query.fcgi?cmd=search&db=protein&doptcmdl=genpept&tool=mascot&term=268612512) from [Homo sapiens](http://www.ncbi.nlm.nih.gov/Taxonomy/Browser/wwwtax.cgi?lvl=0&id=9606)

[gi|268612513](http://www.ncbi.nlm.nih.gov/entrez/query.fcgi?cmd=search&db=protein&doptcmdl=genpept&tool=mascot&term=268612513) from [Homo sapiens](http://www.ncbi.nlm.nih.gov/Taxonomy/Browser/wwwtax.cgi?lvl=0&id=9606)

[gi|268612514](http://www.ncbi.nlm.nih.gov/entrez/query.fcgi?cmd=search&db=protein&doptcmdl=genpept&tool=mascot&term=268612514) from [Homo sapiens](http://www.ncbi.nlm.nih.gov/Taxonomy/Browser/wwwtax.cgi?lvl=0&id=9606)

[gi|268612515](http://www.ncbi.nlm.nih.gov/entrez/query.fcgi?cmd=search&db=protein&doptcmdl=genpept&tool=mascot&term=268612515) from [Homo sapiens](http://www.ncbi.nlm.nih.gov/Taxonomy/Browser/wwwtax.cgi?lvl=0&id=9606)

[gi|268612516](http://www.ncbi.nlm.nih.gov/entrez/query.fcgi?cmd=search&db=protein&doptcmdl=genpept&tool=mascot&term=268612516) from [Homo sapiens](http://www.ncbi.nlm.nih.gov/Taxonomy/Browser/wwwtax.cgi?lvl=0&id=9606)

[gi|268612517](http://www.ncbi.nlm.nih.gov/entrez/query.fcgi?cmd=search&db=protein&doptcmdl=genpept&tool=mascot&term=268612517) from [Homo sapiens](http://www.ncbi.nlm.nih.gov/Taxonomy/Browser/wwwtax.cgi?lvl=0&id=9606)

[gi|281500692](http://www.ncbi.nlm.nih.gov/entrez/query.fcgi?cmd=search&db=protein&doptcmdl=genpept&tool=mascot&term=281500692) from [Homo sapiens](http://www.ncbi.nlm.nih.gov/Taxonomy/Browser/wwwtax.cgi?lvl=0&id=9606)

[gi|281500693](http://www.ncbi.nlm.nih.gov/entrez/query.fcgi?cmd=search&db=protein&doptcmdl=genpept&tool=mascot&term=281500693) from [Homo sapiens](http://www.ncbi.nlm.nih.gov/Taxonomy/Browser/wwwtax.cgi?lvl=0&id=9606)

[gi|283807026](http://www.ncbi.nlm.nih.gov/entrez/query.fcgi?cmd=search&db=protein&doptcmdl=genpept&tool=mascot&term=283807026) from [Homo sapiens](http://www.ncbi.nlm.nih.gov/Taxonomy/Browser/wwwtax.cgi?lvl=0&id=9606)

[gi|283807027](http://www.ncbi.nlm.nih.gov/entrez/query.fcgi?cmd=search&db=protein&doptcmdl=genpept&tool=mascot&term=283807027) from [Homo sapiens](http://www.ncbi.nlm.nih.gov/Taxonomy/Browser/wwwtax.cgi?lvl=0&id=9606)

[gi|283807028](http://www.ncbi.nlm.nih.gov/entrez/query.fcgi?cmd=search&db=protein&doptcmdl=genpept&tool=mascot&term=283807028) from [Homo sapiens](http://www.ncbi.nlm.nih.gov/Taxonomy/Browser/wwwtax.cgi?lvl=0&id=9606)

[gi|283807029](http://www.ncbi.nlm.nih.gov/entrez/query.fcgi?cmd=search&db=protein&doptcmdl=genpept&tool=mascot&term=283807029) from [Homo sapiens](http://www.ncbi.nlm.nih.gov/Taxonomy/Browser/wwwtax.cgi?lvl=0&id=9606)

[gi|283807030](http://www.ncbi.nlm.nih.gov/entrez/query.fcgi?cmd=search&db=protein&doptcmdl=genpept&tool=mascot&term=283807030) from [Homo sapiens](http://www.ncbi.nlm.nih.gov/Taxonomy/Browser/wwwtax.cgi?lvl=0&id=9606)

[gi|283807031](http://www.ncbi.nlm.nih.gov/entrez/query.fcgi?cmd=search&db=protein&doptcmdl=genpept&tool=mascot&term=283807031) from [Homo sapiens](http://www.ncbi.nlm.nih.gov/Taxonomy/Browser/wwwtax.cgi?lvl=0&id=9606)

[gi|283807032](http://www.ncbi.nlm.nih.gov/entrez/query.fcgi?cmd=search&db=protein&doptcmdl=genpept&tool=mascot&term=283807032) from [Homo sapiens](http://www.ncbi.nlm.nih.gov/Taxonomy/Browser/wwwtax.cgi?lvl=0&id=9606)

[gi|283807033](http://www.ncbi.nlm.nih.gov/entrez/query.fcgi?cmd=search&db=protein&doptcmdl=genpept&tool=mascot&term=283807033) from [Homo sapiens](http://www.ncbi.nlm.nih.gov/Taxonomy/Browser/wwwtax.cgi?lvl=0&id=9606)

[gi|283807034](http://www.ncbi.nlm.nih.gov/entrez/query.fcgi?cmd=search&db=protein&doptcmdl=genpept&tool=mascot&term=283807034) from [Homo sapiens](http://www.ncbi.nlm.nih.gov/Taxonomy/Browser/wwwtax.cgi?lvl=0&id=9606)

[gi|283807035](http://www.ncbi.nlm.nih.gov/entrez/query.fcgi?cmd=search&db=protein&doptcmdl=genpept&tool=mascot&term=283807035) from [Homo sapiens](http://www.ncbi.nlm.nih.gov/Taxonomy/Browser/wwwtax.cgi?lvl=0&id=9606)

[gi|283807036](http://www.ncbi.nlm.nih.gov/entrez/query.fcgi?cmd=search&db=protein&doptcmdl=genpept&tool=mascot&term=283807036) from [Homo sapiens](http://www.ncbi.nlm.nih.gov/Taxonomy/Browser/wwwtax.cgi?lvl=0&id=9606)

[gi|283807037](http://www.ncbi.nlm.nih.gov/entrez/query.fcgi?cmd=search&db=protein&doptcmdl=genpept&tool=mascot&term=283807037) from [Homo sapiens](http://www.ncbi.nlm.nih.gov/Taxonomy/Browser/wwwtax.cgi?lvl=0&id=9606)

[gi|291463532](http://www.ncbi.nlm.nih.gov/entrez/query.fcgi?cmd=search&db=protein&doptcmdl=genpept&tool=mascot&term=291463532) from [Homo sapiens](http://www.ncbi.nlm.nih.gov/Taxonomy/Browser/wwwtax.cgi?lvl=0&id=9606)

[gi|291463533](http://www.ncbi.nlm.nih.gov/entrez/query.fcgi?cmd=search&db=protein&doptcmdl=genpept&tool=mascot&term=291463533) from [Homo sapiens](http://www.ncbi.nlm.nih.gov/Taxonomy/Browser/wwwtax.cgi?lvl=0&id=9606)

[gi|223688](http://www.ncbi.nlm.nih.gov/entrez/query.fcgi?cmd=search&db=protein&doptcmdl=genpept&tool=mascot&term=223688) from [Homo sapiens](http://www.ncbi.nlm.nih.gov/Taxonomy/Browser/wwwtax.cgi?lvl=0&id=9606)

[gi|224263](http://www.ncbi.nlm.nih.gov/entrez/query.fcgi?cmd=search&db=protein&doptcmdl=genpept&tool=mascot&term=224263) from [Homo sapiens](http://www.ncbi.nlm.nih.gov/Taxonomy/Browser/wwwtax.cgi?lvl=0&id=9606)

Fixed modifications: Carbamidomethyl (C)

Variable modifications: Oxidation (M)

Cleavage by Trypsin: cuts C-term side of KR unless next residue is P

Sequence Coverage: **38%**

Matched peptides shown in **Bold Red**

**1** GPTGTGESKC PLMVKVLDAV R**GSPAINVAV HVFRKAADDT WEPFASGKTS**

**51 ESGELHGLTT EEEFVEGIYK** VEIDTKSYWK ALGISPFHEH AEVVFTANDS

**101** GPRRYTIAAL LSPYSYSTTA VVTNPKE

  Residue Number  Increasing Mass  Decreasing Mass

**Start - End Observed Mr(expt) Mr(calc) Delta Miss Sequence**

**22 - 34 683.9003 1365.7861 1365.7517 0.0344 0 R.GSPAINVAVHVFR.K**  ([Ions score 69](http://www.matrixscience.com/cgi/peptide_view.pl?file=../data/20100727/FtemCaaOR.dat&query=610&hit=1&index=gi|443295&px=1&section=5&ave_thresh=43&_ignoreionsscorebelow=0&report=5&_sigthreshold=0.05&_msresflags=1089&_msresflags2=2&percolate=-1&percolate_rt=0))

**22 - 35 747.9481 1493.8816 1493.8467 0.0349 1 R.GSPAINVAVHVFRK.A**  ([Ions score 54](http://www.matrixscience.com/cgi/peptide_view.pl?file=../data/20100727/FtemCaaOR.dat&query=663&hit=1&index=gi|443295&px=1&section=5&ave_thresh=43&_ignoreionsscorebelow=0&report=5&_sigthreshold=0.05&_msresflags=1089&_msresflags2=2&percolate=-1&percolate_rt=0))

**35 - 48 761.8731 1521.7317 1521.7100 0.0217 1 R.KAADDTWEPFASGK.T**  ([Ions score 97](http://www.matrixscience.com/cgi/peptide_view.pl?file=../data/20100727/FtemCaaOR.dat&query=671&hit=1&index=gi|443295&px=1&section=5&ave_thresh=43&_ignoreionsscorebelow=0&report=5&_sigthreshold=0.05&_msresflags=1089&_msresflags2=2&percolate=-1&percolate_rt=0))

**36 - 48 697.8268 1393.6391 1393.6150 0.0241 0 K.AADDTWEPFASGK.T**  ([Ions score 75](http://www.matrixscience.com/cgi/peptide_view.pl?file=../data/20100727/FtemCaaOR.dat&query=622&hit=1&index=gi|443295&px=1&section=5&ave_thresh=43&_ignoreionsscorebelow=0&report=5&_sigthreshold=0.05&_msresflags=1089&_msresflags2=2&percolate=-1&percolate_rt=0))

**49 - 70 819.4029 2455.1869 2454.1438 1.0431 0 K.TSESGELHGLTTEEEFVEGIYK.V**  ([Ions score 64](http://www.matrixscience.com/cgi/peptide_view.pl?file=../data/20100727/FtemCaaOR.dat&query=730&hit=1&index=gi|443295&px=1&section=5&ave_thresh=43&_ignoreionsscorebelow=0&report=5&_sigthreshold=0.05&_msresflags=1089&_msresflags2=2&percolate=-1&percolate_rt=0))

Bottom of Form

Bottom of Form
